# A randomised controlled trial of rosuvastatin for the prevention of aminoglycoside-induced kidney toxicity in children with cystic fibrosis

**DOI:** 10.1038/s41598-020-58790-1

**Published:** 2020-02-04

**Authors:** Stephen J. McWilliam, Anna Rosala-Hallas, Ashley P. Jones, Victoria Shaw, William Greenhalf, Thomas Jaki, Alan R. Smyth, Rosalind L. Smyth, Munir Pirmohamed

**Affiliations:** 1 Department of Women’s and Children’s Health, University of Liverpool, Liverpool, Merseyside United Kingdom; 2 Clinical Trials Research Centre, University of Liverpool, a member of the Liverpool Health Partners, Liverpool, Merseyside United Kingdom; 3 Institute of Translational Medicine, University of Liverpool, Liverpool, Merseyside United Kingdom; 4 Department of Mathematics and Statistics, Lancaster University Lancaster, United Kingdom; 5 Division of Child Health, Obstetrics & Gynaecology, University of Nottingham, Nottingham, United Kingdom; 6 University College London, Great Ormond Street Institute of Child Health, London, United Kingdom; 7 Department of Molecular and Clinical Pharmacology, and MRC Centre for Drug Safety Science, University of Liverpool, Liverpool, Merseyside United Kingdom

**Keywords:** Predictive markers, Toxin-induced nephropathy, Cystic fibrosis, Paediatric research, Acute kidney injury

## Abstract

The PROteKT study tested the hypothesis that rosuvastatin can inhibit aminoglycoside-induced nephrotoxicity in children with Cystic Fibrosis (CF). This open label, parallel group, randomised controlled trial recruited children and young people aged 6 to 18 years with CF at 13 paediatric CF treatment centres in the UK. Participants were randomised equally to either receive oral rosuvastatin (10 mg once daily) or no intervention (control) throughout clinically indicated treatment with intravenous tobramycin. The primary outcome was the difference between the groups in mean fold-change in urinary Kidney Injury Molecule-1 (KIM-1). Fifty (rosuvastatin n = 23, control n = 27) participants were recruited between May 2015 and January 2017. Primary outcome data was available for 88% (rosuvastatin n = 20, control n = 24). The estimated mean treatment difference in the geometric mean-fold change of normalised KIM-1 was 1.08 (95% CI 0.87–1.35, p = 0.48). In total there were 12 adverse reactions, all mild, reported by five participants randomised to rosuvastatin, and one serious adverse event in each group. Whilst no protective effect of rosuvastatin was seen, there was a lower than expected level of nephrotoxicity in the cohort. Therefore, we can neither confirm nor refute the hypothesis that rosuvastatin protects against aminoglycoside nephrotoxicity.

## Introduction

In Cystic fibrosis (CF) the resistant pathogen *Pseudomonas aeruginosa* is commonly implicated in secondary bacterial lung infections and colonisation. Aminoglycoside antibiotics, usually combined with a beta-lactam, are frequently used to treat respiratory exacerbations in CF^1^, as they are effective against *P. aeruginosa*. Whilst this approach generally leads to improved patient outcomes, aminoglycoside use is also associated with an increased risk of nephrotoxicity.

The incidence of acute kidney injury (AKI) in children with CF is increased with current or recent exposure to aminoglycosides^2–4^, with rates of 20% reported using daily monitoring of serum creatinine^5^. The risk of AKI is higher with longer duration of aminoglycoside use and with recent previous aminoglycoside exposure^6^. In one cohort of adults with CF, between 31% and 42% had evidence of chronic renal impairment which was significantly associated with cumulative aminoglycoside exposure^7^. However, this has not been replicated in other cohorts^8,9^. Strategies such as therapeutic drug monitoring and extended-interval dosing^10^ are only partially effective in preventing aminoglycoside-induced nephrotoxicity. It is therefore important to develop further, more effective, strategies.

Aminoglycosides cause targeted toxicity to renal proximal tubule epithelial cells. Megalin, a multi-ligand receptor, facilitates the endocytosis and accumulation of aminoglycosides in these cells^11^. This pathway is activated by intermediates derived from mevalonate, which is formed from 3-hydroxy-3-methylglutaryl-coenzyme A (HMG-CoA) via the enzyme HMG-CoA reductase^12^. We hypothesised that inhibiting HMG-CoA reductase with a statin would lead to a reduction in toxicity. Statins are inhibitors of megalin-mediated endocytosis *in vitro*^13,14^, and are therefore likely to reduce uptake of aminoglycosides in the proximal tubule. Indeed, *in vivo* studies of aminoglycoside-induced nephrotoxicity in rats have consistently demonstrated a protective effect of statins^15–20^.

In this paper, we describe the first clinical trial to evaluate the repurposing of a statin for the prevention of aminoglycoside-induced kidney toxicity. Rosuvastatin was chosen as it is hydrophilic, has minimal hepatic metabolism, and has some renal elimination of the parent drug^21^ (and therefore theoretically preferable in preventing megalin-mediated uptake rather than a hepatically metabolised statin). In addition, it is highly potent, and licensed for use in children. This trial was designed to test the hypothesis that rosuvastatin can inhibit aminoglycoside-induced nephrotoxicity in children with CF.

## Results

### Study participants

**Figure 1:**
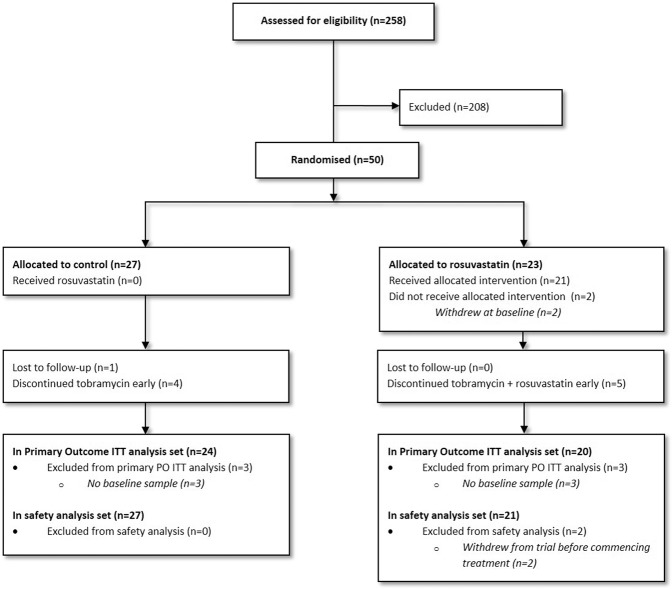
CONSORT Flow Diagram.

A total of 258 patients were assessed for eligibility across 13 CF centres in the UK (May 2015-January 2017); 208 were not randomised as they failed to meet the inclusion/exclusion criteria or did not provide consent. The planned sample size was met with twenty-three participants randomised to the intervention and 27 to the control (Fig. 1). Baseline characteristics are shown in Table 1. The two arms were well balanced and no clinical differences were deemed to be important.

**Table 1:** Characteristics of the participants at baseline.

Characteristic	Control Group (n = 27)	Intervention Group (n = 23)
Female sex – no. (%)	19 (70)	13 (57)
Age - yr	13.30 (2.65)	12.09 (2.74)
Height – cm	151.93 (16.07)	148.43 (915.79)
Weight - kg	44.67 (15.58)	40.63 (14.98)
Ethnic origin – no. (%)		
White	27 (100)	21 (91)
Other White	0 (0)	1 (4)
Mixed: White and Black African	0 (0)	1 (4)
**Blood Results**		
Serum creatinine - µmol/L	45.30 (10.56)	43.57 (11.52)
eGFR - mL/min/1.73 m^2^	139.90 (29.69)	142.21 (27.02)
Aspartate transaminase - iu/L	28.77 (11.09)^a^	33.41 (17.69)^a^
Alanine transaminase - iu/L	25.48 (16.53)	27.77 (17.09)
HDL cholesterol - mmol/L	1.06 (0.32)	1.09 (0.40)
LDL cholesterol - mmol/L	1.47 (0.54)^a^	1.25 (0.55)
Total cholesterol- mmol/L	2.80 (0.67)	2.75 (0.66)
Triglycerides - mmol/L	1.17 (0.77)	1.00 (0.53)
Creatine kinase - iu/L	67.15 (33.13)	83.83 (55.31)
C Reactive Protein - mg/L	10.46 (14.29)	7.48 (8.41)
**Spirometry Results**		
FEV in 1 second	2.10 (1.30)	1.86 (0.91)^b^
FEV in 1 second (% predicted)	74.05 (17.57)	73.98 (19.59)^b^
**Urine Results**		
KIM-1 (normalised to urinary creatinine) -ng/mgCr	1.94 (2.45)^c^	0.67 (0.45)^c^
NGAL (normalised to urinary creatinine) - ng/mgCr	61.08 (89.55)^c^	22.46 (22.99)^c^

*Plus-minus values are means ± SD ^a^Measurement missing for 1 participant. ^b^Measurement missing for 2 participants. ^c^Measurement missing for 3 participants.

Five (22%) and four (15%) participants randomised to the intervention and control groups, respectively, discontinued tobramycin treatment early. Median duration of treatment was 14 days in both the intervention and control groups (Supplemental Table 1). Four participants in the intervention group discontinued tobramycin treatment due to a change in their condition and one due to line failure. One control group participant discontinued tobramycin treatment early due to a change in their condition, two were a clinical decision to cease and, in one case, no reason was given.

### Assessment of outcome measures

**Figure 2:**
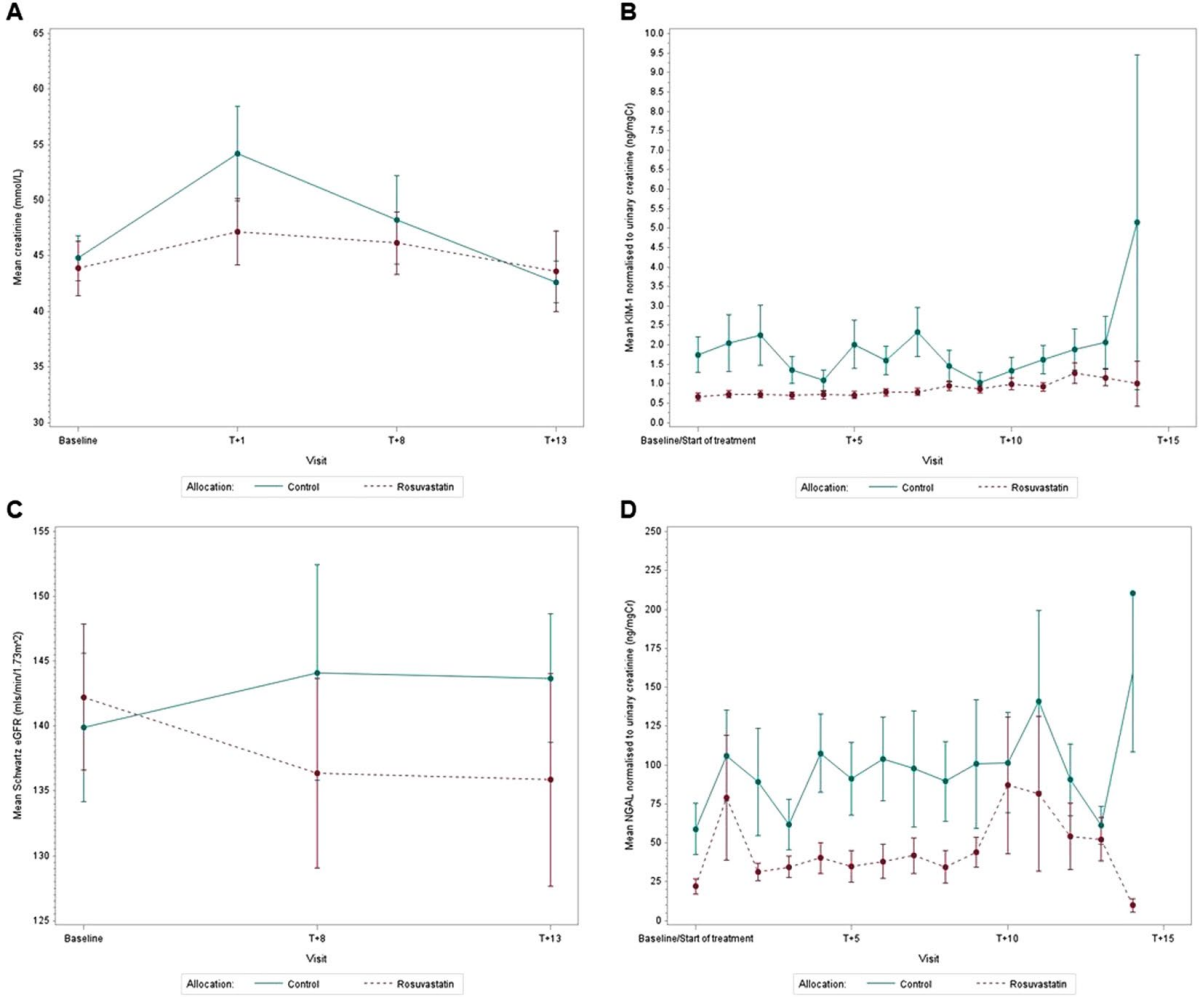
Mean profile plots. Mean profile plots for control and intervention (rosuvastatin) groups during tobramycin exposure for creatinine (**A**), KIM-1 normalised to urinary creatinine (**B**), Schwartz eGFR (**C**), and NGAL normalised to urinary creatinine (**D**).

In terms of the primary outcome, the estimated geometric mean fold-change of normalised Kidney Injury Molecule-1 (KIM-1) was 1.85 and 2.00 in the control and intervention groups respectively (Table 2). The estimated mean treatment difference was 1.08 (95% CI: 0.87,1.35; p = 0.48). Four participants (intervention n = 1 and control n = 3) did not have baseline urine samples and were excluded from the primary analysis. Five sensitivity analyses confirmed the conclusion found in the primary analysis (Supplemental Table 2). The estimated mean treatment difference in the area under the curve (AUC) of normalised KIM-1 between the two treatment groups was 12.41 ng/mgCr (95% CI: −0.89,25.70; p = 0.07) (Supplemental Table 3). There were no statistically significant differences between the treatment groups and the treatment by time interactions were not significant for the secondary outcomes (Table 3). Mean profile plots for each outcome measure are included in Fig. 2.

**Table 2:** Primary outcome: ANCOVA model results.

Primary outcome: log-transformed mean fold change from baseline to peak KIM-1 normalised to urinary creatinine	N^a^	Estimated geometric mean fold-change	Estimated mean treatment difference^b^	95% CI	P-value
Control	24	1.85	—	—	—
Rosuvastatin	20	2.00	1.08	0.87, 1.35	0.48

^a^3 in the control and 1 in the intervention arm did not have baseline urine samples and were excluded; 2 in the intervention arm withdrew at baseline before commencing treatment and were excluded. ^b^Adjusted for baseline normalised KIM-1.

**Table 3:** Secondary outcomes: random intercept model results.

Secondary Outcomes	Treatment group	N	Estimated mean	Estimated mean treatment difference	95% CI	P-value
Serum creatinine (μmol/l)	Control	27	47.61	—	—	—
	Rosuvastatin	23	44.66	−2.95	−9.61, 3.71	0.38
eGFR (mls/min/1.73 m^2^)	Control	27	142.01	—	—	—
	Rosuvastatin	23	140.59	−1.43	−16.64, 13.78	0.85
NGAL (ng/mgCr)	Control	27	101.75	—	—	—
	Rosuvastatin	21^a^	47.04	−54.71	−102.25, −7.16	0.02
FEV in 1 second (L)	Control	27	1.94	—	—	—
	Rosuvastatin	21^b^	1.93	−0.01	−0.50, 0.49	0.98
CRP (mg/L)	Control	27	9	—	—	—
	Rosuvastatin	23	5.89	−3.11	−7.68, 1.46	0.18

^a^2 participants withdrew at baseline prior to commencing treatment thus had no samples to contribute. ^b^2 participants withdrew at baseline and had missing FEV1 samples at baseline.

The estimated geometric mean fold-change of normalised Neutrophil Gelatinase-Associated Lipocalin (NGAL) was 8.90 and 4.99 in the control and intervention groups respectively. The estimated mean treatment difference was 0.56 (95% CI: 0.27,1.15; p = 0.11) (Supplemental Table 4). The estimated mean treatment difference in the AUC of normalised NGAL between the two arms was 557.8 ng/mgCr (95% CI: 46.5,1069.2; p = 0.03) (Supplemental Table 3). The tobramycin and rosuvastatin data are reported in Supplemental Tables 5 and 6, respectively.

An analysis of serum creatinine data using the Kidney disease: Improving global outcomes (KDIGO) AKI criteria^22^ demonstrated that a total of four participants fulfilled the criteria for AKI during the trial: two patients in the control group both reached AKI stage 3, and two patients in the intervention group both reached AKI stage 1 (Supplemental Table 7).

### Safety and adverse events

Two participants randomised to the intervention group withdrew consent prior to receiving the intervention and were therefore not included in the safety analysis. Twelve adverse reactions were reported by 5 (25%) of the 21 participants in the intervention group (Table 4). All events were reported as mild in severity.

**Table 4:** Adverse Reactions reported in the intervention group (n = 21).

Event	Number of participants (%)	Number of events
**Metabolism and nutrition disorders**		
Hypoglycaemia	1 (4.76%)	2
**Investigations**		
Alanine aminotransferase increased	1 (4.76%)	1
Aspartate aminotransferase increased	1 (4.76%)	1
Blood cholesterol decreased	1 (4.76%)	1
Blood creatine phosphokinase increased	1 (4.76%)	1
Blood triglycerides decreased	1 (4.76%)	1
Blood triglycerides increased	1 (4.76%)	1
**Musculoskeletal and connective tissue disorders**		
Back pain	1 (4.76%)	1
**Nervous system disorders**		
Headache	1 (4.76%)	1
Paraesthesia	1 (4.76%)	1
Paraesthesia oral	1 (4.76%)	1

Recurrence of pulmonary exacerbation during follow-up, requiring hospitalisation, was the only serious adverse event in the control group, reported for one (4%) participant. Abnormal blood results requiring prolonged hospitalisation were reported as a serious adverse event for one (5%) participant in the intervention group but this was not considered to be related to the intervention.

## Discussion

This is the first randomised controlled trial in man to assess whether rosuvastatin has a protective effect against tobramycin-induced nephrotoxicity. It was designed as an early phase trial using KIM-1 as a surrogate outcome measure. We found little evidence of a protective effect of rosuvastatin in this trial as determined by both primary and secondary outcomes, but this should be interpreted with caution as the control group only demonstrated a mean fold-change of KIM-1 of 1.85. The sample size calculation for this study was, however, based upon an expected mean fold-change in KIM-1 of 3.03 during exposure to tobramycin derived from an early analysis of samples in the URBAN CF study^23^. The trial was powered to detect a difference in fold-change between the groups of 2. The PROteKT and URBAN CF populations were similar, although PROteKT contained a greater proportion of female participants, and had higher mean age, height and weight (due to differences in the inclusion criteria for age). There were no differences in aminoglycoside prescribing practices between the two studies. We do not believe that the differences between the populations used in PROteKT and URBAN CF account for lower than expected levels of nephrotoxicity observed in our randomised controlled trial. Therefore, it is unclear whether this reflects lower than usual levels of toxicity in the PROteKT cohort, or higher than usual levels of toxicity in the cohort upon which the sample size calculation was based. This makes it difficult to definitively exclude the possibility of a protective effect of rosuvastatin. Indeed, some of our secondary outcome measures suggest a trend towards a protective effect. Importantly, the use of rosuvastatin was generally well tolerated.

Urine KIM-1 is a cell membrane glycoprotein upregulated by proximal tubule epithelial cells in response to toxicity^24^. It was chosen as the primary outcome measure as it has previously shown potential for the identification of aminoglycoside-induced nephrotoxicity in preterm neonates^25^, and in children with CF^23,26^, as well as outperforming other biomarkers of aminoglycoside-induced nephrotoxicity in pre-clinical models^27^. In clinical practice, changes in serum creatinine meeting agreed criteria for AKI^22^ are more widely accepted for defining nephrotoxicity. However, changes in serum creatinine are a relatively late event in the process of nephrotoxicity, and for the purposes of a phase IIa trial, it was felt that KIM-1 was a good surrogate measure. Indeed, we identified no significant treatment effect upon serum creatinine in this study, consistent with our previous experience in the URBAN CF study^23^. The use of a change in serum creatinine as an outcome measure may be relevant to use in future trials, but given that this is estimated to occur in 14–20%^5,6^, a much larger sample size will be required.

Urine NGAL is a 25 kDa protein expressed by kidney epithelial cells (as well as other tissues, including neutrophils)^28^. It is a sensitive predictor for AKI^29^, and has previously been shown to be elevated during aminoglycoside exposure in neonates^25^, and in children with CF^23^. Patients in the intervention group had a smaller estimated geometric mean fold-change of normalised NGAL (p = 0.11), and a significantly lower AUC of normalised NGAL. The difference in AUC between the groups must be interpreted in the light of a higher baseline urinary NGAL in the control compared to the intervention group (61.08 ng/mgCr and 22.46 ng/mgCr respectively). This may, in part, be explained by the higher proportion of females in the control versus intervention groups as females have been consistently shown to have higher baseline NGAL concentrations than males^30^. Whilst our results may be interpreted to suggest a protective effect of rosuvastatin, we feel that these should be interpreted cautiously.

Published studies in rat models of aminoglycoside-induced nephrotoxicity have consistently demonstrated a protective effect with a range of statins, including atorvastatin^17–19^, simvastatin^15,16^ and rosuvastatin^20^. We felt that rosuvastatin was the most promising candidate based upon its pharmacology. It is a hydrophilic statin, and has greater renal excretion than the more lipophilic atorvastatin and simvastatin. It causes more proteinuria than other statins, probably because it is secreted in the proximal tubules^31^ and inhibits megalin-mediated endocytosis. We considered whether we should have given the statin for a few days in advance of commencing the tobramycin in order to achieve steady-state concentrations. However, published pre-clinical studies demonstrated a protective effect of statins without the need for this^15,17–20^.

The recruitment of children with CF from thirteen different sites in the UK to participate in this trial ensures that differences in practice between centres should be accounted for in the randomisation. The trial was designed to cause the least disruption possible to standard daily care, and for the intervention to be minimally burdensome to patients (requiring only one additional medicine per day for 2 weeks). This was reflected in the good adherence to the randomised intervention, and by a low number of reported adverse reactions.

In conclusion, given the lower than expected level of nephrotoxicity seen in this study we feel that we can neither confirm nor refute the hypothesis that statins protect against aminoglycoside nephrotoxicity. Given the large amount of pre-clinical data which shows that statins can be protective^15–20^, we feel further studies in humans are warranted. These would need to be powered for a clinically acceptable primary outcome measure such as AKI (rise in creatinine). There are of course other interventions which have been attempted to reduce the risk of aminoglycoside nephrotoxicity, including extended-interval dosing and drug trough level monitoring^10^, and morning (versus evening) administration of the aminoglycoside^32^. All these interventions are not mutually exclusive, and it may be that combinations of interventions may be more successful in preventing renal injury when compared with individual interventions, and such a possibility should be investigated in an appropriately designed clinical trial in the future.

## Methods

### Protocol and trial population

This multi-centre, randomised, open-labelled, parallel group, phase IIa trial was registered in duplicate on both the EU Clinical Trials Register (EudraCT number 2014-002387-32, 27/06/2014) and the ISRCTN Registry (ISRCTN26104255, 05/09/2014). It received ethical approval from the National Research Ethics Service Committee North West – Greater Manchester Central, and was conducted in accordance with the Declaration of Helsinki. Participants were recruited at 13 paediatric CF treatment centres in the UK. Each parent or guardian gave written informed consent, and each child gave assent where appropriate.

Children and young people with CF, aged 6 to 18 years, who had a planned, clinically indicated (usually for a respiratory exacerbation of CF), course of treatment with IV tobramycin were eligible for randomisation. Key exclusion criteria included participants of Asian ancestry (specifically Japanese, Chinese, Filipino, Vietnamese and Korean subjects, as they have clinically significant increased systemic exposure to rosuvastatin^33^), previous adverse reaction to a statin, and existing treatment with a statin. Participants with renal disease, elevation of either transaminases and/or creatine kinase were also excluded. The full inclusion and exclusion criteria are described in the trial protocol (Appendix 1).

There were amendments to the eligibility criteria during the trial. The age inclusion criteria were changed from 10–18 to 6–18 years following a change in the licence for rosuvastatin^34^. A review of published pharmacokinetic data suggested that concomitant itraconazole^35^ or Asian-Indian ethnicity^33^ would result in clinically insignificant increases in rosuvastatin exposure and therefore these were removed from the exclusion criteria.

### Randomisation and trial procedures

Randomisation was performed, by the Principal Investigator or delegated other at site, using a secure web-based system with random permuted blocks of 2 and 4 stratified by trial site. Randomisation lists were generated using Stata v9.0 by an independent statistician. Emergency back-up randomisation envelopes were available when there was a problem with this system. At enrolment eligible participants were randomised 1:1 to receive either rosuvastatin or no intervention.

Each participant randomised to the intervention arm received a daily oral dose of 10 mg rosuvastatin (Crestor®; AstraZeneca UK Ltd) for the duration of a treatment course of IV tobramycin (usually 14 days). Tobramycin was the first choice aminoglycoside in CF for all centres, and dosing and monitoring of drug concentrations followed the British National Formulary^36^ (standard dosing is 10 mg/kg once daily) and local protocols. The first dose of rosuvastatin was given on the same calendar day and prior to the first dose of IV tobramycin. Subsequent daily doses of rosuvastatin were given prior to that day’s dose of tobramycin. Treatment with oral rosuvastatin continued for the duration of the course of IV tobramycin. If the tobramycin course was shorter than 14 days, rosuvastatin was discontinued after the final tobramycin dose. If the tobramycin course was longer than 14 days, rosuvastatin was continued until the final day of the tobramycin course. Participants randomised to the no intervention arm received usual care during their IV tobramycin treatment course. No placebo was provided.

### Sample collection

Baseline assessment before tobramycin therapy (T0) included collection of urine and blood samples for confirmation of eligibility criteria, and spirometry. During treatment with IV tobramycin urine samples were collected daily from each child. Participants had assessments on days T + 1, T + 8 and T + 13. These included adverse event assessment, a symptom-directed physical examination if required, and collection of blood and urine samples. Spirometry was performed at the T + 8 and T + 13 visits. A final follow-up visit, with collection of blood/urine samples and spirometry, was conducted 3–5 weeks following completion of tobramycin treatment.

Urine samples were normally collected by clean catch into a sterile container. If the participant was an inpatient, the daily samples were sent directly to the local laboratory. If the participant received IV tobramycin at home, samples were stored in the home refrigerator in a sealed container until the next scheduled study visit. All samples were stored at fridge temperature (4 °C) for a total of one week (including time spent at the patient’s home) and then aliquoted and stored at −80 °C (or −20 °C for a maximum of 6 months) in the local laboratory. Stability of KIM-1 has previously been reported in urine stored at 4 °C for periods of one week^37,38^.

Wherever possible, study bloods were collected by venepuncture at the same time as any clinically indicated bloods in order to minimise the burden to the patient. A minimum of two 1.2 ml samples in Lithium/Heparin tubes, and one 1.2 ml sample in a serum tube were collected at each time point. Plasma and serum were extracted locally at each site, aliquoted and stored at −80 °C.

Batched samples were couriered on dry ice to the Good Clinical Practice Laboratory (GCPLab) facility in Liverpool for subsequent storage and analysis (here samples were stored at −80 °C). All laboratory analyses were undertaken in a standardised manner, being blinded to allocation, time of sampling and clinical outcomes of patients.

### Outcome measures

The primary outcome was the difference in mean fold-change in urinary KIM-1 from baseline to ‘highest value’ concentration during exposure to tobramycin between the intervention and control arms.

The secondary outcome measures were the difference in serum creatinine, estimated glomerular filtration rate (eGFR) and urinary NGAL between the intervention and control arms during exposure to tobramycin. We assessed for pharmacokinetic interaction between rosuvastatin and tobramycin by comparing tobramycin concentrations between the rosuvastatin treated arm and the control arm. We assessed for pharmacodynamic interaction between rosuvastatin and tobramycin, by comparing change in percent of predicted Forced Expiratory Volume in 1 second (FEV1), between the rosuvastatin treated and control arms. This is a widely used indirect measure of aminoglycoside treatment outcome in children with CF^39^, and was measured locally during study visits. We also compared change in C-Reactive Protein (CRP), as a marker of inflammation/infection, between the two groups. Rosuvastatin concentrations were measured in the intervention arm participants to describe the pharmacokinetic profile of rosuvastatin, to assess compliance, and to relate rosuvastatin concentrations to change in urinary KIM-1.

### Safety

All the participants who received at least one dose of rosuvastatin were included in the safety analysis. Adverse reactions and serious adverse events were recorded from the point of informed consent and throughout the trial treatment period up until the final assessment (3–5 weeks after the participant had taken the final dose of rosuvastatin).

### Laboratory analyses

Urinary KIM-1 and NGAL were measured using validated electrochemiluminescent assays (Meso Scale Discovery (MSD), US) as previously described^23,30^. Biomarker values were normalised to urinary creatinine which was determined spectrophotometically as previously described^40^. Serum creatinine, tobramycin concentrations and CRP were measured in the laboratory serving the local hospital site.

Rosuvastatin concentrations were measured centrally at the University of Liverpool. A high performance liquid chromatography mass spectrometric method for the estimation of rosuvastatin in plasma, was developed and validated using rosuvastatin-D6 as internal standard. Sample preparation was accomplished by protein precipitation. The samples were chromatographed on a C-18 Halo column using mobile phases consisting of 0.1% formic acid in water and 0.1% formic acid in acetonitrile. The method was validated over a concentration range of 0.5–100 ng/mL for rosuvastatin.

### Statistical analysis

The statistical analysis plan (Appendix 2) was written prior to any formal analysis. The trial was designed with a 92% power to detect a difference in fold-change in KIM-1 between the 2 groups at a 5% significance level (two sided). A standard deviation of 1.84 was derived from an early analysis of samples in the URBAN CF study^23^ from 10 participants receiving a single course of treatment with tobramycin. The same data was also inspected to assess that the assumption of normality is reasonable. Using these assumptions and utilizing a 2-sample t-test, a sample size of 20 in each arm would be sufficient. We planned to include 50 patients in order to compensate for 10% loss to follow up.

The principle of intention-to-treat was the main strategy of the analysis adopted for the primary outcome and all the secondary outcomes. These analyses were conducted on all randomised participants, in the group to which they were allocated, and for whom the outcome(s) of interest have been observed/measured.

The primary outcome was analysed using the method of analysis of covariance (ANCOVA). The outcome measure was the mean log-transformed fold-change of normalised KIM-1 (ng/mgCr) calculated by dividing the peak value, corresponding to the ‘highest value’ of normalised KIM-1 during exposure to tobramycin, by the baseline normalised KIM-1 value for each participant. The explanatory variables were treatment group and baseline normalised KIM-1 value. 95% confidence intervals will be reported and a p-value of <0.05 considered statistically significant. The secondary outcomes were analysed longitudinally using random intercept models with unstructured covariance matrices, including an interaction between treatment group and visit.

In an additional analysis of normalised NGAL (ng/mgCr), an ANCOVA model was used, as for KIM-1 above, comparing log-transformed mean fold-change from baseline to peak NGAL between the treatment groups, controlling for the baseline normalised NGAL. The model estimates were exponentiated to be interpretable on the normal scale.

In an additional descriptive analysis of serum creatinine data, change from baseline serum creatinine during tobramycin exposure was defined according to the KDIGO AKI criteria (Stage 1, increase ≥1.5 and <2 times from baseline; Stage 2, increase ≥2 and <3 times from baseline; Stage 3, increase ≥3 times from baseline)^22^.

Pre-specified sensitivity analyses tested impact of missing data; the statistical analysis plan includes full details of sensitivity analyses and analyses of secondary outcomes. Analyses were performed using SAS® version 9.4 (SAS Inc., USA).

## Supplementary information

PROteKT Supplementary Material.

## Data Availability

Data may be requested by submitting a written request to the corresponding author outlining the purposes for which the data would be used.
