# Author Correction: A randomised controlled trial of rosuvastatin for the prevention of aminoglycoside-induced kidney toxicity in children with cystic fibrosis

**DOI:** 10.1038/s41598-020-61783-9

**Published:** 2020-03-10

**Authors:** Stephen J. McWilliam, Anna Rosala-Hallas, Ashley P. Jones, Victoria Shaw, William Greenhalf, Thomas Jaki, Alan R. Smyth, Rosalind L. Smyth, Munir Pirmohamed

**Affiliations:** 1 Department of Women’s and Children’s Health, University of Liverpool, Liverpool, Merseyside United Kingdom; 2 Clinical Trials Research Centre, University of Liverpool, a member of the Liverpool Health Partners, Liverpool, Merseyside United Kingdom; 3 Institute of Translational Medicine, University of Liverpool, Liverpool, Merseyside United Kingdom; 4 Department of Mathematics and Statistics, Lancaster University, Lancaster, United Kingdom; 5 Division of Child Health, Obstetrics & Gynaecology, University of Nottingham, Nottingham, United Kingdom; 6 University College London, Great Ormond Street Institute of Child Health, London, United Kingdom; 7 Department of Molecular and Clinical Pharmacology, and MRC Centre for Drug Safety Science, University of Liverpool, Liverpool, Merseyside United Kingdom

Correction to: *Scientific Reports* 10.1038/s41598-020-58790-1, published online 04 February 2020

In this Article, the Competing Interests statement was omitted. The Competing Interests section should read:

“MP has a PhD studentship jointly funded by EPSRC and Astra Zeneca on drug-drug interactions, but this is not specifically focusing on rosuvastatin or other statins.”

