# Supplementary material for: A randomised controlled trial of rosuvastatin for the prevention of aminoglycoside-induced kidney toxicity in children with cystic fibrosis
Source: Sci Rep. 2020 Feb 4;10:1796. doi: 10.1038/s41598-020-58790-1 (PMC7000680; doi:10.1038/s41598-020-58790-1)
Supplement: Supplementary file 1 — PROteKT Supplementary Material. [file 41598_2020_58790_MOESM1_ESM.pdf]

# A randomised controlled trial of rosuvastatin for the prevention of aminoglycoside-induced kidney toxicity in children with cystic fibrosis

**Stephen J McWilliam PhD, Anna Rosala-Hallas MSc, Ashley P Jones PhD, Victoria Shaw PhD, William Greenhalf PhD, Thomas Jaki PhD, Alan R Smyth MD, Rosalind L Smyth MD, Munir Pirmohamed PhD**

## Supplemental Material

# Contents

Appendix 1: PROteKT Protocol - Version 8, 6th February 2017 .......... 2

Appendix 2: Statistical Analysis Plan – Version 4.0, 28th March 2018 .......... 63

Supplemental Tables .......... 89

Supplemental Table 1: Treatment compliance .......... 89

Supplemental Table 2: Sensitivity analyses for the primary outcome .......... 89

Supplemental Table 3: Area under the curve analysis results .......... 91

Supplemental Table 4: NGAL ANCOVA model results .......... 91

Supplemental Table 5: Post-hoc Analysis: Linear mixed model to assess difference in tobramycin concentrations .......... 91

Supplemental Table 6: Relationship between plasma rosuvastatin concentrations and urinary KIM-1 .......... 92

Supplemental Table 7: AKI by KDIGO criteria .......... 92

## Appendix 1: PROteKT Protocol - Version 8, 6th February 2017

# PROteKT

## Phase IIa, Randomised, Controlled, Open-Label Trial of Rosuvastatin for the Prevention of Aminoglycoside-Induced Kidney Toxicity in Children with Cystic Fibrosis

**Chief Investigator:** Prof Munir Pirmohamed
**Study Sponsor:** The University of Liverpool

**EudraCT number:** **2014-002387-32**
**REC reference:** **14/NW/1067**
**Sponsor reference:** **UoL001019**
**IRAS Project ID:** **137736**

UNIVERSITY OF LIVERPOOL

UCL INSTITUTE OF CHILD HEALTH

# Protocol Approval

**Authorised by Chief Investigator:**

**Signature: ________________________________________ Date: ______________**

Professor Munir Pirmohamed
University of Liverpool
The Wolfson Centre for Personalised Medicine
Department of Molecular and Clinical Pharmacology
Block A: Waterhouse Buildings
1-5 Brownlow Street
Liverpool, L69 3GL
Tel: 0151 794 5549
Fax: 0151 794 5540
E-mail: munirp@liverpool.ac.uk

**Authorised on behalf of the Sponsor(s):**

**Signature: ________________________________________ Date: ______________**

Mr Alex Astor
Research Support Office
2nd Floor Block D Waterhouse Building
3 Brownlow Street
Liverpool
L69 3GL
Tel: 0151 794 8739
Email: sponsor@liv.ac.uk

**Authorised by Statistician:**

**Signature: ________________________________________ Date: ______________**

Dr Ashley Jones
Medicines for Children Clinical Trials Unit
Clinical Trials Research Centre
University of Liverpool
Institute of Child Health
Alder Hey Children's NHS Foundation Trust
Liverpool
L12 2AP

# Contact Details

**Sponsor:**

University of Liverpool
Research Support Office
2nd Floor Block D Waterhouse Building
3 Brownlow Street
Liverpool
L69 3GL
Tel: 0151 7948739
Email: sponsor@liverpool.ac.uk

**Clinical Trials Unit:**
Medicines for Children Clinical Trials Unit
Clinical Trials Research Centre
University of Liverpool
Institute of Child Health
Alder Hey Children's NHS Foundation Trust
Liverpool
L12 2AP

**Trial Manager:**
Cathy MacLean
UCL Comprehensive Clinical Trials Unit
Gower Street
London, WC1E 6BT
protekt_trial@ucl.ac.uk

**Central Laboratories:**
The Wolfson Centre for Personalised Medicine
Department of Molecular and Clinical Pharmacology
Institute of Translational Medicine
University of Liverpool
Block A:Waterhouse Buildings
1-5 Brownlow Street
Liverpool
L69 3GL

**GCLP Laboratory**
Molecular and Clinical Cancer Medicine
5th Floor UCD Block
Royal Liverpool University Hospital
Daulby Street Liverpool
Tel 0151 706 4184
Fax 0151 706 5826

## Personnel Involved

**Professor Munir Pirmohamed**, David Weatherall Chair of Medicine and NHS Chair of Pharmacogenetics, University of Liverpool

**Professor Rosalind Smyth,** Director, Institute of Child Health, University College London

**Dr Daniel Antoine,** Lecturer in Pharmacology, University of Liverpool

**Dr Stephen McWilliam,** MRC Clinical Pharmacology Research Fellow, University of Liverpool

**Dr Ashley Jones**, Senior Statistician, University of Liverpool

## TABLE OF CONTENTS

1 PROTOCOL SUMMARY ........ 7
2 BACKGROUND INFORMATION ........ 10
3 SELECTION OF CENTRES/CLINICIANS ........ 17
4 TRIAL DESIGN ........ 18
5 STUDY POPULATION ........ 21
6 ENROLMENT AND RANDOMISATION ........ 24
7 TRIAL TREATMENT ........ 29
8 ASSESSMENTS AND PROCEDURES ........ 35
9 STATISTICAL CONSIDERATIONS ........ 42
10 PHARMACOVIGILANCE ........ 45
11 ETHICAL CONSIDERATIONS ........ 50
12 TRIAL MONITORING ........ 54
13 RESEARCH GOVERNANCE, SPONSORSHIP AND FINANCIAL ARRANGEMENTS ........ 58
14 REGULATORY APPROVAL ........ 60
15 PUBLICATION ........ 60
16 REFERENCE LIST ........ 61

# 1 PROTOCOL SUMMARY

**Title:** PROteKT
Phase IIa, Randomised, Controlled, Open-Label Trial of Rosuvastatin for the Prevention of Aminoglycoside-Induced Kidney Toxicity in Children with Cystic Fibrosis

**Phase:** IIa

**Study Design:** This study is a phase IIa, multi-centre, randomised, controlled, open-label trial of rosuvastatin in children with cystic fibrosis (CF) receiving clinically indicated treatment with the intravenous (IV) aminoglycoside antibiotic, tobramycin. Patients will be randomised equally to either receive rosuvastatin 10mg once daily or no intervention (control), throughout a course of treatment with IV tobramycin (usually lasting 14 days).

**Study Objectives**

**Primary Objective:** This trial will evaluate the effect of rosuvastatin on aminoglycoside-induced nephrotoxicity. This will be assessed using the difference in mean fold-change in urinary KIM-1 from baseline to 'highest value' concentration during exposure to tobramycin between the rosuvastatin treated arm and control arm.

**Secondary Objectives:**
1. Change in serum concentration of creatinine and eGFR during tobramycin exposure between rosuvastatin treated arm and the control arm.
2. Change in other urinary and plasma biomarkers of renal injury during tobramycin exposure between rosuvastatin treated arm and the control arm.
3. Difference in serious adverse events between rosuvastatin treated arm and the control arm.
4. Difference in tobramycin concentrations between rosuvastatin treated arm and the control arm to identify any pharmacokinetic interaction between rosuvastatin and the tobramycin.
5. Difference in Forced Expiratory Volume in 1 second (FEV1) and C-Reactive Protein, between rosuvastatin treated arm and the control arm to identify any pharmacodynamics interaction between rosuvastatin and the tobramycin
6. Assessment of plasma rosuvastatin concentrations achieved in children randomised to the intervention arm.
7. Difference in biomarkers of *Pseudomonas aeruginosa* between rosuvastatin treated arm and the control arm.
8. Assess the feasibility of collecting DNA for a molecular genetic study of aminoglycoside-induced nephrotoxicity.

**Population:** Children with cystic fibrosis aged 6 to 18 years receiving clinically indicated treatment with intravenous tobramycin, and who fulfil the inclusion criteria.

**Criteria for Inclusion:**
1. Age 6 to 18 years inclusive.
2. Diagnosis of cystic fibrosis (established by sweat test or genotype).
3. Planned, clinically indicated, course of treatment with IV tobramycin.
4. Ability to give informed consent.
5. Willingness to comply with all study requirements.
6. Able to take tablets.

**Criteria for Exclusion:**
1. Existing treatment with a statin.
2. Previous adverse reaction to a statin.
3. Co-enrolment in other drug trials*, or completion of a previous CTIMP within the last 30 days.
4. Previous randomisation in the PROteKT trial.
5. Patients taking any of the following medications: Ciclosporin, Protease Inhibitors, Fibrates, Ezetimibe, Erythromycin (but not other macrolides), Eltrombopag, Dronedarone, - Coumarins, Oral contraceptives, nicotinic acid, fusidic acid and Simepravir.

6. Female participants who are pregnant or lactating or refuse a pregnancy test if of childbearing potential (female participants of childbearing potential must use a barrier method of contraception if sexually active whilst taking rosuvastatin and for 7 days afterwards).
7. Patients of Asian ancestry (Japanese, Chinese, Filipino, Vietnamese andKorean). **†**
8. Patients with renal disease (eGFR<60ml/min/1.73m², using the Schwartz formula**, in the 6 months preceding screening).
9. Patients with current elevation in transaminases exceeding 3x the upper limit of normal, in the past 12 weeks.
10. Patients with current elevation in creatine kinase exceeding 2x the upper limit of normal at baseline, or in the past 12 weeks ***
11. Family history, or personal history, of hereditary muscular disorders.
12. Patients with myopathy.
13. Patients with a history of, or active alcohol abuse.
14. Patients with hypothyroidism.
15. Patients with galactose intolerance, the Lapp lactase deficiency, or glucose-galactose malabsorption.
16. Patients who are Hepatitis C positive or HIV-positive.

***Patients who are currently taking part in TORPEDO-CF are allowed to take part in PROteKT as long as their date of randomisation into TORPEDO-CF is not within the previous six months of the screening date for PROteKT.**

****Schwartz formula to calculate eGFR = [40 * height (cm)] / serum or plasma creatinine (µmol/l)**

***** If creatine kinase in the past 12 weeks exceeds 2x the upper limit of normal this should be confirmed with the baseline bloods. If the baseline creatine kinase exceeds 2x the upper limit of normal this test should be repeated within 6 hours. If the creatine kinase remains elevated above 2x the upper limit of normal then the patient should be excluded. If the repeat creatine kinase is less than 2x the upper limit of normal, the patient may be included.**

**† Patients of Asian-Indian ethnicity may be included as a recent pharmacokinetic study[23] suggests that patients of Asian-Indian ethnicity would experience only about a 30% increase in exposure to rosuvastatin which is unlikely to be clinically significant.**

**Study Centres and Distribution:** Paediatric cystic fibrosis centres in the UK

**Study Duration:** The recruitment period will last 18 months

**Description of Agent/ Intervention:** Oral rosuvastatin 10mg once daily.

**Number of participants to be enrolled:** Up to 50 participants will be enrolled to account for loss to follow-up. A minimum of 20 participants in each arm of the study is required.

## Study Flow Chart

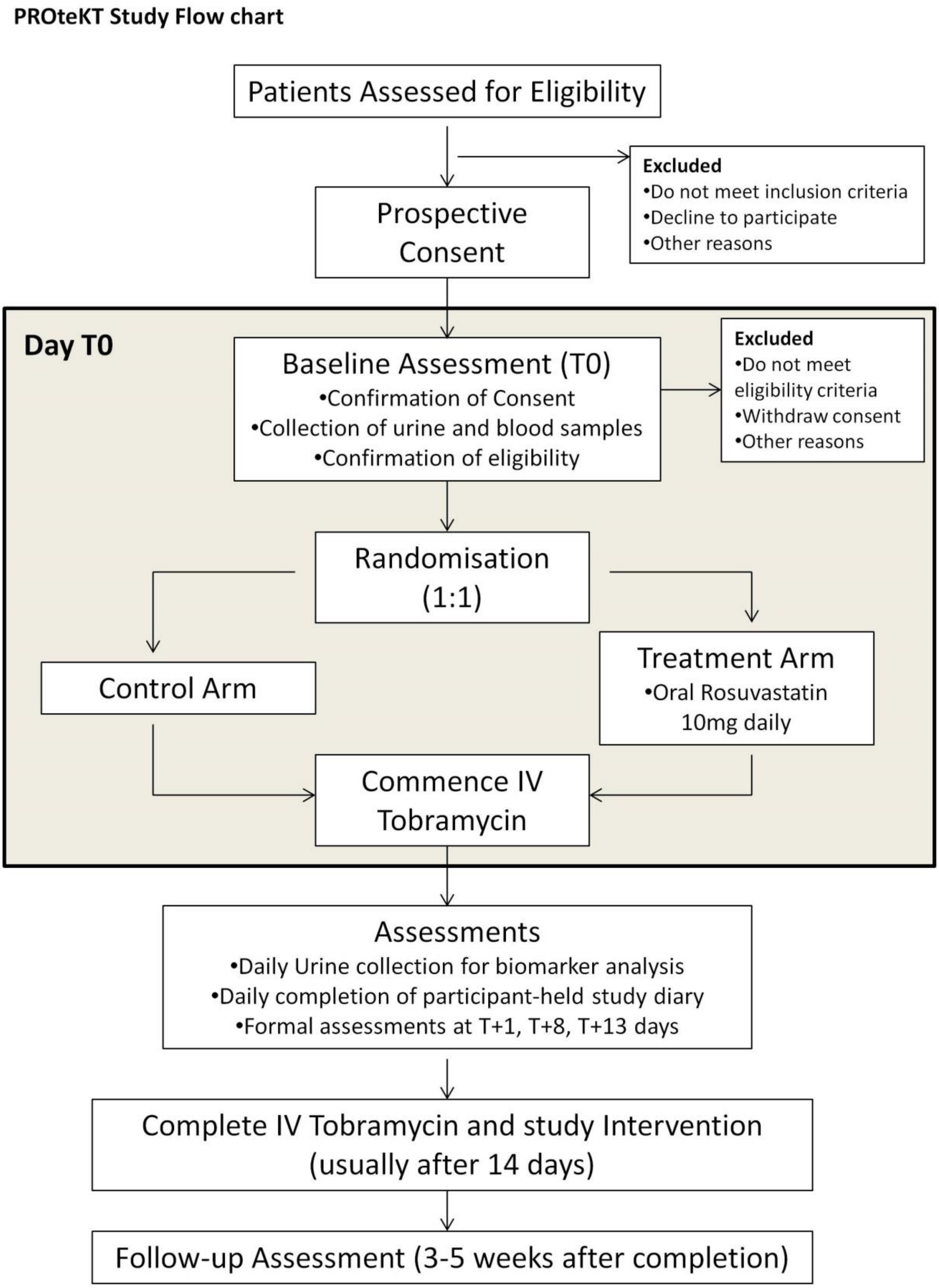

# 2 BACKGROUND INFORMATION

## 2.1 Background and Rationale

**Introduction:** Cystic Fibrosis (CF) is a common, inherited, life-limiting disease which affects around 9000 people in the UK alone.[1] 25% of children with CF aged 12-15 years have chronic pulmonary infection with *Pseudomonas aeruginosa*, and 40% by age 16-19 years.[1] There is improved survival with 2 week courses of intravenous (IV) antibiotics (an aminoglycoside plus a beta-lactam) up to 4 times per year. Aminoglycosides (AGs) are, however, potentially nephrotoxic. A UK national survey of Acute Renal Failure (ARF) in patients with CF found 24 cases between 1997 and 2004.[2] They estimated an incidence of ARF of 4.6 to 10.5 cases per 10000 CF patients per year. In a follow-on case-control study, they identified an 80-fold increase in the risk of ARF if CF patients received an AG within the preceding week.[3] Renal impairment (reduced creatinine clearance) is present in 31-42% of adult CF patients, and is associated with cumulative AG exposure (P=0.0055).[4, 5] Current strategies for the prevention of AG-induced nephrotoxicity include extended-interval dosing, and drug trough level monitoring with dose adjustment, but these are only partially effective. AGs are an important part of the management of children with CF, helping to improve survival. It is, however, important to develop further strategies to minimise their nephrotoxic consequences.

**Assessing aminoglycoside-induced nephrotoxicity:** AG-induced nephrotoxicity is characterized by selective targeting of the proximal tubule cells within the renal cortex. Accumulation of the drug within the proximal tubule epithelial cells, following glomerular filtration, is thought to be the key determining mechanism for the development of toxicity.[6] Endocytosis via the multi-ligand receptor, megalin, has been demonstrated to be the principal pathway for this accumulation.[7] Intracellular aminoglycoside can result in apoptosis and necrosis of proximal tubule epithelial cells by a variety of pathways (mitochondrial dysfunction and the release of reactive oxygen species).[8, 9]

Traditional measures of kidney function and of kidney injury rely on measurement of serum markers such as creatinine and blood urea nitrogen. These markers require invasive blood tests, and the information they provide is limited. Serum creatinine (sCr) is used currently to detect Acute Kidney Injury (AKI), but is of limited value since any change in its concentration is a delayed response with levels rising significantly above baseline levels only when 25-50% of renal function has been lost.[10] Furthermore, it is a marker of glomerular filtration, and therefore not specific to damage at other sites in the kidney nephron. Oliguria is, at best, a late sign of AKI, and may not be present in many forms of AKI especially those related to toxins.[11]

**Advantages of KIM-1:** There has been increasing interest recently in the development of novel urinary biomarkers, which not only are more sensitive than urea and creatinine, but also delineate damage to different parts of the nephron,[12, 13] and have the advantage of being non-invasive. Particularly promising is Kidney Injury Molecule-1 (KIM-1). KIM-1 is upregulated in proximal tubule epithelial cells in response to nephrotoxins, and excreted in the urine.[14, 15] Its upregulation parallels the degree of renal damage seen on histopathology in rodent studies.[16] Moreover, recent reports from a multi-site validation investigation in animal models treated

with a number of nephrotoxins, suggest that Kim-1 (KIM-1 in man) can outperform, with respect to sensitivity and specificity, a number of traditional and novel biomarkers of AKI.[17]

In clinical trials, KIM-1 has been shown to be an early diagnostic marker of acute kidney injury (AKI).[12] A systematic review found it to be one of the best performing biomarkers for the differential diagnosis of established AKI and for the prediction of mortality risk following AKI.[13] In our recent work, we measured various urinary biomarkers (KIM-1, Neutrophil Gelatinase-associated Lipocalin (NGAL), and N-acetyl-β-D-glucosaminidase (NAG)) in 41 neonates exposed to gentamicin for treatment of neonatal sepsis. We found a rise in KIM-1 levels during treatment with gentamicin, which remained significant even after adjusting for confounders.[18]

We have recently completed a study (URBAN CF) in healthy children to establish reference intervals for urinary KIM-1 and NGAL, as an important step in the validation of these two biomarkers for use in the paediatric population.[19] A longitudinal study in children with cystic fibrosis is ongoing. A preliminary analysis has demonstrated a significant correlation between baseline KIM-1 and the number of previous courses of aminoglycosides ($R=0.70$, $P<0.002$). During exposure to tobramycin, mean 'highest value' KIM-1 was significantly elevated from baseline (mean 'highest value' KIM-1, 1.24ng/mg Cr, 95% CI, 0.71-1.78ng/mg Cr, $p=0.02$, $n=10$). Mean fold change ('highest value' KIM-1 during tobramycin exposure:pre-treatment baseline KIM-1) was 3.03 (95% CI, 1.89-4.17).

**Preventing aminoglycoside-induced nephrotoxicity:** Given the problem of aminoglycoside induced nephrotoxicity, particularly in this vulnerable CF population, there is a need for better ways of prevention. Our work in the MRC Centre for Drug Safety Science (CDSS) in Liverpool has been focusing on this question, with a view to developing novel interventions that could be used in all age groups, with the ultimate aim of improving the benefit-risk ratio of aminoglycoside therapy. Given the mechanism of action of aminoglycosides in causing nephrotoxicity, we identified statins as a possible intervention. Statins are drugs widely used in cardiovascular disease in adults, with proven efficacy and safety. Statins are also used in children having been licensed principally for the treatment of hyperlipidaemia.

In order to test our hypothesis, we first used renal tubular cell models: using a number of statins, we were able to show that statins prevent aminoglycoside-induced cell toxicity *in vitro*[20] This effect occurs through the inhibition of HMG-CoA reductase by statins resulting in a depletion of the cellular sterols required for the megalin-mediated uptake of aminoglycosides into renal proximal tubular cells (Figure 1). The next step involved developing an animal model to identify which statin and which dose would be most appropriate in preventing aminoglycoside nephrotoxicity. It is important to note that not all statins are the same because they are handled differently by the body. Initial studies utilised a rat model: although we were able to show inhibition of aminoglycoside toxicity in this model, the doses of statins required were extremely high because of the high activity of HMG CoA reductase in the rat (Figure 2). We then progressed to a guinea pig model which does not have the same attributes in terms of HMG-CoA reductase activity as the rat. This was much more successful and was able to show inhibition of gentamicin-induced nephrotoxicity by rosuvastatin (Figure 3). However, the effect was not seen with simvastatin.

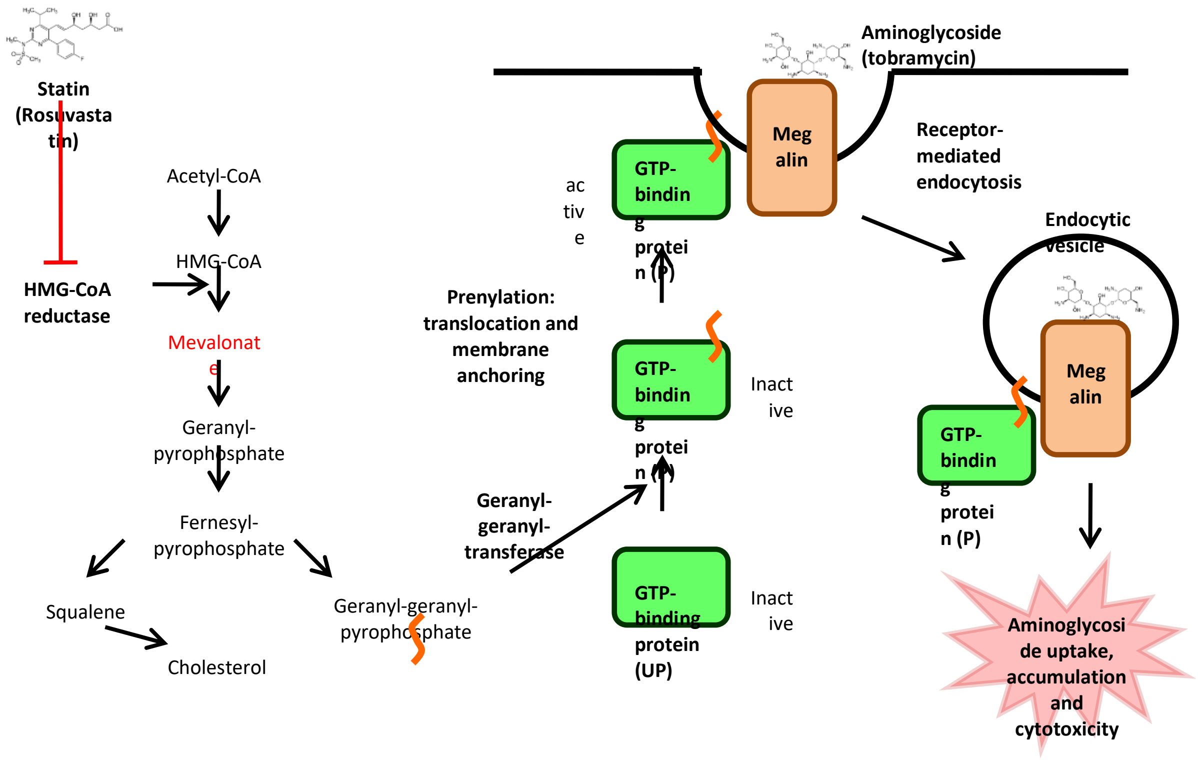


**Figure 1. Mechanism of inhibition of aminoglycoside nephrotoxicity by statins**

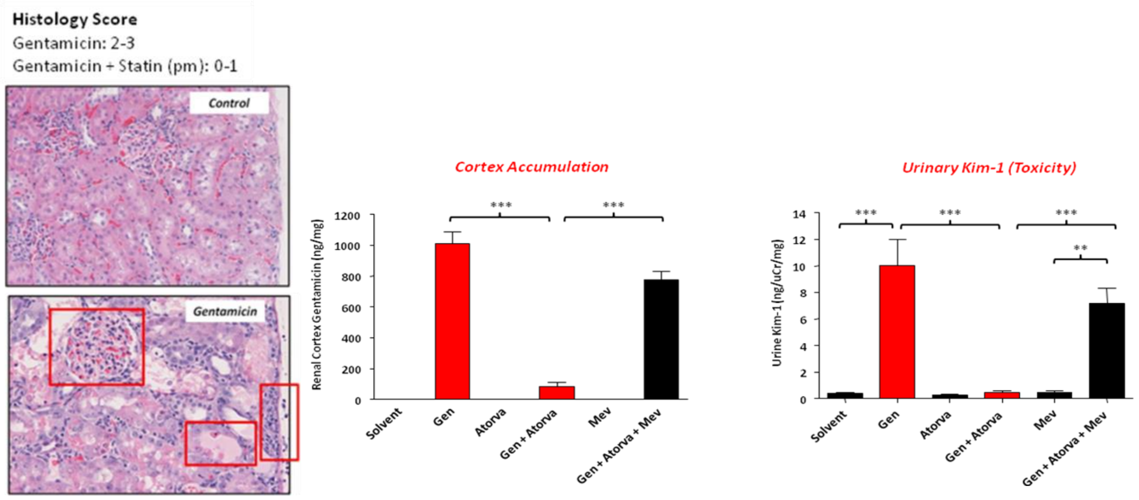


**Figure 2. Gentamicin-induced nephrotoxicity in a rat model, and the impact of atorvastatin.** Sprague Dawley (SD) Rats, (n=4 per group) were treated with solvent (control group), gentamicin (200mg/kg/day), Atorvastatin (40mg/kg/day), Gentamicin & Atorvastatin, Mevalonate (100mg/kg/day), and Gentamicin & Atorvastatin & mevalonate for 10 days. H&E figures: Gentamicin-induced renal effects in the SD rat. Histological determination reveals proximal tubule cell death by necrosis, luminal protein casts and inflammatory cell infiltration. The degree of proximal tubule cell loss is indicated on the figure and ranges in severity from 0-5. Bar charts: Atorvastatin prevents gentamicin accumulation into the renal cortex in SD rats and also the resulting toxicity (urinary Kim-1 – red bars). Co-treatment with mevalonate (the product normally inhibited by statin treatment), reverses the protective effect of atorvastatin *in vivo* (black bars).

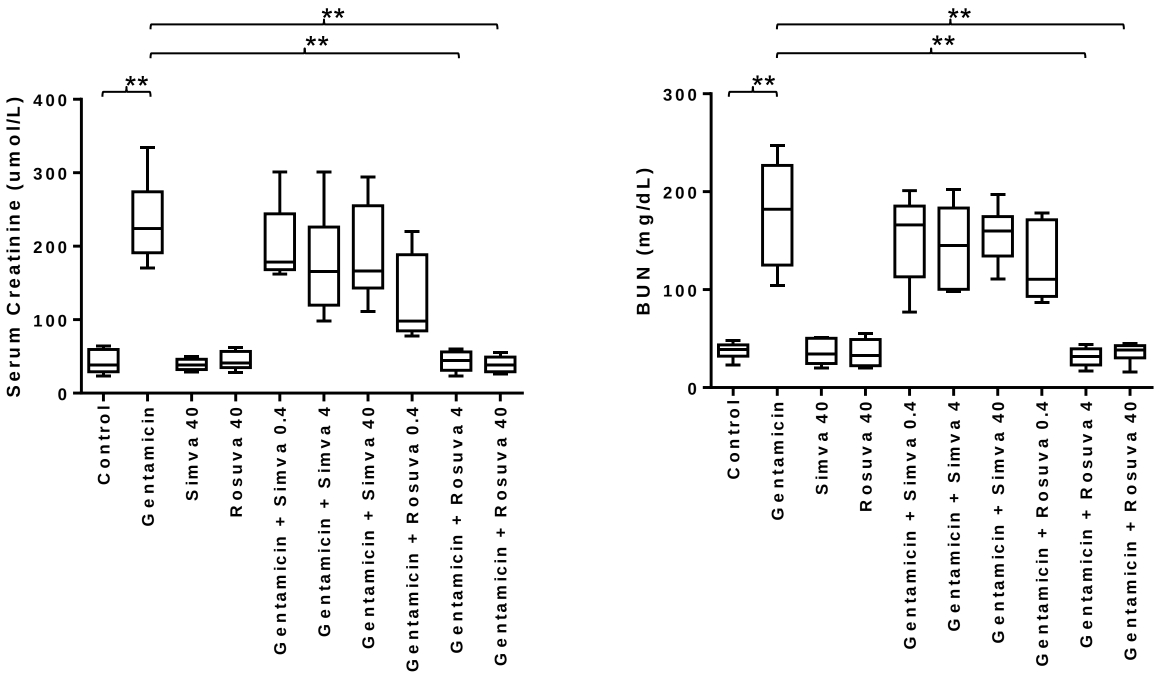


**Figure 3. Gentamicin-induced nephrotoxicity in a guinea pig model, and the impact of simvastatin and rosuvastatin.** Six guinea pigs per group were treated as indicated for 10 days. A Gentamicin dose of 100mg/kg/day was used. Simvastatin and Rosuvastatin were given at doses of 40, 4 or 0.4mg/kg/day as indicated.

A rosuvastatin dose-ranging study in the guinea pig model demonstrated inhibition of nephrotoxicity at a dose of 0.94mg/kg (Figure 4). Using a dose-scaling algorithm,[21] this equates to a rosuvastatin dose of 10mg in a 40kg child. We found that a dose of 0.47mg/kg in guinea pigs (equating to a 5mg dose in a 40kg child) did not inhibit nephrotoxicity. Our work is an example of translational medicine whereby work at the bench in a laboratory can be transferred to the clinical situation. It is also an example of repurposing of drugs – i.e. rosuvastatin may be useful in an indication which is different from that for which it was originally licensed. Rosuvastatin is already used in children for familial hypercholesterolaemia on a chronic basis, and no increased susceptibility of children to liver or muscle adverse effects has been identified, and there have been no effects on hormonal status and growth reported.[22]

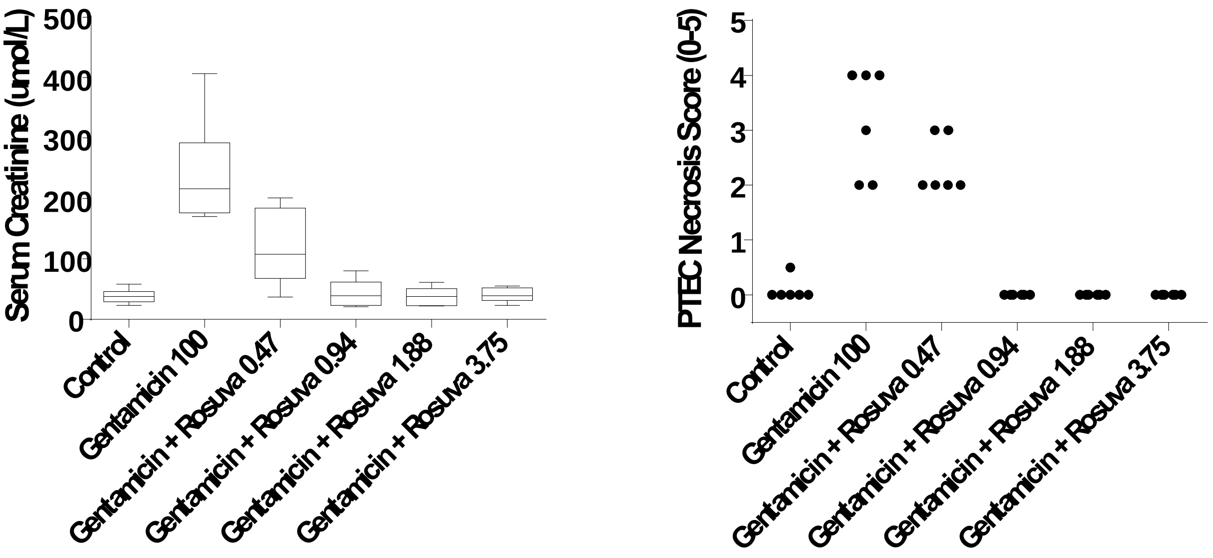


**Figure 4. Inhibition of Gentamicin-induced nephrotoxicity in a guinea pig model by Rosuvastatin: dose-ranging study.** Six guinea pigs per group were treated as indicated for 10 days. A Gentamicin dose of 100mg/kg/day was used. Rosuvastatin was given at doses of 3.75, 1.88, 0.94, or 0.47mg/kg/day.

**Need for a clinical intervention study:** The next stage of our translational journey is to test the hypothesis that rosuvastatin can inhibit aminoglycoside-induced nephrotoxicity in children with cystic fibrosis. We have therefore chosen to conduct this study using a 10mg dose of rosuvastatin for reasons outlined above. It will use change in urinary KIM-1 during aminoglycoside exposure as its primary outcome measure. This phase IIa study, if positive, will be used to design a national, multi-centre, phase IIb/III study to evaluate the effect of rosuvastatin in preventing aminoglycoside-induced kidney injury.

The development of an intervention strategy to minimise the nephrotoxic consequences of aminoglycosides would enable patients to continue to benefit from their positive impacts, but reduce their risk of suffering acute and long-term adverse consequences.

## 2.2 Objectives

**Primary Objective:** This trial will evaluate the effect of rosuvastatin on aminoglycoside-induced nephrotoxicity. This will be assessed using the difference in mean fold-change in urinary KIM-1 from baseline to ‘highest value’ concentration during exposure to tobramycin between the rosuvastatin treated arm and control arm.

**Secondary Objectives:**

1. Change in serum concentration of creatinine and eGFR during tobramycin exposure between rosuvastatin treated arm and the control arm.
2. Change in other urinary and plasma biomarkers of renal injury during tobramycin exposure between rosuvastatin treated arm and the control arm.
3. Difference in serious adverse events between rosuvastatin treated arm and the control arm.
4. Difference in tobramycin concentrations between rosuvastatin treated arm and the control arm to identify any pharmacokinetic interaction between rosuvastatin and the tobramycin.

5. Difference in Forced Expiratory Volume in 1 second (FEV1) and C-Reactive Protein, between rosuvastatin treated arm and the control arm to identify any pharmacodynamics interaction between rosuvastatin and the tobramycin
6. Assessment of plasma rosuvastatin concentrations achieved in children randomised to the intervention arm.
7. Difference in biomarkers of *Pseudomonas aeruginosa* between rosuvastatin treated arm and the control arm.
8. Assess the feasibility of collecting DNA for a molecular genetic study of aminoglycoside-induced nephrotoxicity.

## 2.3 Potential Risks and Benefits

Rosuvastatin has a marketing authorisation for use in children for the management of hypercholesterolaemia. It can be used at doses up to 20mg/day in children aged 10 to 17 years, and up to 10mg/day in children aged 6 to 9 years. Our trial represents an example of drug repurposing, whereby we will use rosuvastatin outside its licence to prevent an important adverse effect. Guidance issued by the MRC, Department of Health and the MHRA on risk-adapted approaches to the management of CTIMPs propose a three level categorisation for the potential risk associated with the IMP, assigned according to the following categories:

***Type A*** *'no higher than that of standard medical care';*
***Type B*** *'somewhat higher than that of standard medical care';*
***Type C*** *'markedly higher than that of standard medical care'.*

Whilst rosuvastatin has a marketing authorisation, in this study it will be used outside the manufacturer's indication, and therefore the risk associated with the IMP in this trial is categorised as ***Type B 'somewhat higher than that of standard medical care'.*** This level of risk informs the risk assessment, regulatory requirements, nature and extent of the monitoring, and the management processes used in the trial.

Participants will be closely monitored for known potential adverse events, and for unexpected adverse events. The management of any symptoms or exacerbations will be in accordance with usual clinical practice and either the local principal investigator (PI) or delegated research staff, will be available throughout the study to discuss specific issues with individuals concerned. Any concerns, which cannot be satisfied at a local level, will be forwarded to the chief investigator (CI). Any participant can withdraw from the study at any time without detriment to their future care. All ethical aspects of the study will be discussed when informed written consent is obtained. Appropriate information leaflets have been developed and will be discussed at the screening consultation. Potential participants will be provided with a copy of the information sheets and their signed consent forms.

### 2.3.1 Potential Risks

A previous trial has been conducted using simvastatin at a dose of 40mg in children with CF for the reduction of airway inflammation (NCT00255242). However, although the trial is recorded as completed, the outcomes have not yet been published. Participation in this trial does not change existing CF treatment protocols. We will also be using rosuvastatin for short periods rather than chronic use which characterises hyperlipidemia.

Short-term rosuvastatin treatment in children who have normal lipid profiles may potentially result in transient hypolipidaemia. We are not aware of any evidence of adverse effects related to short-term hypolipidaemia, but this will be routinely monitored in all patients during the trial.

Rosuvastatin can have rare but serious adverse effects involving skeletal muscle and the liver. We will also monitor for these by measuring creatine kinase and liver function tests, and also by monitoring for signs or symptoms daily whilst on rosuvastatin. We will exclude patients taking medications known to interact with rosuvastatin, or with conditions or past history that may lead to an increased susceptibility to adverse effects of rosuvastatin.

One practical issue is the addition to the already existing pill burden in this group of patients; however, rosuvastatin is administered as a single oral tablet taken only once daily, and thus, is likely to cause a minimal increase in the pill burden. There is no further additional burden to participants in the trial.

### 2.3.2 Known Potential Benefits

There are potential benefits if our hypothesis that rosuvastatin can prevent aminoglycoside-induced kidney injury is proven.

# 3 SELECTION OF CENTRES/CLINICIANS

Potential study centres will be identified through the Clinical Research Network: Children (CRN: Children). Site set-up will also be facilitated by the CRN. Study centres will be initiated once all global (e.g. local R&D approval) and study-specific conditions (e.g. training requirements) have been met, and all necessary documents completed. Site Initiation visits will take place.

## 3.1 Centre/Clinician Inclusion Criteria

Each participating centre (and Principal Investigator; PI) has been identified on the basis of:

- Being a paediatric cystic fibrosis (CF) treatment centre
- Having at least one lead clinician with a specific interest in, and responsibility for supervision and management of paediatric patients with CF
- Showing enthusiasm to participate in the study
- Ensuring that sufficient time, staff and adequate facilities are available for the trial
- Planning to provide information to all supporting staff members involved with the trial or with other elements of patient management
- Feasibility data confirms that they have a number of eligible patients
- Acknowledging and agreeing to conform to the administrative and ethical requirements and responsibility of the study including adhering to GCP and other regulatory documentation
- Other important criteria are:
    a. Local R&D approval
    b. Completion and return of 'Signature and Delegation Log'
    c. Signed non-commercial agreement between centre and sponsor
    d. Receipt of evidence of completion of (a),(b) and (c)

## 3.2 Centre/Clinician Exclusion Criteria

a. Not meeting the inclusion criteria listed above

# 4 TRIAL DESIGN

This study is a phase IIa, multi-centre, randomised, open-labelled trial of rosuvastatin in children with cystic fibrosis (CF) receiving clinically indicated treatment with intravenous (IV) tobramycin.

Patients will be randomised equally to either receive rosuvastatin 10mg once daily or no intervention (control), throughout a course of treatment with IV tobramycin (usually lasting 14 days).

If at the final analysis, a significant reduction in fold-change of KIM-1 during tobramycin exposure is found, rosuvastatin 10mg will be recommended for phase IIb/III.

In a previous study (URBAN CF) urine biomarkers have been measured in children with CF receiving treatment with tobramycin. Preliminary results, specifically the fold-change in urinary KIM-1 from baseline to ‘highest value’ concentration during exposure to tobramycin, from this study have been used to inform the design of the present study. According to this power calculation, a sample size of 20 in each arm will be recruited (See section 9.4).

## 4.1 IV Tobramycin

This study will include only children with CF treated with the aminoglycoside antibiotic tobramycin given intravenously. We will not include those children receiving treatment with other aminoglycoside antibiotics or receiving nebulised aminoglycoside therapy. IV tobramycin is usually given once daily, but can also be given three times per day. Participants may receive tobramycin at either frequency (as decided by the local CF team on clinical grounds), but this should be specified in the study CRFs, and the time and amount of each dose should be accurately recorded.

## 4.2 Primary Outcome

This trial will evaluate the effect of rosuvastatin on aminoglycoside-induced nephrotoxicity. This will be assessed using the difference in mean fold-change in urinary KIM-1 from baseline to ‘highest value’ concentration during exposure to tobramycin between the rosuvastatin treated arm and control arm. Urinary KIM-1 will be measured using a validated assay in a GCLP Laboratory at the University of Liverpool. Urinary KIM-1 will be normalised to urinary creatinine.

## 4.3 Secondary Outcome(s)

1. Difference in serum concentration of creatinine and eGFR during tobramycin exposure between rosuvastatin treated arm and the control arm.
2. Difference in other urinary and plasma biomarkers of renal injury during tobramycin exposure between rosuvastatin treated arm and the control arm.
3. Difference in serious adverse events between rosuvastatin treated arm and the control arm.
4. Difference in tobramycin concentrations between rosuvastatin treated arm and the control arm to identify any pharmacokinetic interaction between rosuvastatin and the tobramycin.

5. Difference in Forced Expiratory Volume in 1 second (FEV1) and C-Reactive Protein, between rosuvastatin treated arm and the control arm to identify any pharmacodynamics interaction between rosuvastatin and the tobramycin
6. Relationship between plasma rosuvastatin concentrations achieved in children randomised to the intervention arm and change in urinary KIM-1.
7. Difference in biomarkers of *Pseudomonas aeruginosa* between rosuvastatin treated arm and the control arm.

### 4.3.1 Description of Secondary Outcomes

- Change in traditional markers of renal injury, serum creatinine and estimated Glomerular Filtration Rate, and change in novel urinary and plasma biomarkers of renal injury during tobramycin exposure between the rosuvastatin treated arm and the control arm. Serum creatinine will be measured locally, and novel biomarker analysis (NGAL, cystatin C) will occur centrally at the University of Liverpool.
- To assess safety of the intervention, we will compare the reported expected and unexpected serious adverse events between the rosuvastatin treated arm and the control arm. We will particularly focus on muscle adverse events which have been reported with rosuvastatin. CPK will be measured in these patients through blood that is routinely collected.
- We will assess for interaction between rosuvastatin and tobramycin:
    - Pharmacokinetically, by comparing tobramycin concentrations between the rosuvastatin treated arm and the control arm. Blood samples will be taken at 3 time-points during tobramycin exposure and analysed locally for the tobramycin concentration. Any additional blood samples taken during the study period will also be analysed for tobramycin concentration to give additional data.
    - Pharmacodynamically, by comparing change in percent of predicted Forced Expiratory Volume in 1 second (FEV1), between the rosuvastatin treated and control arms. This is a widely used indirect measure of aminoglycoside treatment outcome in children with CF, and will be measured locally during study visits. We will also compare change in CRP, a widely used marker of inflammation/infection, between the two groups.
- In the rosuvastatin treated arm, collected blood samples will be analysed centrally for rosuvastatin concentration, in order to assess the pharmacokinetic profile of rosuvastatin in children with CF, to assess compliance, and to relate rosuvastatin concentrations to change in urinary KIM-1.
- We will assess the impact on *Pseudomonas aeruginosa* by measuring biomarkers associated with *P. aeruginosa* quorum sensing which can be used to determine both the presence of *P. aeruginosa* as well as markers of virulence. These markers will be compared between the rosuvastatin treated and control arms

## 4.4 PROteKT substudy - Molecular genetics of aminoglycoside-induced nephrotoxicity

All children who are invited to participate in the PROteKT study will also be invited to participate in a substudy to assess the molecular genetics of aminoglycoside-induced nephrotoxicity.

There exists a considerable degree of inter-individual variability in susceptibility to aminoglycoside-induced nephrotoxicity, and the reasons for this are not clear from the

available literature. In particular, whether there is any genetic component to this variability has not been investigated previously in a genome-wide approach.

During this study all participants will be exposed to IV tobramycin as part of their routine clinical care. We will collect a sample for molecular genetic analysis from each child who consents to do so, and identify aminoglycoside-induced nephrotoxicity using novel biomarkers and serum creatinine.

The samples collected in this study will be combined with:

1. Samples collected through the MAGIC study (Molecular Genetics of Adverse Drug Reactions in Paediatric Patients), Research Ethics No: 10/H1002/57.
2. Samples collected through worldwide efforts to evaluate genetic factors predisposing to drug-induced renal injury.

In the future, we will conduct a genome wide association study (GWAS) to identify genetic risk factors for aminoglycoside-induced nephrotoxicity. Any positive hits will be further investigated using functional approaches to elucidate mechanisms and identify causal variants.

We will aim to assess the feasibility of carrying out a molecular genetic study in this group of children, and to address the following objectives:

(1) Identify genetic risk factors for aminoglycoside-induced nephrotoxicity in children in order to provide better preventive strategies in the future.

(2) Identify causal mechanisms of aminoglycoside-induced AKI by exploring genotype/phenotype correlations, in order to provide better interventional strategies in future.

# 5 STUDY POPULATION

The study will recruit children with cystic fibrosis from paediatric CF treatment centres in the UK.

## 5.1 Inclusion Criteria

1. Age 6 to 18 years inclusive.
2. Diagnosis of cystic fibrosis (established by sweat test or genotype).
3. Planned, clinically indicated, course of treatment with IV tobramycin.
4. Ability to give informed consent.
5. Willingness to comply with all study requirements.
6. Able to take tablets.

## 5.2 Exclusion Criteria

1. Existing treatment with a statin.
2. Previous adverse reaction to a statin.
3. Co-enrolment in other drug trials*, or completion of a previous CTIMP within the last 30 days.
4. Previous randomisation in the PROteKT trial.
5. Patients taking any of the following medications: Ciclosporin, Protease Inhibitors, Fibrates, Ezetimibe, Erythromycin (but not other macrolides), Eltrombopag, Dronedarone, Coumarins, Oral contraceptives, nicotinic acid, fusidic acid and simepravir.
6. Female participants who are pregnant or lactating or refuse a pregnancy test if of childbearing potential (female participants of childbearing potential must use a barrier method of contraception if sexually active whilst taking rosuvastatin and for 7 days afterwards).
7. Patients of Asian ancestry (Japanese, Chinese, Filipino, Vietnamese andKorean).†
8. Patients with renal disease (eGFR<60ml/min/1.73m$^2$, using the Schwartz formula[23]**, in the 6 months preceding screening).
9. Patients with current elevation in transaminases exceeding 3x the upper limit of normal, in the past 12 weeks.
10. Patients with current elevation in creatine kinase exceeding 2x the upper limit of normal at baseline, or in the past 12 weeks ***.
11. Family history, or personal history, of hereditary muscular disorders.
12. Patients with myopathy.
13. Patients with a history of, or active alcohol abuse.
14. Patients with hypothyroidism.
15. Patients with galactose intolerance, the Lapp lactase deficiency, or glucose-galactose malabsorption.
16. Patients who are Hepatitis C positive or HIV-positive.

***Patients who are currently taking part in TORPEDO-CF are allowed take part in PROteKT as long as their date of randomisation into TORPEDO-CF is not within the previous six months of the screening date for PROteKT.**

****Schwartz formula to calculate eGFR = [40 * height (cm)] / serum or plasma creatinine (µmol/l)**

*** If creatine kinase in the past 12 weeks exceeds 2x the upper limit of normal this should be confirmed with the baseline bloods. If the baseline creatine kinase exceeds 2x the upper limit of normal this test should be repeated within 6 hours. If the creatine kinase remains elevated above 2x the upper limit of normal then the patient should be excluded. If the repeat creatine kinase is less than 2x the upper limit of normal, the patient may be included.**

**† Patients of Asian-Indian ethnicity may be included as a recent pharmacokinetic study[23] suggests that patients of Asian-Indian ethnicity would experience only about a 30% increase in exposure to rosuvastatin which is unlikely to be clinically significant.**

Exclusion criteria are based upon those contraindications or cautions outlined in the Summary of Product Characteristics.

## 5.3 Patient Transfer and Withdrawal

In consenting to the trial, patients are consented to trial treatment, follow-up and data collection. If voluntary withdrawal occurs, the patient should be asked to allow continuation of scheduled evaluations, complete an end-of-study evaluation if appropriate and be given appropriate care under medical supervision until the symptoms of any adverse event resolve or the patient's condition becomes stable.

Follow-up of these patients will be continued through the trial research nurses (RN) and the lead investigator at each centre unless the participant explicitly also withdraws consent for follow-up.

### 5.3.1 Patient Transfers

For patients moving from the area, every effort should be made for the patient to be followed-up at another participating trial centre if possible and for this trial centre to take over responsibility for the patient. A copy of the patient CRFs should be provided to the new site. The patient will have to sign a new consent form at the new site, and until this occurs, the patient remains the responsibility of the original centre. The study co-ordinator should be notified in writing of patient transfers.

### 5.3.2 Withdrawal from Trial Intervention

Patients may be withdrawn from the trial treatment rosuvastatin for any of the following reasons:

a. Patient withdraws consent;
b. Clinical decision not to treat with tobramycin;
c. Unacceptable adverse effect of Grade 3/4 which can be attributed to rosuvastatin;
d. Intercurrent illness preventing further treatment;
e. Inability to attend regularly for treatment or assessment;
f. Any change in the patient's condition that justifies the discontinuation of treatment in the clinician's opinion;
g. Failure to comply with the protocol requirements or cooperate with the investigator;
h. The patient starts treatment with any disallowed medication without prior notification and the consent of the investigators;
i. Pregnancy during trial treatment period;
j. If the inclusion/exclusion criteria change following consent.

If a patient wishes to withdraw from trial treatment, centres should nevertheless explain the importance of remaining on trial follow-up, or failing this, of allowing routine follow-up data to be used for trial purposes. Generally, follow-up will continue unless the patient explicitly also withdraws consent for follow-up (see Section 5.3.3). If tobramycin is stopped before the 2 week period, rosuvastatin must also be stopped (see section 7.3.5). If a clinical decision is taken to withdraw a patient from the trial, a reason for this must be provided on the Withdrawal CRF form.

Patients who withdraw from trial treatment but are willing to allow further data collection must have a discussion with the investigator as to whether they will be able to attend subsequent follow-up assessments at specific time-points until the end of their follow-up period. The decision should be based on the patient’s own preferences and the clinician.

### 5.3.3 Withdrawal from follow-up

Every effort should be made to collect follow-up data even if participants have withdrawn from trial intervention. In some cases it may not be possible to continue follow-up of trial participants due to transfer to a non-participating centre, loss to follow-up etc. in which case the withdrawal form should be completed. Where a patient is considered as lost to follow-up or if the patient has stated that they do not wish to continue follow-up, the data collected up until that point will be used in the analysis unless the patient explicitly states that they require all data to be removed (see 5.3.4).

If patient withdraws consent for blood samples, then participation with just urine tests can continue until end of follow up as long as the patient consents to this.

### 5.3.4 Withdrawal of consent for participation

Patients are free to withdraw consent at any time without providing a reason. If a patient withdraws consent for treatment this should not be considered as withdrawal of consent for follow-up unless the patient explicitly states that they no longer wish to participate in follow-up. Where patients wish to withdraw consent for the trial the data collected up to the time of withdrawal of consent will be included in the analyses unless the patient explicitly states that they require all data collected to be removed. The study co-ordinator should be informed in writing and a withdrawal CRF should be completed in order for the CTRC to ensure that data are removed and not made available for analyses. The patient will not contribute further data to the study.

# 6 ENROLMENT AND RANDOMISATION

## 6.1 Recruitment

Patients who are eligible for inclusion into the trial will be identified and recruited through the paediatric cystic fibrosis centres participating in the study.

Potentially eligible participants will be identified by their clinical care team at each centre via a search of the patient database/s either electronically or manually or by clinic list or clinical record review. A short introductory letter about the study, along with a parent information sheet and age-appropriate information sheet for the child, will be sent out to the family at least 24 hours prior to their clinic visit. The introductory letter will ask them to read the information sheets, and will explain that they will be approached by the research team when they come to the clinic to consider consenting to participate in the study.

In the CF outpatient clinic children (and their parents/guardian) who are eligible will be approached by a member of the healthcare team who is also a member of the research team. If they have not had opportunity before coming to the clinic, parents and children will be given time to read the study information sheets. The study will then be verbally presented, and any questions arising from this or the patient information sheets will be answered. If they are willing to participate, informed, written consent will be obtained (see Section 11.3 for the consent procedure) from the parents, or the participant if aged 16 years or over. An assent form will be completed by the child if aged under 16 years. This consent will be considered ‘prospective’ and will be re-confirmed when the child attends for their baseline assessment. ‘Prospective’ consent should be completed as close to randomisation as possible. Eligibility blood tests can be done at this visit if this is more convenient than being done during the baseline assessment.

If potential participants feel in need of more time to consider their response, they will be approached again at their next outpatient appointment.

A situation may arise where a potential participant is identified in clinic, but has not previously received the study information in the post. In this instance the patient should be given the study information during their outpatient appointment and may then and may be approached for consent at their next outpatient appointment. However, if they are due to commence treatment with tobramycin before their next outpatient appointment, then they may be approached for consent on the day of attendance to commence their IV antibiotics. Consent may be obtained within 24 hours but only on the rare occasion when a potential participant is due to commence treatment with tobramycin within 24 hours of attending their outpatient appointment, and where they have not previously been sent the study information.

However, this should only occur if the patient fully understands the risks and benefits of taking part and has fully read and understood the patient information sheet and is satisfied that all their questions have been answered by a member of the research team. Only then will the patient be asked to provide consent. It should be made clear to patients that (1) they do not have to take part, (2) they have every right to require more time to consider participation and (3) the care they receive will not be affected should they not want to take part.

### 6.1.1 PROteKT substudy - Molecular genetics of aminoglycoside-induced nephrotoxicity

Information about the substudy will be included in a separate information sheet to the main PROteKT sheet, and will be sent to eligible participants at the same time as the main information sheet. They will be given the same time as the main trial to consider their participation in the substudy and will be asked to consent at the time of consenting to the main PROteKT study.

## 6.2 Screening

Children with CF aged 6 to 18 years who are considered likely to require treatment with IV tobramycin during the study period should be approached about the study. A 'Screening Log' will be maintained of all the patients who undergo screening regardless of whether they decide to participate in the study or are found ineligible to participate. Reasons for not being eligible will be recorded. Reasons for declining to participate will be asked routinely but it will be made clear that they do not have to provide a reason unless happy to do so.

## 6.3 Baseline (T0)

Baseline assessment may be completed on the same day that consent is provided or on another day (as long as this is within one week before the patient commences their IV Tobramycin treatment). Alternatively, on the planned day for beginning tobramycin therapy, participants will be asked to attend early in the day for baseline assessment. The research team should conduct the baseline (T0) assessments and complete the eligibility and baseline case report form (CRF) during the baseline assessment. The baseline assessments include:

1. Verification that the eligibility criteria are fulfilled;
2. Confirmation of consent;
3. Demographic details including age, gender, ethnicity;
4. Full medical and drug history (including concomitant medications such as contraceptives). This will also include an assessment of symptoms normally associated with rosuvastatin toxicity such as muscle pains in order to get an indication of the prevalence of these symptoms prior to statin exposure;
5. Physical examination;
6. Body weight and height;
7. Pregnancy test for females of childbearing potential (urine): if a potential participant refuses the test, they should not be included in the study;
8. Measurement of FEV1;
9. Collection of a Sputum sample;
10. Collection of blood samples, as necessary (in particular those not done at consent), from each patient;
11. Collection of one urine sample from each patient at the same time as the blood samples.

## 6.4 Randomisation

Randomisation should ideally occur once the results of all eligibility criteria have been met. However, it may be necessary to randomise prior to completion of these in some circumstances (for instance, where the participant is being admitted over the weekend and facilities do not exist for the IMP to be dispensed at the weekend). Participants can be randomised equally to either receive rosuvastatin 10mg once daily or no intervention (control) via two randomisation pathways (Figure **):

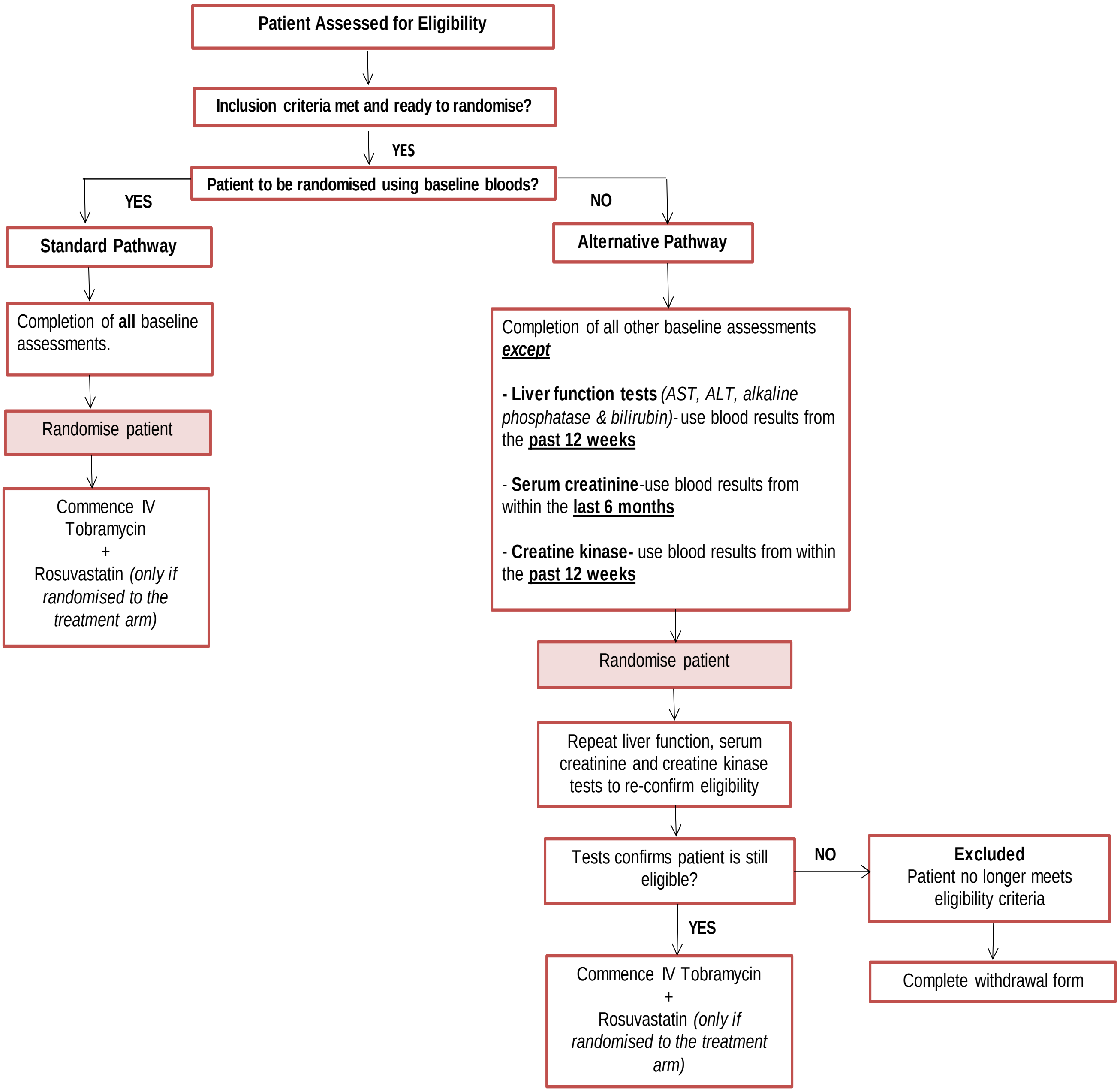


Figure **: Randomisation Pathways

If baseline assessment blood results are received following randomisation then it will be necessary to ensure that the participant still meets all the eligibility criteria (including pregnancy test). Treatment should only commence once all blood results from baseline assessment are available and eligibility is confirmed. If a participant no longer meets the criteria they will have to be withdrawn, and the reason for this documented appropriately.

***Elevated creatinine kinase at baseline***
In the event of a patient's creatinine kinase level being greater than 2 times the upper limit of normal at baseline or in the past 12 weeks the creatinine kinase levels should be repeated within 6 hours. Ideally randomisation should occur only once the result of the second creatinine kinase level is available, and only if it is less than or equal to 2 times the upper limit of normal. If necessary, the patient can be randomised whilst awaiting the repeat creatinine kinase level, however the patient can only commence treatment if the creatinine kinase level is less than or equal to 2 times the upper limit of normal after the repeat test. If the repeat is >2 times the upper limit of normal the patient would then have to be excluded from the study.

Appropriately delegated research staff at centres will randomise participants using a secure (24-hour) web based randomisation programme controlled centrally by the CTRC. Research staff will be trained to use the randomisation systems as part of the greenlight process for opening centres. Once the site has been initiated, they will be issued with usernames and passwords; new staff can be issued with usernames and passwords if they are delegated with the responsibility for randomising participants.

The randomisation system will randomise equally between the two arms using variable block randomisation. Participant treatment allocation will be displayed on a secure webpage and an automated email confirmation sent to the authorised randomiser, the PI and the trial manager (TM). The email should be printed out and filed alongside the participant's CRF as this provides information about the randomised treatment, randomisation number and date randomised.

It is the responsibility of the PI or delegated research staff to inform the pharmacy department at their centre prior to randomisation to ensure there is enough supply of the study drugs.

| |
|---|
| **Randomisation: web access** https://ctrc.liv.ac.uk/Randomisation/Protekt<br><br>*If there are any problems with web randomisation, please contact*<br>the trial manager using the following email address: protekt_trial@ucl.ac.uk<br><br>*Randomisation backup envelopes will be used in case of failure of the randomisation systems outside working hours* |

In the event of problems with the online randomisation system, the centre should contact the Trial manager at UCL (Monday to Friday between 9:00 to 17:00 excluding bank holidays) to try to resolve the problem.

### 6.4.1 Back-up randomisation

The web based randomisation system should always be used for randomising participants into the study. If there is a system failure that occurs outside UCL/CTRC office hours or when a system failure cannot be resolved in a reasonable time-frame by UCL/CTRC a randomisation envelope can be used. All centres will be provided with emergency back-up randomisation envelopes for this purpose.

In the event that emergency back-up envelopes are required, the randomising person will select the next sequentially numbered, opaque, pressure-sealed envelope that will give the randomisation allocation. The envelope will be similar to those used for pay slips, which cannot be viewed without fully opening and their construction is resistant to accidental damage or tampering.

As the envelope contains all the details of the randomisation, each page of the open envelope should be scanned and emailed to the trial manager and a photocopy can be sent to CTRC along with completed CRFs. Please ensure that the envelope is stored in the patient's medical notes.

The RN will check to ensure that the correct number of randomisation envelopes is present, that they are intact and that the sequential numbering system is maintained. Any discrepancies should be immediately reported to the trial manager.

## 6.5 Subsequent courses of aminoglycoside

Participants will not be given the option to consent to be re-randomised on subsequent occasions of treatment with IV tobramycin (exclusion criteria 4).

# 7 TRIAL TREATMENT

## 7.1 Introduction

Rosuvastatin (marketed as Crestor®; AstraZeneca UK Ltd) is indicated for clinical use in the treatment of hypercholesterolaemia. However, the current trial will use rosuvastatin outside the manufacturer's indication.

Participants recruited into the study will be randomised to one of the following arms:

**Control Arm:** Non intervention arm

**Treatment Arm:** oral rosuvastatin 10 milligram (mg) dose, once daily, for the duration of a treatment course of IV tobramycin (usually 14 days)

## 7.2 Formulation, Packaging, Labelling, Storage and Stability

The rosuvastatin used in the trial will be sourced via usual local NHS procurement arrangements once the sites have been initiated. The size of the procurement of investigational drug at each site will be pre-determined based on the patient recruitment target for that individual site. Recruitment will be monitored centrally and drug procurement will be tailored in liaison with the respective pharmacies to ensure that pharmacies always hold adequate supplies of trial treatment.

### 7.2.1 Description and Composition of the Drug Product

**Rosuvastatin:**
Generic name – Rosuvastatin (Brand name: Crestor®).

Rosuvastatin is a selective and competitive inhibitor of HMG-CoA reductase, the rate-limiting enzyme that converts 3-hydroxy-3-methylglutaryl coenzyme A to mevalonate, a precursor for cholesterol. It is used to treat hypercholesterolaemia, and to prevent cardiovascular events in those who are at risk.

Rosuvastatin tablets are for once-daily treatment, and may be given at any time of day, with or without food.

Crestor® 10 mg oral tablets: Each tablet contains 10 mg rosuvastatin (as rosuvastatin calcium). Each tablet contains 91.3 mg lactose monohydrate. It is a Round, pink coloured, Film-coated tablet intagliated with 'ZD4522' and '10' on one side and plain on the reverse.

Crestor® 10 mg oral tablets contain the following excipients: Tablet core; Lactose monohydrate, Microcrystalline cellulose, Calcium phosphate, Crospovidone, Magnesium stearate; Tablet coat; Lactose monohydrate, Hypromellose , Triacetin, Titanium dioxide (E171), Ferric oxide, red (E172).

Crestor® is manufactured by AstraZeneca UK Ltd.

### 7.2.2 Packaging and Labelling

Crestor® 10 mg tablets are packaged in blisters of aluminium laminate/aluminium foil of 7, 14, 15, 20, 28, 30, 42, 50, 56, 60, 84, 90, 98 and 100 tablets (although not all packages may be marketed).

Blister packs will be labelled and dispensed for trial treatment locally at each site in accordance with Annex 13 of Good Manufacturing Practice.

### 7.2.3 Stability and Shelf life

Rosuvastatin should be stored as per the Summary of Product Characteristics (SPC). Please refer to the reference SPCs provided as a separate document to this protocol.

The product should be stored below 30ºC in the original package to protect the tablets from moisture. The 10 mg tablet has a shelf life of 3 years. The IMP will be stored in local pharmacy departments in line with the conditions of the SPC. No special requirements above normal pharmacy practice for temperature monitoring or other processes have been identified.

## 7.3 Preparation, Dosage and Administration of Study Treatment/s

### 7.3.1 Dispensing

For each randomised patient who receives the intervention arm (Rosuvastatin), treatment will continue for the duration of their treatment course of IV tobramycin (usually 14 days).

The trial treatment can start immediately after randomisation and must be given on the same calendar day and prior to the first dose of IV tobramycin. The PI or delegated other will issue a prescription based on the patient's randomisation status. For participants in the treatment arm, rosuvastatin will be dispensed at a dose of 10mg once daily, for the duration of their treatment course of IV tobramycin, unless interruption or discontinuation is warranted (see sections 5.3.2 and 7.3.4).

For participants receiving the trial treatment as an inpatient, the prescription will be written on an inpatient prescription chart. For participants receiving the trial treatment at home, the prescription will be written on an outpatient prescription chart.

The respective pharmacy will dispense the trial treatments labelled as described in section 7.2.2.

The medications will be dispensed upon production of a valid, signed trial prescription to either the RN or directly to the patient as detailed below:

#### 7.3.1.1 Dispensing for the Control Arm:

For patients randomised to the Control Arm (the non-intervention arm), no prescription will be given.

#### 7.3.1.2 Dispensing for the Treatment Arm: Oral rosuvastatin 10 mg, once daily

For those who are randomised to the treatment arm, sufficient trial treatment (usually 14 tablets) consisting of blister packs of rosuvastatin 10mg will be dispensed on receipt of a valid trial prescription.

### 7.3.2 Lost or Damaged Medications

In the event of loss or damage to the tablet pack they are currently using, the participant (or their parent/guardian) or the inpatient ward should contact the RN who will arrange that the patient can be dispensed a replacement prescription in sufficient time to ensure there is no break in medication.

### 7.3.3 Administration

The patient will be instructed in the correct use of the medications dispensed. Further guidance will be provided throughout the remainder of the trial where necessary.

The trial treatment has only one route of administration:
Oral (rosuvastatin) - One tablet is to be administered daily, and may be given at any time of day, with or without food.

The daily dose of rosuvastatin should be given prior to the dose of IV tobramycin.

### 7.3.4 Dose Modifications

Rosuvastatin dose modifications will not be allowed. Those who show adverse effects as a result of the trial intervention may be withdrawn from the trial treatment. The decision to withdraw the patient from trial treatment is based on the patient's current clinical presentation. The decision to interrupt or discontinue trial therapy is at the discretion of the treating physician using their informed clinical opinion. Doses may be interrupted or discontinued at any point during the trial period for reasons such as unacceptable adverse effects, intercurrent illness, development of serious disease or any change in the patient's condition that the physician believes warrants a change in medication. Any changes must be documented in the CRF along with the justification for those changes. Follow-up should be continued until the end of the trial as per the study visit schedule. If the patient is withdrawn from trial treatment, the dispensed medications will be returned to pharmacy for disposal via their local procedures.

### 7.3.5 Duration of Treatment

For those in the treatment arm, treatment with rosuvastatin will continue for the full duration of the course of IV tobramycin received. Usually this will last for 14 days (14 once daily IV doses), in which case the participant would receive 14 once daily doses of rosuvastatin on each day of the IV tobramycin course. Treatment with oral rosuvastatin should continue for the duration of the course of IV tobramycin. Therefore, if the IV tobramycin course is shorter than 14 days, rosuvastatin should be discontinued after the final dose of IV tobramycin has been given. If the course of IV tobramycin is longer than 14 days, then additional oral rosuvastatin should be dispensed, in order to continue until the final day of the IV tobramycin course.

## 7.4 Unblinding

The study is an open label trial, therefore unblinding is not required. This is a phase IIa study, and we are using the change in an objective biomarker (KIM-1) as an end-point, which is not going to be affected by whether the trial is blinded or not. However, the laboratory measurement of KIM-1 will be blinded.

## 7.5 Accountability Procedures for Study Treatment/s

As the rosuvastatin used in the trial will be sourced via usual NHS procurement arrangements, pharmacy will liaise with the local procurement department to ensure that the site has the following in place and will report any problems to the study co-ordinator:

- A record of deliveries and dispensing of rosuvastatin - A system in place that allows for the retrieval if the manufacturer issues a recall - local procedures should be used.

- Enough rosuvastatin within shelf life assigned to be used in the study (see Section 7.2.3).

- Rosuvastatin is used in compliance with the protocol requirements and accountability records are maintained.

- Batch number of the product dispensed should be recorded on the prescription form, and these forms should be filed in a trial folder to permit retrospective verification.

## 7.6 Assessment of Compliance with Study Treatment/s

Once the participant has been informed of their treatment allocation, treatment compliance with the study protocol should be recorded in the study CRFs (this will include a daily record of the time of taking the study IMP, completed by the participant in their patient-held study diary. Where patients are still in hospital, the diary can also be completed by the research nurse). At the T+13/final day assessment, a count of any unused tablets will be performed, and these will be returned to the local site pharmacy

## 7.7 Concomitant Medications/Treatments

### 7.7.1 Medications Permitted

Details of concomitant medications will be collected at the screening visit (T0) and recorded on the CRF. They will be reviewed daily during admission for IV tobramycin, and at all subsequent study visits.

The participants in this study are likely to be taking a number of concomitant medications for the management of their CF. The trial treatment has few adverse interactions with other medicinal products, therefore concomitant medications, with the exception of those listed in Section 7.7.2, are permissible.

#### 7.7.1.1 Aluminium and magnesium hydroxide containing antacids

The SMPC for rosuvastatin states that Aluminium and magnesium hydroxide containing antacids can cause reduced absorption of rosuvastatin. However, this interaction can be avoided by ensuring that the antacid is given at least 2 hours after rosuvastatin. Providing this advice is followed, then participants on these antacids may participate in the study.

### 7.7.1.2 Clarithromycin and Azithromycin

Erythromycin is contraindicated with rosuvastatin, but the other macrolides are permitted. Unlike other statins, Rosuvastatin is not metabolised through CYP3A4. With other statins erythromycin results in increased plasma levels of the statin due to the interaction via CYP3A4. However, with Rosuvastatin erythromycin results in lower plasma levels, possibly secondary to it increasing gut motility. There is no published data on interactions between clarithromycin or azithromycin and rosuvastatin. However, there is not thought to be a class effect, and therefore there is no reason for concomitant use of these to be contraindicated.

### 7.7.1.2 Itraconazole

Itraconazole is a potent inhibitor of cytochrome P450 (CYP) 3A4, and is used fairly commonly in children with cystic fibrosis for prophylaxis and treatment of fungal infections. Several of the statins are primarily metabolised through this pathway, and co-administration with itraconazole results in significantly elevated plasma concentrations of these statins. Rosuvastatin, however, is only minimally cleared by metabolism, and this is primarily by CYP 2C9, with only a minor role for CYP3A4 and CYP2C19. A clinical trial has been done to assess the impact of itraconazole on the pharmacokinetics of rosuvastatin.[24] When rosuvastatin 10mg was coadministered with itraconazole, the resulting exposure to rosuvastatin was increased by 39%. Given the relatively low exposure ensuing from rosuvastatin 10mg in children[25] it is unlikely that increases in exposure of this degree would be clinically significant. Therefore concomitant use of itraconazole is permitted.

## 7.7.2 Medications Not Permitted

The following are not permitted for the duration of the trial period (from SmPC & BNFc):

1. **Ciclosporin** (Contraindicated with Rosuvastatin, SmPC)
2. **Protease Inhibitors** (strongly increase rosuvastatin levels, SmPC)
3. Fibrates (increase rosuvastatin levels, SmPC)
4. Ezetimibe (small increase in rosuvastatin level, SmPC)
5. Erythromycin, but not other macrolides (see 7.7.1.2, reduced rosuvastatin level, SmPC)
6. Eltrombopag (increase rosuvastatin levels, SmPC)
7. Dronedarone (increase rosuvastatin levels, SmPC)
8. Coumarins (increased anticoagulant effect with rosuvastatin, SmPC)
9. Oral contraceptives (increased levels of ethinylestradiol, norgestimate, and norgestrel with rosuvastatin, SmPC)
10. nicotinic acid (BNFc)
11. fusidic acid (BNFc)
12. Simepravir (increase rosuvastatin levels, SmPC)

### 7.7.3 Precautions required

**Pregnancy:**

A pregnancy test should be carried out in all female participants of childbearing potential (all who have reached menarche) at the time of enrolment to the study before taking rosuvastatin. If a potential participant refuses the test, they should not be included in the study. Rosuvastatin is not recommended in pregnancy; therefore, for those who are on the treatment arm, barrier contraception must be *strongly* advised to all women of childbearing age for the duration of the trial, if sexually active. Sensitivity will need to be exercised by the researcher addressing this issue with the potential participant, and care may be required to avoid embarrassment to the child in front of their parents. An appropriate approach should be discussed beforehand with the clinical team who know the patient and their family. This issue has been discussed with the CRN: Children, and is in line with their recommendations.

### 7.7.4 Data on Concomitant Medication

The dose and name of all concomitant medications, including over the counter and alternative medicines should be documented on the CRF at T0. This will be reassessed on a daily basis during tobramycin treatment, and at each follow-up visit by the PI/RN. Any new medications introduced or any changes to current medications should be documented on the CRF.

## 7.8 Co-enrolment Guidelines

To avoid potentially confounding issues, patients recruited into another CTIMP should not be enrolled in this study. Where recruitment into another study is considered to be appropriate and without having any detrimental effect on this study, this must first be discussed with the Chief Investigator. We have received confirmation from the TORPEDO-CF trial Co-Chief Investigator (Professor Alan Smyth) that patients already taking part in the TORPEDO-CF trial may be approached about participation in the PROteKT trial but only when a period of six months have lapsed since patients were randomised onto the TORPEDO-CF trial. If the same patient can be approached for either trial, then the TORPEDO-CF trial should take precedent over PROteKT since the eligibility criteria is more restrictive in TORPEDO-CF.

## 7.9 Pharmacy Manual

Further details will be provided to each participating site in a PROteKT study Pharmacy Manual.

# 8 ASSESSMENTS AND PROCEDURES

Participating centres will be expected to each maintain a file of essential trial documentation (Site File), and copies of all completed case report forms (CRFs) for the trial. Data collection will use paper CRFs.

All paper CRFs should be completed by personnel named on the delegation log as authorised to do so. Once completed, original CRFs should be sent to the CTRC, and copies should be kept in the local site file.

Participant details including name, initials and date of birth will be reported on the consent form, separate to clinical data. Once written informed consent has been obtained from the participant, the participant will be invited to attend a baseline screening visit on the day of admission for IV tobramycin treatment. At this visit, consent will be confirmed, the RN will collect the baseline characteristics, and the participant will be randomised and followed-up in the trial. For screening, baseline and randomisation procedures refer to section 6. For details of procedures associated with trial treatments refer to section 7.Data similar to that collected at baseline (see section 6.3) should be recorded on the appropriate follow-up CRFs.

## 8.1 Schedule for Follow-up

See schedule of study procedures, Table 1.

Patients with CF may receive their treatment course of IV tobramycin as an inpatient or at home. Participants should attend for their baseline (T0) visit as described, before commencing treatment with IV tobramycin. They may then either be admitted or will go home to commence their IV tobramycin. Daily urine sample collections will be completed either on the inpatient ward, or at home (see section 8.2.1). Assessments at T+1, T+8 and T+13 where blood sample collection is required will normally require the subject to attend the study site, unless facilities exist for bloods and lung function to be done at the patient's home. The T+1 assessment should be completed on that day. The T+8 assessment should be completed between days T+7 and T+9 inclusive (allowing it to be planned to coincide with clinically indicated bloods more easily). The T+13 assessment should be completed on day T+13 or the final day of tobramycin treatment if this occurs earlier. If treatment with IV tobramycin extends beyond 14 days, a further assessment, completing the same information as the T+13 day assessment, should be completed on the final day of therapy, and daily assessments should be continued until this point.

Scheduled study visits are designed to fit with routine hospital visits where possible. The 4 week follow-up visit should be conducted between 3 and 5 weeks after the completion of tobramycin treatment. Participants withdrawn from trial treatment will be asked to continue with scheduled follow-up visits. If the participant misses the scheduled follow-up visit, the RN should conduct the follow-up at the earliest convenient date. If a participant does not wish to continue in the study, a withdrawal CRF will be completed to capture the date and reason for trial withdrawal as detailed in section 5.3.3.

### 8.1.1 Scheduled Assessments

Baseline assessments will be completed at the T0 visit as described in section 6.3. During treatment with IV tobramycin the T+1, T+8 and T+13 assessments will include a review of concomitant medications, assessment of adverse events, a symptom-directed physical examination if required, and collection of a urine sample for biomarker analysis.

Urine samples for biomarker analysis will be collected daily during treatment with IV tobramycin as described in section 8.2.1. If the participant is an inpatient, samples will be refrigerated and then sent to the local laboratory for processing and storage. If the participant is receiving IV tobramycin at home, samples will be stored in the home refrigerator in a sealed container until the next scheduled study visit (T+8 or T+13) when they will be sent to the local laboratory.

Participants will complete a daily diary during IV tobramycin treatment in which they will record the times of their IV tobramycin dose, rosuvastatin dose, and urine sample collection. They will also have a telephone number to contact the study team in case of any symptoms of a suspected adverse event.

Assessment at T+1day will include the routine daily assessment, plus collection of blood samples for analysis as described in sections 8.2-8.4.

Assessments at T+8 and T+13days, will include the routine daily assessment, plus height and weight measurements, measurement of FEV1, and collection of blood samples for analysis as described in sections 8.2-8.4.

The 4 week (+/- 1 week) follow-up visit will include collection of a urine sample for biomarker analysis, height and weight measurements, measurement of FEV1, and collection of blood samples for analysis as described in sections 8.2-8.4.

### 8.1.2 Unscheduled Assessments

If treatment with IV tobramycin extends beyond 14 days, a further assessment, completing the same information as the T+13 day assessment, should be completed on the final day of therapy.

If treatment with IV tobramycin is completed before 14 days, a final assessment (akin to the T+ 13 assessment) should be completed on the final day of therapy. Patients randomised to the treatment arm will also need to stop taking Rosuvastatin on the same day as IV Tobramycin is completed.

In circumstances where a decision is made to stop IV tobramycin which does not leave sufficient time to complete a final day assessment, the assessment may be completed the following day.

During IV tobramycin participants will be reviewed daily for symptoms or signs of adverse events. If there exists clinical concern of an adverse event, part of the assessment would include blood samples for all blood tests described in sections 8.2-8.4.

If additional blood samples are collected during IV tobramycin therapy, on days other than the T+1, T+8 and T+13 assessments, details should be completed on the relevant day’s CRF.

Following the completion of IV tobramycin and before the scheduled 4 week follow-up visit, some participants may need to attend for unscheduled study visits. In these instances, the PI or RN should complete the Unscheduled Visit CRF.

## 8.2 Procedures for Assessing Efficacy

Efficacy of trial treatments will be assessed throughout the period of the study using objective measures.

### 8.2.1 Assessment of Urine Biomarkers

The primary outcome measure for the trial will be fold-change in urinary KIM-1 during tobramycin exposure.

Urine samples will be collected from each child at baseline, and on each day of tobramycin treatment. A further sample will be collected at the 4 week follow-up visit.

The normally preferred method of collection will be a clean catch urine sample into a sterile container. Samples will then be transferred to (if not already collected in) a sterile universal sample container (white top).

If the participant is an inpatient, the daily samples must be stored at fridge temperature (4ºC) for 1 week and then sent to the local laboratory for processing and further storage at -80ºC (or -20 ºC for a maximum of 6 months). If the participant is receiving IV tobramycin at home, samples will be stored in the home refrigerator in a sealed container until the next scheduled study visit (T+8 or T+13). In the latter case, when the study team receive the samples, they need to ensure that they are stored at fridge temperature for a total of one week (including time spent at patient's home) and then sent to the local laboratory for processing and further storage at -80ºC (or -20 ºC for a maximum of 6 months). KIM-1 is stable in urine stored at 4ºC for up to 1 week.[24, 25]

Urine samples will be centrifuged and then aliquoted into smaller volumes at the local site. These aliquoted samples will then be frozen (at -80ºC or -20 ºC for a maximum of 6 months) and stored at the local site. Batched samples will be couriered on dry ice to a GCLP registered laboratory at the University of Liverpool for subsequent storage and analysis (here samples will be stored at -80ºC). All measurements of biomarkers will be undertaken in a standardised manner, with the laboratory analysis being blinded to timing of samples and clinical outcomes of patients. When samples are analysed for urine biomarkers, urinary creatinine will also be measured, as this is used to standardise urinary biomarker values. Analysis of the primary outcome measure, KIM-1, and urinary creatinine, will be undertaken using validated assays in a GCLP laboratory at the University of Liverpool. Analysis of other urinary biomarkers will be undertaken in other laboratories at the University of Liverpool. Samples will be kept until they have been used up.

### 8.2.2 Assessment of Serum creatinine

Serum creatinine measurements will be performed at T0 and T+1 day, T+8 days, T+13 days, and the 4 week follow-up visits. The blood sample mentioned in Section 8.4 will be analysed at the laboratory serving the local research site for this purpose.

## 8.3 Procedures for Assessing Safety

Adverse event reporting is detailed in Section 10 (Pharmacovigilance) and will occur from the point of randomisation and throughout the trial treatment period up until the final follow-up visit 3-5 weeks after the patient has taken the final dose of investigational medicinal product.

## 8.4 Other Assessments: Special Assays or Procedures

Blood samples will be collected from each participant at the T0, T+1 day, T+8 days, T+13 days, and the 4 week follow-up visits at the respective sites. A minimum of two 1.2ml samples in Lithium/Heparin blood tubes, and one 1.2ml sample in a serum blood tube will be collected at each time point. Plasma and serum will be extracted locally at each site, aliquoted and stored at -80ºC. Wherever possible, the timing of study bloods should coincide with any clinically indicated bloods being taken on that day in order to minimise the burden to the patient. The method of blood collection will most likely need to be by venepuncture given the blood volumes required.

### 8.4.1 Assessment of effect of statin on cholesterol

Serum lipid levels (total cholesterol, triglycerides, LDL-c and HDL-c) will be performed at T0 and T+1 day, T+8 days, T+13 days, and the 4 week follow-up visits. The blood sample mentioned above in Section 8.4 will be analysed at the laboratory serving the local research site for this purpose.

### 8.4.2 Assessment of effect of statin on liver and muscle

Liver Function Tests (Alanine transaminase (ALT), aspartate transaminase (AST), Alkaline Phosphatase, and bilirubin) and creatine kinase will be performed at T0 and T+1 day, T+8 days, T+13 days, and the 4 week follow-up visits; analysis will be carried out at the laboratory serving the local research site using the same blood sample as mentioned above (see section 8.4).

### 8.4.3 Assessment of novel serum biomarkers

Plasma and serum will be extracted from blood samples and will be stored at -80ºC until sent to the Wolfson Centre for Personalised Medicine, University of Liverpool for laboratory analysis of novel biomarkers. Samples will need to be sent on dry ice by courier.

### 8.4.4 Assessment of C-reactive protein

C-reactive protein will be performed at T0 and T+1 day, T+8 days, T+13 days, and the 4 week follow-up visits; analysis will be carried out at the laboratory serving the local research site using the same blood sample as mentioned above (see section 8.4).

### 8.4.5 Assessment of response to tobramycin

Forced Expiratory Volume in 1 second (FEV1) (percent predicted) will be measured using spirometry at T0 , T+8 days, T+13 days, and the 4 week follow-up visits.

### 8.4.6 Assessment of tobramycin concentrations

Tobramycin concentrations will be measured at T+1 day, T+8 days, and T+13 days; analysis will be carried out at the laboratory serving the local research site using the same blood sample as mentioned above (see section 8.4).

### 8.4.7 Assessment of rosuvastatin concentrations

For patients in the treatment arm of the study, Rosuvastatin concentrations will be measured at T+1 day, T+8 days, and T+13 days. Plasma and serum will be extracted from blood samples and will be stored at -80ºC until sent to the Wolfson Centre for Personalised Medicine, University of Liverpool for laboratory analysis of rosuvastatin concentrations. Samples will need to be sent on dry ice by courier.

### 8.4.8 Assessment of *Pseudomonas aeruginosa* biomarkers

Biomarkers including molecules associated with *Pseudomonas aeruginosa* quorum sensing can be used to determine both the presence of *P. aeruginosa* as well as markers of virulence. These markers will be quantified from the serum/plasma and urine samples sent to the University of Liverpool as described above.

### 8.4.9 Assessment of changes in sputum microbiome

Sputum samples will be collected at the baseline T0, T+8 days, T+13 days and the 4 week follow-up visit. Ideally a sample of greater than 200ul is required. If clinically indicated samples are being collected at these times, then the remainder of the sample can be kept for the study. Samples should be frozen at -80 ºC at the local site as soon as possible after collection. They will be stored at the local site until sent to the Wolfson Centre for Personalised Medicine, University of Liverpool for storage prior to laboratory analysis. Samples will need to be sent on dry ice by courier. These samples will be used for analysis of the sputum microbiome, and for investigation of the underlying mechanisms behind changes in the microbiome during exposure to tobramycin with or without rosuvastatin. This will include analysis of biomarkers of *Pseudomonas aeruginosa* quorum sensing in sputum as per 8.4.8. Bacteria will also be isolated from the samples for routine microbiology analysis such as typing and antibiotic susceptibility testing.

Cough swabs and Bronchoalveolar lavage (BAL) fluid if collected for clinically indicated reasons during the study, and sputum samples collected for clinical reasons at times other than those prescribed above, can also be stored for analysis as part of the study, providing accurate time and date of collection is provided. BAL samples should be frozen at -80 ºC in the same way as sputum samples.

Details of previous microbiology results and sensitivities of previously isolated organisms will be recorded at baseline. Any new results during the course of the study will also be recorded.

## 8.5 Additional Blood Tests

During the study period, participants may have blood samples taken for a clinically –indicated reason on days other than T+1, T+8 and T+13. In this case, any left-over blood samples would be used for the assessment of tobramycin and rosuvastatin concentrations.

## 8.6 PROteKT substudy - Molecular genetics of aminoglycoside-induced nephrotoxicity

### 8.6.1 DNA Collection

The aim is to collect 5ml of whole blood in an EDTA blood tube to allow for DNA extraction. At a minimum a blood sample should contain 2 ml. If a blood sample cannot be collected then a Saliva sample will be collected (2 x 2ml).

All of our participants will be having blood samples taken as part of the PROteKT study. Therefore, an additional blood sample, for DNA collection, will be taken at the same time as other study bloods. Usually this will be done at the Baseline Assessment (T0), but it can be taken at any study visit when other bloods are being done. However, if the patient does not wish to have an additional blood test carried out we will use a saliva sample.

### 8.6.2 Laboratory analysis

All samples will be posted/ delivered to the Wolfson Centre for Personalised Medicine, University of Liverpool. Here samples will be processed, DNA extracted and stored securely. We have instituted a new bar coding system in the Department that adds further security in terms of sample tracking and also for confidentiality purposes. All biological samples stored within the Department are in keeping with the Human Tissue Act (license held by the University of Liverpool).

The samples collected in this study will be combined with:

1. Samples collected through the MAGIC study (Molecular Genetics of Adverse Drug Reactions in Paediatric Patients), Research Ethics No: 10/H1002/57.

2. Samples collected through worldwide efforts to evaluate genetic factors predisposing to drug-induced renal injury.

In the future, we will conduct a genome wide association study (GWAS) to identify genetic risk factors for aminoglycoside-induced nephrotoxicity.

## 8.7 Loss to Follow-up

If any of the trial patients are lost to follow up, contact will be attempted through the RN and lead investigator at each centre. Wherever possible, information on the reason for loss to follow-up will be recorded (see section 5).

## 8.8 Trial Closure

The end of the trial is defined to be the date on which data for all participants has been finalised and all data has been entered onto the database with data entry privileges withdrawn (data lock).

## 8.9 Storage of Samples

The samples collected as part of the study will be kept for analysis until all of the samples are used up. New markers, or methods of analysis, may become available in the future. In order to maximise the utility of the samples collected, these will be stored by the research team at the University of Liverpool, for future analysis. If there is any remaining sample after all analysis has been completed, and no further analysis is anticipated, it will be disposed in accordance with the Human Tissue Authority's Code of Practice. Participants will be asked to complete a separate consent form to give permission for this as it was not included in the original consent form. Participants and their parents will be approached for consent at routine clinic visits or study follow-up visits by a member of the research team trained in taking consent. Parent and participant information sheets will be provided, and any questions answered. Consent forms will be completed by parents or by the participant if aged 16 or over. Children aged under 16 years will be asked to complete an assent form.

## Table 1: Schedule of study procedures

| | Pre-clinic | Clinic | Baseline T0 | Daily during IV Tobramycin | T+1day | T+8days (between day T+7 and T+9 | T+13days/Final day assessment | Follow-up visit (4 weeks after final dose of | Additional assessment in case of suspected |
|---|---|---|---|---|---|---|---|---|---|
| Screening for potential participants | x | | | | | | | | |
| Information sheet sent to patient | x | | | | | | | | |
| Identification of eligible patients | | x | | | | | | | |
| Signed Informed consent | | x | | | | | | | |
| Assessment of Eligibility Criteria | | x | | | | | | | |
| Confirmation of consent and eligibility criteria | | | x | | | | | | |
| Review of Medical History | | | x | | | | | | |
| Review of Concomitant Medications | | | x | | x | x | x | | |
| Urine pregnancy test | | | x | | | | | | |
| Randomisation | | | x | | | | | | |
| Physical Exam - Complete | | | x | | | | | | |
| Dispense study medication | | | x | | | | | | |
| Study Intervention | | | x | x | | | | | |
| Treatment diary | | | | x | | | | | |
| Physical Exam - Symptom-Directed | | | | | x | x | x | | x |
| Assessment of Adverse Events | | | | | x | x | x | | x |
| Check study compliance | | | | | x | x | x | | |
| Return unused medication | | | | | | | x | | |
| Height | | | x | | | x | x | x | |
| Weight | | | x | | | x | x | x | |
| Collection of urine sample for biomarker analysis | | | x | x | | | | x | |
| Measurement of FEV1 | | | x | | | x | x | x | |
| Blood samples: | | | x | | x | x | x | x | x |
| Serum Creatinine | | | x | | x | x | x | x | x |
| Lipid Profile | | | x | | x | x | x | x | x |
| Liver Function | | | x | | x | x | x | x | x |
| Creatine Kinase | | | x | | x | x | x | x | x |
| CRP | | | x | | x | x | x | x | x |
| Tobramycin | | | | | x | x | x | | x |
| Rosuvastatin (Treatment arm) | | | | | x | x | x | | x |
| Plasma biomarkers | | | x | | x | x | x | x | x |
| Sputum | | | x | | | x | x | x | x |
| Plasma sample | | | x | | x | x | x | x | x |
| Serum sample | | | x | | x | x | x | x | x |
| Sub study (blood or saliva) – optional | | | | | | x | | | |

NB: Cough/Upper Respiratory Tract (Throat) Swabs and Bronchoalveolar Lavage (BAL) fluid will only be collected for clinically indicated reasons.

# 9 STATISTICAL CONSIDERATIONS

## 9.1 Introduction

A separate and full statistical analysis plan will be developed prior to the final analysis of the trial. This would include verification of the assumption that fold-change in KIM-1 is normally distributed, and use of an appropriate transformation (logarithmic) if necessary.

## 9.2 Method of Randomisation

Participants will be randomised using a web-based randomisation tool. This will randomise equally between two arms using variable block randomisation.

## 9.3 Outcome Measures

### 9.3.1 Primary Outcome

The primary outcome measure will be the difference in mean fold-change in urinary KIM-1 from baseline to 'highest value' concentration during exposure to tobramycin between the rosuvastatin treated arm and control arm.

### 9.3.2 Secondary Outcomes

- Change in traditional markers of renal injury, serum creatinine and estimated Glomerular Filtration Rate, and change in novel urinary and plasma biomarkers of renal injury during tobramycin exposure between the rosuvastatin treated arm and the control arm.
- To assess safety of the intervention, we will compare the reported expected and unexpected serious adverse events between the rosuvastatin treated arm and the control arm. We will particularly focus on muscle adverse events which have been reported with rosuvastatin.
- We will assess for interaction between rosuvastatin and tobramycin:
    - Pharmacokinetically, by comparing tobramycin concentrations between the rosuvastatin treated arm and the control arm.
    - Pharmacodynamically, by comparing change in percent of predicted Forced Expiratory Volume in 1 second (FEV1) and change in CRP, between the rosuvastatin treated and control arms.
- In the rosuvastatin treated arm, collected blood samples will be analysed centrally for rosuvastatin concentration, in order to assess the pharmacokinetic profile of rosuvastatin in children with CF, to assess compliance, and to relate rosuvastatin concentrations to change in urinary KIM-1.
- We will assess the impact on *Pseudomonas aeruginosa* by measuring biomarkers associated with *P. aeruginosa* quorum sensing which can be used to determine both the presence of *P. aeruginosa* as well as markers of virulence. These markers will be compared between the rosuvastatin treated and control arms

## 9.4 Sample Size and Power Requirement

A power calculation for the study has been completed using the following assumptions: The fold-change in KIM-1 ('highest value' measurement following treatment / baseline) is normally distributed in each arm with a common standard deviation. A mean fold change in KIM-1 of

3.03, with a standard deviation of 1.84 was derived from an early analysis of samples in the URBAN CF study from 10 participants receiving a single course of treatment with tobramycin. The same data was also inspected to assess that the assumption of normality is reasonable. Using these assumptions and utilizing a 2-sample t-test, a sample size of 20 in each arm would have a power of 0.92 to detect a difference in fold-change between the groups of 2, at a two-sided significance level of 0.05. We plan to include 50 patients in the trial in order to compensate for loss to follow up.

## 9.5 Analysis Plan

The primary outcome will be analysed using the method of analysis of covariance (ANCOVA). The outcome measure will be the 'highest value' of KIM-1 during exposure to tobramycin and the covariates will be treatment group and the baseline KIM-1 value. A secondary analysis of urinary KIM-1 will investigate the difference in Area Under the Curve (AUC) of urinary KIM-1 during exposure to IV tobramycin between the rosuvastatin treated arm and control arm.To explore the secondary objectives of identifying change in biomarkers in the active arm in comparison to the control, linear mixed effect models will be used to fully exploit the serial nature of these outcomes. These models can estimate the effect of treatment and the timing of any effect on each biomarker while accounting for variability within patients of the various biomarkers. We will then be able to assess which biomarker has the largest and/or earliest change in response to treatment. The evaluation of beneficial and adverse biomarkers in relation to rosuvastatin treatment will be examined using joint modelling approaches accounting for informative loss to follow up or censoring. All modelling will be adjusted for the covariate effects. The secondary outcomes of pharmacokinetics will be analysed using the relevant modelling approaches. Expected and unexpected adverse events will be analysed as the standard MHRA guidelines. A separate and full statistical analysis plan will be developed prior to the final analysis of the trial.

## 9.6 PROteKT substudy - Molecular genetics of aminoglycoside-induced nephrotoxicity

### 9.6.1 Statistical Methods

The samples collected in this study will be combined with:

1. Samples collected through the MAGIC study (Molecular Genetics of Adverse Drug Reactions in Paediatric Patients), Research Ethics No: 10/H1002/57.
2. Samples collected through worldwide efforts to evaluate genetic factors predisposing to drug-induced renal injury.

Statistical methodology for genetic association studies is a rapidly developing field, and the most up to date methods will be applied to bring the most powerful statistical methods to bear on the data analysis, and thus extract the maximum information possible from the genotype data. A detailed statistical analysis plan will be prepared prior to starting the analysis.

Prior to the association analyses, a test for Hardy Weinberg equilibrium will be undertaken at each SNP, using Fisher's exact test. Any marker found to deviate significantly ($p<0.001$) will be flagged and the reasons for deviation explored. Population substructure will also be tested for, and adjusted for in the analysis if any is detected. The extent of missing genotype data per SNP and per patient will be examined and the reasons explored. Tests to ensure that any

missing genotype data is at random will also be conducted. Multiple imputation methods will be used should missing genotypes be extensive.

For assessing association between a SNP and the risk of an ADR, two tests for association will be undertaken to compare genotype frequencies between cases and controls. The first will be a Chi-squared test, which makes no assumption regarding the underlying mode of inheritance, and the second will be a Cochrane-Armitage test for trend, which assumes an additive mode of inheritance. In the event that it is necessary to adjust for the effect of potential confounding factors, two logistic regression models will be fitted – the first including covariates to represent the confounding factors only and the second including covariates to represent both the confounding factors and the SNP – and a likelihood ratio test used to assess for association. The regression analysis will be conducted twice under the two different assumptions regarding mode of inheritance. In addition to the p-value, the false discovery rate will be calculated to assess for statistical significance whilst accounting for the multitude of tests undertaken.

# 10 PHARMACOVIGILANCE

## 10.1 Terms and Definitions

The Medicines for Human Use (Clinical Trials) Regulations 2004 (SI 2004/1031) definitions:

**Adverse Event (AE)**
Any untoward medical occurrence in a subject to whom a medicinal product has been administered, including occurrences which are not necessarily caused by or related to that product.

**Adverse Reaction (AR)**
Any untoward and unintended response in a subject to an investigational medicinal product which is related to any dose administered to that subject.

**Unexpected Adverse Reaction (UAR)**
An adverse reaction the nature and severity of which is not consistent with the information about the medicinal product in question set out in:

In the case of a product with a marketing authorization, in the summary of product characteristics for that product

In the case of any other investigational medicinal product, in the investigator's brochure relating to the trial in question.

**Serious Adverse Event (SAE), Serious Adverse Reaction (SAR) or Suspected Unexpected Serious Adverse Reaction (SUSAR)**
Any adverse event, adverse reaction or unexpected adverse reaction, respectively, that:

- results in death
- is life-threatening* (subject at immediate risk of death)
- requires in-patient hospitalisation or prolongation of existing hospitalisation**
- results in persistent or significant disability or incapacity, or
- consists of a congenital anomaly or birth defect
- Other important medical events

*'life-threatening' in the definition of 'serious' refers to an event in which the patient was at risk of death at the time of the event; it does not refer to an event which hypothetically might have caused death if it were more severe.

**Hospitalisation is defined as an inpatient admission, regardless of length of stay, even if the hospitalisation is a precautionary measure for continued observation. Hospitalisations for a pre-existing condition, including elective procedures that have not worsened, do not constitute an SAE.

***Other important medical events that may not result in death, be life-threatening, or require hospitalisation may be considered a serious adverse event/experience when, based upon appropriate medical judgment, they may jeopardise the subject and may require medical or surgical intervention to prevent one of the outcomes listed in this definition

## 10.2 Notes on Adverse Event Inclusions and Exclusions

### 10.2.1 Trial specific definitions

- Myalgia and myopathy (including myositis) are expected adverse reactions related to rosuvastatin. In addition to monitoring for symptoms, we will also measure Creatine kinase as part of the study. If creatine kinase is increased above 4 times the upper limit of normal, rosuvastatin should be discontinued, and the local site should report as a serious adverse reaction. If creatine kinase is less than 4 times the upper limit of normal and muscular symptoms are absent or mild, this should be recorded as an adverse reaction, but rosuvastatin can be continued and the patient should be closely monitored. If muscular symptoms are severe and cause daily symptoms, even if creatine kinase is less than 4 times the upper limit of normal, rosuvastatin should be discontinued, and the local site should report as a serious adverse reaction.
- Increases in liver transaminases are an expected adverse reaction related to rosuvastatin. Liver transaminases will be measured as part of the study. If liver transaminases are increased above 3 times the upper limit of normal, after the start of rosuvastatin, then rosuvastatin should be discontinued, and the local site should report as a serious adverse reaction.
- Minor side-effects identified in SPC that if occur, the impact would be relatively non-substantial in this patient group: Headache, Dizziness, Constipation, Nausea, Abdominal pain, Myalgia, Asthenia, Pruritis, Rash, Urticaria. These should be recorded as Adverse Reactions.
- Side effects identified in the SPC that could have a substantial impact in this patient group: Thrombocytopenia, Hypersensitivity reactions including angioedema, Polyneuropathy, Memory loss, Diabetes Mellitus, Pancreatitis, Jaundice, Hepatitis, Rhabdomyolysis, Stevens-Johnson syndrome, Immune-mediated necrotising myopathy. These should be recorded and reported as Serious Adverse Reactions.

### 10.2.2 Include

- An exacerbation of a pre-existing illness
- An increase in frequency or intensity of a pre-existing episodic event/condition
- A condition (even though it may have been present prior to the start of the trial) detected after trial drug administration
- Continuous persistent disease or symptoms present at baseline that worsens following the administration of the study/trial treatment
- Laboratory abnormalities that require clinical intervention or further investigation (unless they are associated with an already reported clinical event).
- Abnormalities in physiological testing or physical examination that require further investigation or clinical intervention

### 10.2.3 Do Not Include

- Medical or surgical procedures- the condition which leads to the procedure is the adverse event
- Pre-existing disease or conditions present before treatment that do not worsen

- Situations where an untoward medical occurrence has occurred e.g. cosmetic elective surgery
- Overdose of medication without signs or symptoms
- The disease being treated or associated symptoms/signs unless more severe than expected for the patient’s condition

- Children with cystic fibrosis may require hospitalisation for exacerbations related to their condition. Therefore any readmission which occurs during the study pharmacovigilance period does not require expedited reporting as ‘serious’ unless it is felt there is evidence to suggest that exposure to rosuvastatin may have had a causative effect.

## 10.3 Reporting Procedures

Adverse reactions and all serious adverse events should be recorded. Depending on the nature of the event the reporting procedures below should be followed. Adverse reactions and all serious adverse events will be recorded from the point that the participant provides informed consent and throughout the trial treatment period up until the date of the follow-up assessment (3-5 weeks after the patient has taken the final dose of investigational medicinal product).

### 10.3.1 Non serious ARs/AEs

All adverse reactions (non-serious events suspected to be related to any dose administered of rosuvastatin) should be recorded, whether expected or not. Adverse reactions should be recorded on the study CRFs. Non-serious adverse events do not need to be reported.

### 10.3.2 Serious ARs/AEs/SUSARs

SARs, SAEs and SUSARs should be reported within 24 hours of the local site becoming aware of the event to the trial manager (scan report, encrypt and attach to email: protekt_trial@ucl.ac.uk). The SAE form asks for the nature of event, date of onset, severity, corrective therapies given, outcome and causality. The responsible investigator should sign the causality of the event. Additional information should be sent within 5 days if the reaction has not resolved at the time of reporting.

The trial manager will ensure that all serious adverse events are reviewed by the Chief Investigator or another senior clinician nominated by the Sponsor. For each serious adverse event, the CI, or his deputy, will make a judgment about whether the nature or severity of the circumstances surrounding the event are consistent with adverse events associated with the underlying condition, or with rosuvastatin. In the event of a serious adverse event that has a causal relationship to rosuvastatin and is not consistent with the information contained in the Summary of Product Characteristics the event will be labelled as a Suspected Unexpected Serious Adverse Reaction (SUSAR). All events labelled as SUSARs will be reported to the sponsor by the trial manager within 24 hours of the decision being made. All SUSARs will be reported by the trial manager to the competent regulatory authority (MHRA) in accord with the Sponsor’s Standard Operating Procedures. The trial manager should then supply a copy of the SUSAR to the CTRC in parallel with submitting to the MHRA.

All investigators will be informed of all SUSARs occurring throughout the study.

## 10.4 Responsibilities – Investigator

The Investigator is responsible for reporting all ARs that are observed or reported during the study. The Investigator is also responsible for reporting all SAEs observed or reported during the study, regardless of their relationship to study product.

All SAEs must be reported immediately by the investigator on an SAE form unless the SAE is specified in the protocol as not requiring immediate reporting.

## 10.5 Reporting of Pregnancy

Study participants will be tested for pregnancy as part of the trial screening process. Any pregnancy which occurs during the study should be recorded in the study CRF and the participant should be instructed immediately to stop taking study drugs. The sponsor should be informed. All pregnancies that occur during treatment need to be followed up until after the outcome using the SAE form. Consent to report information regarding these pregnancy outcomes should be obtained from the mother prior to completion and emailing of the SAE Form. Any SAE experienced during pregnancy must be reported on the SAE form. The investigator should contact the participant to discuss the risks of continuing with the pregnancy and the possible effect to the foetus. Appropriate Obstetric care should be arranged.

## 10.6 Notes on Severity / Grading of Adverse Events

The assignment of the severity/grading should be made by the investigator responsible for the care of the participant. Regardless of the classification of an AE as serious or not, its severity must be assessed according to medical criteria alone using the following categories:

**Grade 1 (Mild):** does not interfere with routine activities

**Grade 2 (Moderate):** Interferes with routine activities

**Grade 3 (Severe):** impossible to perform routine activities

A distinction is drawn between serious and severe AEs. Severity is a measure of intensity (see above) whereas seriousness is defined using the criteria in section 10.1, hence, a severe AE need not necessarily be a Serious Adverse Event.

## 10.7 Relationship to Trial Treatment

An initial assignment of the causality should be made by the investigator responsible for the care of the participant using the definitions below.

Following reporting, a further assessment of causality will be made by the Chief Investigator. In the case of discrepant views on causality between the Chief investigator and others, the MHRA will be informed of both points of view.

**Definitions of Causality**

**Unrelated**
There is no evidence of any causal relationship. N.B. An alternative cause for the AE should be given

**Unlikely**
There is little evidence to suggest there is a causal relationship (e.g. the event did not occur within a reasonable time after administration of the trial medication). There is another reasonable explanation for the event (e.g. the participant's clinical condition, other concomitant treatment).

**Possibly**
There is some evidence to suggest a causal relationship (e.g. because the event occurs within a reasonable time after administration of the trial medication). However, the influence of other factors may have contributed to the event (e.g. the participant's clinical condition, other concomitant treatments).

**Probably**
There is evidence to suggest a causal relationship and the influence of other factors is unlikely.

**Almost certainly**
There is clear evidence to suggest a causal relationship and other possible contributing factors can be ruled out.

## 10.8 Expectedness

An AE whose causal relationship to the study drug is assessed by the investigator as "possible", "probable", or "definite" is an Adverse Drug Reaction. All events judged by the designated investigator to be possibly, probably, or almost certainly related to the IMP, graded as serious and **unexpected** (see section 10.2 and SPC for list of Expected Adverse Events) should be reported as a SUSAR.

## 10.9 Follow-up After Adverse Events

All adverse events should be followed until satisfactory resolution or until the investigator responsible for the care of the participant deems the event to be chronic or the patient to be stable.

When reporting SAEs and SUSARs the investigator responsible for the care of the participant should apply the following criteria to provide information relating to event outcomes: resolved; resolved with sequelae (specifying with additional narrative); not resolved/ongoing; ongoing at final follow-up; fatal or unknown.

# 11 ETHICAL CONSIDERATIONS

## 11.1 Ethical Considerations

We consider the specific ethical issues relating to participation in this trial to be:

### 11.1.1 Informed consent process

The study will be conducted in a paediatric population. We will provide age-specific patient information leaflets, which will be sent by the clinical team to the patient's home address at least 24 hours prior to them being approached for consent. Consent will be requested from parent/guardian with child assent form for participants <16 years old. Participants aged 16-18 years will consent for themselves. Informed consent will be sought according to methods approved by an independent REC and local NHS management organisation, and will be in accordance with the Declaration of Helsinki. The researchers will have experience in taking informed consent from parents and children, and will have up-to-date GCP training.

### 11.1.2 Allocation of participants to control arm

Half of recruits will be allocated to the non-intervention control arm. These patients will not receive any investigational drug and therefore do not get any direct benefit of the intervention, if any; however such a non-intervention comparator arm is necessary for the identification of a positive drug effect in the treatment arm. This will have no impact on their standard CF treatment as rosuvastatin is used as an addition to standard care.

### 11.1.3 Risk of adverse effects

There are known adverse effects associated with rosuvastatin that are outlined in the SmPC. Whilst serious adverse effects are rare, we will monitor participants receiving rosuvastatin to identify these early.

### 11.1.4 Increased medication burden to the participants

There will be further increase in the medication burden to the participants of this trial. However, we do not envisage this to be a major issue since the intervention is available as a single tablet that needs to be taken only once daily for a period of only 2 weeks.

### 11.1.5 Contraception during the treatment period

A pregnancy test should be carried out in all female participants of childbearing potential (all who have reached menarche) at the time of enrolment to the study before taking rosuvastatin. If a potential participant refuses the test, they should not be included in the study. Rosuvastatin is not recommended in pregnancy; therefore, for those who are on the treatment arm, barrier contraception must be *strongly* advised to all women of childbearing age for the duration of the trial, if sexually active. Sensitivity will need to be exercised by the researcher addressing this issue with the potential participant, and care may be required to avoid embarrassment to the child in front of their parents. An appropriate approach should be discussed beforehand with the clinical team who know the patient and their family. This issue has been discussed with the NIHR Medicines for Children Research Network, and is in line with their recommendations.

### 11.1.6 Additional visits required for the trial

We have taken steps in designing this study to minimise the need for participants to attend for extra visits. Participants will need to attend for baseline assessment, but they would normally need to attend on this day for their IV line to be sited and bloods taken. During the treatment phase daily assessments and collection of urine samples, may occur on an inpatient unit, or through a home visit if the participants is having IV tobramycin at home. Blood samples may also be collected at home if this is normal local practice. The 4 week follow-up visit will usually be additional, although efforts will be made to combine this visit with another clinical visit if possible.

#### 11.1.7 Additional Blood tests

The protocol is designed so that the majority of study bloods will be collected at the same time as routine clinical bloods. However, it is anticipated that children participating in the study will receive two additional blood tests to those required for routine clinical care. When blood tests are done, an additional volume of at least 4mls of blood will be required. This is well within the volume limits recommended by the European Medicines Agency. If clinically indicated blood samples are being taken at other times, an additional sample will be taken for study bloods. Where possible we will seek to minimise the volumes of blood taken by using left-over blood from clinical samples.

#### 11.1.8 Conflict of interest

The members of the research team who complete the urine biomarker analysis will not be involved in the medical care of participants, and will be blinded to study arm allocation. Those involved in recruiting participants will be members of the team providing medical care to participants.  There are no other conflicts of interest.

### 11.2 Ethical Approval

The trial protocol will be submitted to a multi-centre Research Ethics Committee (MREC) and will also undergo independent review at the R&D offices at participating sites. The local R&D office should be sent the appropriate site specific information form complete with the necessary authorisation signatures, plus any other documentation requested for review. Local Research & Development (R&D) approval is required before the site is initiated and patients recruited.

Consent from the patient should be obtained prior to participation in the trial, after a full explanation has been given of the treatment options, including the conventional and generally accepted methods of treatment. Patient Information and Consent Forms should also be implemented. The right of the patient to refuse to consent to participate in the trial without giving a reason must be respected. After the patient has entered the trial, the clinician must remain free to give alternative treatment to that specified in the protocol, at any stage, if he/she feels it to be in the best interest of the patient. However, the reason for doing so should be recorded and the patient will remain within the trial for the purpose of follow-up and data analysis according to the treatment option to which they have been allocated. Similarly, the patient remains free to withdraw at any time from the protocol treatment and trial follow-up without giving reasons and without prejudicing their future treatment.

### 11.3 Informed Consent Process

Informed consent is a process initiated prior to an individual agreeing to participate in a trial and continues throughout the individual’s participation. In obtaining and documenting informed

consent, the investigator should comply with applicable regulatory requirements and should adhere to GCP and to the ethical principles that have their origin in the Declaration of Helsinki.

Discussion of objectives, risks and inconveniences of the trial and the conditions under which it is to be conducted are to be provided to patients and their parents by staff with experience in obtaining informed consent. Information sheets which outline the trial and possible risks and benefits will be sent to the patient and their parents/guardian at least 24 hours prior to their routine clinic visit once they are identified by the delegated clinical staff at respective recruitment centres to meet the eligibility criteria. This will enable the patient and their parent/guardian to go through relevant information pertaining to the trial and associated risks and benefits in advance. Patient information sheets describing in detail the trial interventions/products, trial procedures and risks will be approved by an independent ethical committee and the patient will be asked to read and review the document.

Potentially eligible participants will be identified by the clinical team at each centre via a search of the patient database/s either electronically or manually or clinic list review. A short introductory letter about the study, along with a parent information sheet and age-appropriate information sheet for the child, will be sent out to the family at least 24 hours prior to their clinic visit. The introductory letter will ask them to read the information sheets, and will explain that they will be approached by the research team when they come to the clinic to consider consenting to participate in the study.

A situation may arise where a potential participant is identified in clinic, but has not previously received the study information in the post. In this instance the patient should be given the study information during their outpatient appointment and may then and may be approached for consent at their next outpatient appointment. However, if they are due to commence treatment with tobramycin before their next outpatient appointment, then they may be approached for consent on the day of attendance to commence their IV antibiotics. Consent may be obtained within 24 hours but only on the rare occasion when a potential participant is due to commence treatment with tobramycin within 24 hours of attending their outpatient appointment, and where they have not previously been sent the study information.

In the CF outpatient clinic, children (and their parents/guardian) who are eligible will be approached by a member of the research team. If they have not had opportunity before coming to the clinic, parents and children will be given time to read the study information sheets. The designated member of the research team will explain the trial to the patient. This information will emphasise that participation in the trial is voluntary and that the participant may withdraw from the trial at any time and for any reason. They will discuss the objectives of the study and all potential benefits and inconveniences of taking part. They will clearly outline all of the responsibilities the patient will be expected to meet if they agree to participate, including attendance at study visits and compliance with trial medications. All participants and their parents/guardian will be given opportunity to ask any questions that may arise, and will have the opportunity to discuss the study and time to consider the information prior to agreeing to participate. A contact point where further information about the trial may be obtained will be provided.

The parent (or the patient if aged 16 or over) will sign and date the informed consent document. The person taking consent must also personally sign and date the form. A copy of the informed consent document will be given to the patient for their records. The original copy will be filed in the participant's notes and a further copy of the signed consent form will be stored in the Investigator Site File. An assent form will be completed by the child if aged under 16 years. This consent will be considered 'prospective' and will be re-confirmed when the child attends for their baseline assessment. If a child aged 15 years has given assent and their parent has given consent then the child will not be re-consented if they reach the age of 16 years during the short follow up period unless they request to do so.

The researcher delegated to obtain informed consent will be determined on a site by site basis, depending on the experience and knowledge of the individual staff at that site. Only personnel deemed competent to do so by the PI and delegated the duty on the site signatory and delegation log will be able to obtain informed consent. This can include PIs, other delegated investigators and RNs. Where informed consent is being obtained by a RN, the patient should have access to a clinician with expertise in management of children with cystic fibrosis if they have any concerns about participation or any further questions that the RN is unable to sufficiently answer.

Where the informed consent discussion is conducted through a translator (i.e. the patient is non-English speaking), the translator will also sign the consent form to confirm that a full and accurate account of the study information has been provided to the patient.

Patients will be given sufficient time to consider their decision and consent to the trial. The patient may, without being subject to any resulting detriment, withdraw from the trial at any time by revoking the informed consent. The rights and welfare of the patients will be protected by emphasising to them that the quality of medical care will not be adversely affected if they decline to participate in this study.

## 11.4 Study Discontinuation

In the event that the study is discontinued, participants will be treated according to standard clinical care. The process for participants who withdraw early from trial treatment or from the trial completely is described in Section 5.3.

# 12 TRIAL MONITORING

## 12.1 Risk Assessment and Trial Monitoring Plan

Monitoring procedures for the PROteKT trial will be determined from the trial specific risk assessment. The risk assessment exercise will inform how monitoring should be conducted and the details of the monitoring activities will be documented in the related trial specific monitoring plan. The risk assessment is completed in partnership with the Chief Investigator, Sponsor and CTRC.

Guidance issued by the MRC, Department of Health and the MHRA on risk-adapted approaches to the management of CTIMPs propose a three level categorisation for the potential risk associated with the IMP, assigned according to the following categories:

***Type A*** *'no higher than that of standard medical care';*
***Type B*** *'somewhat higher than that of standard medical care';*
***Type C*** *'markedly higher than that of standard medical care'.*

Rosuvastatin is indicated for clinical use in the treatment of hypercholesterolaemia. However, in this study rosuvastatin will be used outside the manufacturer's indication, and therefore the risk associated with the IMP in this trial is categorised as ***Type B 'somewhat higher than that of standard medical care'***. This level of risk informs the risk assessment, regulatory requirements, nature and extent of the monitoring, and the management processes used in the trial.

### 12.1.1 What are the particular hazards of the trial?

Rosuvastatin is indicated for clinical use in the treatment of hypercholesterolaemia. However, in this study rosuvastatin will be used outside the manufacturer's indication, and therefore the risk associated with the IMP in this trial is categorised as ***Type B 'somewhat higher than that of standard medical care'***. This level of risk informs the risk assessment, regulatory requirements, nature and extent of the monitoring, and the management processes used in the trial.

Important factors for consideration include:

- The study will be conducted in a paediatric population, therefore close monitoring of consent/assent procedures will be a priority.
- A robust, centralised, web-based randomisation tool will be used to avoid the potential for compromise of random allocation in this open-label trial.
- Rosuvastatin has some known potential adverse effects. Rare but serious adverse effects involve skeletal muscle and the liver. We will monitor specifically for these by measuring creatine kinase and liver function tests respectively.
- Rosuvastatin will be used in a population who have normal lipid profiles, which may potentially result in transient hypolipidaemia. We are not aware of any evidence of adverse effects related to short-term hypolipidaemia, but this will be routinely monitored in all patients during the trial.

## 12.2 Source documents

***Source data****: All information in original records and certified copies of original records of clinical findings, observations, or other activities in a clinical trial necessary for the reconstruction and evaluation of the trial. Source data are contained in source documents (original records or certified copies). (ICH E6, 1.51).*

***Source documents****: Original documents, data, and records (e.g., hospital records, clinical and office charts, laboratory notes, memoranda, subjects diaries or evaluation checklists, pharmacy dispensing records, recorded data from automated instruments, copies or transcriptions certified after verification as being accurate copies, microfiches, photographic negatives, microfilm or magnetic media, x-rays, subject files, and records kept at the pharmacy, at the laboratories and at medico-technical departments involved in the clinical trial). (ICH E6, 1.52).*

In order to resolve possible discrepancies between information appearing in the CRF and any other participant related documents, it is important to know what constitutes the source document and therefore the source data for all information in the CRF. The following data recorded in the CRF should be consistent and verifiable with source data in source documents *other* than the CRF (e.g. medical record, laboratory reports and nurses' notes).

The following parameters that will be documented in the CRF are not source data:

- Relevant medical history and diagnosis (medical notes are source documents)
- Data for evaluation of eligibility criteria (medical notes are source documents)
- Physical examinations and assessments (medical notes are source documents).
- Concomitant medications (including changes) and diagnoses (medical notes are source documents)
- Dispensing of trial medication (pharmacy records are source documents)
- Adverse events (medical notes are source documents)

For data where no prior source documentation exists and which will be recorded directly in the CRF the CRF will be considered the **source document.**

In addition to the above source documentation the date(s) of conducting informed consent discussion, date of provision of patient information, randomisation number, treatment allocation and the fact that the patient is participating in a clinical trial should be added to the patient's medical record.

## 12.3 Data Capture Methods

Data will be collected on paper case report forms (CRFs) and via participant completed diaries. CRFs will be sent into CTRC for data entry into the study specific database by members of the Data Management Staff delegated with data entry responsibilities.

## 12.4 Central Monitoring

At the time of data entry data will be checked for missing or unusual values (range checks) and these will be raised as data queries with the centres. Data queries will be issued to centres in order to address problematic/missing data. Central monitoring reports will highlight where there are issues with missing data, non response to data queries and missing CRFs. The frequency for producing central monitoring reports will be determined from the risk assessment and specified in the monitoring plan.

## 12.5 Clinical Site Monitoring

The trial manager from UCL and staff from CTRC will be conducting routine on-site monitoring visits for the PROteKT trial. In addition there may also be 'triggered' visits to centres should central monitoring highlight any issues.

The specific monitoring activities to be carried out at site visits will be described in the monitoring plan. As members of the CTRC and the trial manager at UCL will need to access patient records, laboratory reports and other confidential medical information this fact is included in the patient information sheet and consent form.

## 12.6 Confidentiality

UCL will be undertaking activities requiring the transfer of some identifiable data: Verification that appropriate informed consent is obtained will be enabled by the provision of participant's signed informed consent form supplied to the trial manager at UCL by recruiting sites. This involves the transfer of participant names. This process is disclosed in the consent form. UCL will preserve the confidentiality of participants taking part in the study. Completed consent forms will be stored securely in a location separate to the clinical data collected.

## 12.7 Records Retention

The investigator at each investigational site must make arrangements to store the essential trial documents, (as defined in Essential Documents for the Conduct of a Clinical Trial (ICH E6, Guideline for Good Clinical Practice)) including the Investigator Site File and Pharmacy Site File, until the CTRC informs the investigator that the documents are no longer to be retained, or for a maximum period of 15 years (whichever is soonest). In addition, the investigator is responsible for archiving of all relevant source documents so, that the trial data can be compared against source data after completion of the trial (e.g. in case of inspection from authorities). The investigator is required to ensure the continued storage of the documents, even if the investigator, for example, leaves the clinic/practice or retires before the end of required storage period. Delegation must be documented in writing.

The CTRC undertakes to store originally completed CRFs, except for source documents pertaining to the individual investigational site, which are kept by the investigator only. The CTRC will archive the documents in compliance with ICH GCP utilising the Records Management Service of the University of Liverpool. All electronic CRFs and trial data will be archived onto an appropriate media for long term accessible storage. Hard copies of data will be boxed and transferred to specially renovated, secure, premises where unique reference numbers are applied to enable confidentiality, tracking and retrieval.

# 13 RESEARCH GOVERNANCE, SPONSORSHIP AND FINANCIAL ARRANGEMENTS

## 13.1 Research Governance

### 13.1.1 Sponsorship

PROteKT will be sponsored by The Joint Research Office, which has been set up by Liverpool Health Partners, a collaboration between the University of Liverpool and 9 NHS Trusts.

### 13.1.2 Trial support

The PROteKT study will be run with the input of the Clinical Trials Research Centre (CTRC), University of Liverpool, and a Trial Manager has been appointed, based at University College London (UCL). The CTRC is a fully registered Clinical Trials Unit (CTU) with the UKCRC and the national Medicines for Children trials unit. This CTU has extensive experience in conducting multi-centre clinical trials including investigational medicinal products regulated by the Medicines and Health care Products Regulatory Agency. The study will be adopted onto the NIHR portfolio and will receive support from the CRN: Children.

### 13.1.3 Independent Data and Safety Monitoring Committee (IDSMC)

The IDSMC will comprise of a statistician, clinician specialising in care of patients with CF, and a chairperson who are independent of the applicants and have no other involvement in the trial. The IDSMC will be responsible for reviewing and assessing recruitment, interim monitoring of safety and effectiveness, trial conduct and external data. The IDSMC will first convene prior to trial initiation and will then define frequency of subsequent meetings (at least annually). The IDSMC will provide a recommendation to the Trial Management Group concerning the continuation of the study.

### 13.1.4 Day to day trial management

The Trial Management Group (TMG) will be responsible for the day-to-day running and management of the trial; they will meet monthly throughout the trial. The Chief Investigator (MP) will meet with other trial staff (e.g. Trial manager, data manager, trial administrator, and trial statisticians) as required. We will also include a patient or parent representative onto this group in order to provide advice on patient recruitment, if one can be identified.

Recruitment and quality of data will be monitored by the trial team:

The data manager will be responsible for chasing overdue CRFs and following-up data queries until resolution as per routine practice.

The data manager alongside the trial manager and statistics team will also contribute towards the production of central monitoring reports. Typically these reports will highlight:

- Adverse event reporting rates between centres
- Protocol violations between centres
- Missing critical data items between centres
- Screening, recruitment and dropout rates between centres

These central monitoring reports will be reviewed by the TMG and, in accordance with the monitoring plan, will identify when additional intervention, e.g. triggered site visits, should be undertaken

The CTRC has processes in place to ensure that the trial will not open to recruitment until appropriate approvals and authorisations have been obtained from the MHRA, independent REC, and NHS Research & Development departments.

### 13.1.5 Trial Management division of responsibilities

The trial manager, based at UCL, will undertake the administration of all safety reports from the PIs at sites and provide data to the CTRC for the Independent Data and Safety Monitoring Committee. The IDSMC, and TMG meetings will be organised by the trial manager. CTRC SOPs will be used for trial management. The trial master file will be at UCL, who will provide a truncated TMF for the CTRC (e.g. ethics and CTA, contracts, current protocol etc).

CTRC will provide support and advice for efficient recruitment, robust data management, quality control, statistical analysis, effective monitoring of recruitment targets and reporting activities in compliance with governance requirements. CTRC SOPs will be used for statistics and data management.

The trial manager and CI should produce the Development Safety Update Report (DSUR). A template DSUR report document and the data required for summary tables can be agreed up front with the CTRC. SUSARs will be submitted to UCL (trial manager) for processing. The trial manager will liaise with the CI for medical oversight and ensure any expedited reports are submitted appropriately. The trial manager should then supply a copy of the SUSAR to the CTRC in parallel with submitting to the MHRA. CTRC Statisticians will provide summary data and contribute to the report, and the clinical perspective will be led by the CI. Guidance on SUSARs will be available from a CTRC Senior Trial Manager. CTRC are not responsible for this reporting other than to ensure we have accurate and up to date data on trial participants for summarising for safety monitoring for IDSMC/DSUR.

## 13.2 Insurance

The University of Liverpool professional indemnity and clinical trials insurance, and NHS indemnity schemes, will apply as appropriate

## 13.3 Funding

Funding for the study has been awarded by The J P Moulton Charitable Foundation. Additional funding to support this study is available through the North West England MRC Clinical Pharmacology and Therapeutics Fellowship awarded to Dr S McWilliam.

# 14 REGULATORY APPROVAL

This trial falls within the remit of the EU Directive 2001/20/EC, transposed into UK law as the UK Statutory Instrument 2004 No 1031: Medicines for Human Use (Clinical Trials) Regulations 2004 as amended. This trial will be registered with the MHRA for a Clinical Trial Authorisation (CTA). The EUDRACT number is 2014-002387-32.

# 15 PUBLICATION

## 15.1 Publication Policy

The study findings will be published in an appropriate peer-reviewed journal.

# 16 Reference List


1. Cystic Fibrosis Trust: UK CF Registry Annual Data Report 2009, Bromley, Kent, 2011.

2. Bertenshaw, C, Watson, AR, Lewis, S, Smyth, A: Survey of acute renal failure in patients with cystic fibrosis in the UK. Thorax, 62: 541-545, 2007.

3. Smyth, A, Lewis, S, Bertenshaw, C, Choonara, I, McGaw, J, Watson, A: Case-control study of acute renal failure in patients with cystic fibrosis in the UK. Thorax, 63: 532-535, 2008.

4. Al-Aloul, M, Miller, H, Alapati, S, Stockton, PA, Ledson, MJ, Walshaw, MJ: Renal impairment in cystic fibrosis patients due to repeated intravenous aminoglycoside use. Pediatr Pulmonol, 39: 15-20, 2005.

5. Pedersen, SS, Jensen, T, Osterhammel, D, Osterhammel, P: Cumulative and acute toxicity of repeated high-dose tobramycin treatment in cystic fibrosis. Antimicrob Agents Chemother, 31: 594-599, 1987.

6. Mathews, A, Bailie, GR: Clinical pharmacokinetics, toxicity and cost effectiveness analysis of aminoglycosides and aminoglycoside dosing services. J Clin Pharm Ther, 12: 273-291, 1987.

7. Schmitz, C, Hilpert, J, Jacobsen, C, Boensch, C, Christensen, EI, Luft, FC, Willnow, TE: Megalin deficiency offers protection from renal aminoglycoside accumulation. J Biol Chem, 277: 618-622, 2002.

8. Servais, H, Van Der Smissen, P, Thirion, G, Van der Essen, G, Van, BF, Tulkens, PM, Mingeot-Leclercq, MP: Gentamicin-induced apoptosis in LLC-PK1 cells: involvement of lysosomes and mitochondria. Toxicol Appl Pharmacol, 206: 321-333, 2005.

9. Servais, H, Ortiz, A, Devuyst, O, Denamur, S, Tulkens, PM, Mingeot-Leclercq, MP: Renal cell apoptosis induced by nephrotoxic drugs: cellular and molecular mechanisms and potential approaches to modulation. Apoptosis : An International Journal on Programmed Cell Death, 13: 11-32, 2008.

10. Askenazi, DJ, Ambalavanan, N, Goldstein, SL: Acute kidney injury in critically ill newborns: what do we know? What do we need to learn? Pediatr Nephrol, 24: 265-274, 2009.

11. Karlowicz, MG, Adelman, RD: Nonoliguric and oliguric acute renal failure in asphyxiated term neonates. Pediatric nephrology (Berlin, Germany), 9: 718-722, 1995.

12. Bonventre, JV: Diagnosis of acute kidney injury: from classic parameters to new biomarkers. Contrib Nephrol, 156: 213-219, 2007.

13. Coca, SG, Yalavarthy, R, Concato, J, Parikh, CR: Biomarkers for the diagnosis and risk stratification of acute kidney injury: a systematic review. Kidney Int, 73: 1008-1016, 2008.

14. Han, WK, Bailly, V, Abichandani, R, Thadhani, R, Bonventre, JV: Kidney Injury Molecule-1 (KIM-1): a novel biomarker for human renal proximal tubule injury. Kidney Int, 62: 237-244, 2002.

15. Ichimura, T, Hung, CC, Yang, SA, Stevens, JL, Bonventre, JV: Kidney injury molecule-1: a tissue and urinary biomarker for nephrotoxicant-induced renal injury. American Journal of Physiology Renal Physiology, 286: F552-F563, 2004.

16. Zhou, Y, Vaidya, VS, Brown, RP, Zhang, J, Rosenzweig, BA, Thompson, KL, Miller, TJ, Bonventre, JV, Goering, PL: Comparison of kidney injury molecule-1 and other nephrotoxicity biomarkers in urine and kidney following acute exposure to gentamicin, mercury, and chromium. Toxicological sciences : an official journal of the Society of Toxicology, 101: 159-170, 2008.

17. Vaidya, VS, Ozer, JS, Dieterle, F, Collings, FB, Ramirez, V, Troth, S, Muniappa, N, Thudium, D, Gerhold, D, Holder, DJ, Bobadilla, NA, Marrer, E, Perentes, E, Cordier, A, Vonderscher, J, Maurer, G, Goering, PL, Sistare, FD, Bonventre, JV: Kidney injury molecule-1 outperforms traditional biomarkers of kidney injury in preclinical biomarker qualification studies. Nat Biotechnol, 28: 478-485, 2010.

18. McWilliam, SJ, Antoine, DJ, Sabbisetti, V, Turner, MA, Farragher, T, Bonventre, JV, Park, BK, Smyth, RL, Pirmohamed, M: Mechanism-based urinary biomarkers to identify the potential for aminoglycoside-induced nephrotoxicity in premature neonates: A proof-of-concept study. PLoS ONE, 7, 2012.

19. McWilliam, SJ, Antoine, DJ, Sabbisetti, V, Pearce, RE, Jorgensen, AL, Lin, Y, Leeder, JS, Bonventre, JV, Smyth, RL, Pirmohamed, M: Reference intervals for urinary renal injury biomarkers KIM-1 and NGAL in healthy children. Biomarkers in medicine, 2014.

20. Antoine, DJ, Srivastava, A, Pirmohamed, M, Park, BK: Statins inhibit aminoglycoside accumulation and cytotoxicity to renal proximal tubule cells. Biochem Pharmacol, 79: 647-654, 2010.

21. Reagan-Shaw, S, Nihal, M, Ahmad, N: Dose translation from animal to human studies revisited. FASEB J, 22: 659-661, 2008.

22. Elis, A, Zhou, R, Stein, EA: Treatment of familial hypercholesterolaemia in children and adolescents in the last three decades. Cardiol Young: 1-5, 2013.

23. Birmingham, BK, Bujac, SR, Elsby, R, Azumaya, CT, Zalikowski, J, Chen, Y, Kim, K, Ambrose, HJ: Rosuvastatin pharmacokinetics and pharmacogenetics in Caucasian and Asian subjects residing in the United States. European Journal of Clinical Pharmacology, 71: 329-340, 2015.

24. Cooper, KJ, Martin, PD, Dane, AL, Warwick, MJ, Schneck, DW, Cantarini, MV: Effect of itraconazole on the pharmacokinetics of rosuvastatin. Clinical Pharmacology and Therapeutics, 73: 322-329, 2003.

25. Braamskamp, MJAM, Langslet, G, McCrindle, BW, Cassiman, D, Francis, GA, Gagné, C, Gaudet, D, Morrison, KM, Wiegman, A, Turner, T, Kusters, DM, Miller, E, Raichlen, JS, Wissmar, J, Martin, PD, Stein, EA, Kastelein, JJP: Efficacy and safety of rosuvastatin therapy in children and adolescents with familial hypercholesterolemia: Results from the CHARON study. Journal of Clinical Lipidology, 9: 741-750, 2015.

## Appendix 2: Statistical Analysis Plan – Version 4.0, 28th March 2018

**PROteKT (Phase IIa, Randomised, Controlled, Open-Label Trial of Rosuvastatin for the Prevention of Aminoglycoside-Induced Kidney Toxicity in Children with Cystic Fibrosis)**

**Eudract No. 2014-002387-32**

# Statistical Analysis Plan v4.0

| | ORIGINATED BY | QC PERFORMED BY | APPROVED BY |
|---|---|---|---|
| **Name** | Anna Rosala-Hallas | TBC | Ashley Jones |
| **Title** | Trial Statistician | QC Statistician | Senior Statistician |
| **Date** | 09/02/2018 | | |
| **Protocol Version and Date** | Version 8.0 on 06/02/2017 | | |

# 1. Change Control

| Protocol version | Updated SAP version no. | Section number changed | Description of change | Date changed |
|---|---|---|---|---|
| 8.0 | 2.0 | 6 | Protocol version 6.0 updated to version 8.0 | 31/08/2017 |
| 8.0 | 2.0 | 9.4 | Patient population changed from 10-18 to 6-18 as per protocol change.<br><br>Removal of Indian ancestry from exclusion criteria as per protocol change. | 31/08/2017 |
| 8.0 | 2.0 | 9.9 | Clarification added that the laboratory responsible for analysing KIM-1 will be blinded to treatment allocation. | 31/08/2017 |
| 8.0 | 2.0 | 13 | Clarification added that no adjustment for multiplicity will be used. | 31/08/2017 |
| 8.0 | 2.0 | 14.1 | Interim monitoring updated to describe IDSMC meetings that have taken place. | 31/08/2017 |
| 8.0 | 2.0 | 15.1 | Under reasons for ineligibility wording changed from 'Patient not aged 6-18' to 'Patient not within required age range' to account for the change in eligibility criteria as per protocol change.<br><br>Wording 'specify' changed to 'reasons will be reported'. | 31/08/2017 |
| 8.0 | 2.0 | 17 | Clarification added that the laboratory analysts will remain blinded throughout the duration of the trial. | 31/08/2017 |
| 8.0 | 2.0 | 18.1 | All numeric values will be presented to two decimal places | 31/08/2017 |
| 8.0 | 2.0 | 18.2 | Age also to be presented in categories as per EudraCT requirements.<br><br>Urinary NGAL added to baseline table. | 31/08/2017 |
| 8.0 | 2.0 | 18.4 | Clarification of how to normalise KIM-1 to creatinine. | 31/08/2017 |
| 8.0 | 2.0 | 18.4.2 | Addition of log-transformation given KIM-1 likely to be highly skewed.<br><br>Addition of text describing blind review of data by independent statisticians. | 31/08/2017 |
| 8.0 | 2.0 | 18.5.1.2<br>18.5.2.2<br>18.5.4.2 | Addition of mean and individual profile plots. | 31/08/2017 |
| 8.0 | 2.0 | 18.5.2.1 | Clarification on timing of urine and plasma samples added. | 31/08/2017 |
| 8.0 | 2.0 | 18.5.3.2 | Further details added to describe pharmacokinetic modelling | 31/08/2017 |
| 8.0 | 2.0 | 18.5.5.2 | Addition of scatter plots. | 31/08/2017 |
| 8.0 | 2.0 | 20 | Additional analysis of KIM-1 using AUC.<br><br>Addition of analysis of NGAL similar to that of the primary outcome added. | 31/08/2017 |
| 8.0 | 3.0 | 18.4 | Removal of reference to GCLP laboratory. | 09/02/2018 |
| 8.0 | 3.0 | 18.5.1.1 | Clarification that height is collected at T0, T+8 and T+13 days | 09/02/2018 |

| | | | | |
|---|---|---|---|---|
| 8.0 | 3.0 | 18.5.3.2 | Clarification added on pharmacokinetic modelling following advice from pharmacokinetic modelling expert blinded to the data. | 09/02/2018 |
| 8.0 | 3.0 | 21.2 | Clarification added that ARs and SAEs will be presented mutually exclusively. | 09/02/2018 |
| 8.0 | 4.0 | 18.4.2 | Removal of text referring to blind review as this has now taken place – see change below. | 27/03/2018 |
| 8.0 | 4.0 | 20 | Recommendations added for sensitivity analyses following blind review. | 27/03/2018 |

# 2. Approval and agreement

**SAP Version Number being approved:** 4.0

**Trial Statistician**

Name ______________________________

Signed ____________________ Date __________

**Senior Statistician**

Name ______________________________

Signed ____________________ Date __________

**Chief Investigator/clinical lead**

Name ______________________________

Signed ____________________ Date __________

## 3. Roles and responsibilities

A Rosala-Hallas (Department of Biostatistics, University of Liverpool), A Jones (Department of Biostatistics, University of Liverpool), S McWilliam (Institute of Translational Medicine, University of Liverpool).

**Author's contributions**

A Rosala-Hallas and A Jones proposed the statistical analysis plan. A Rosala-Hallas drafted the manuscript. A Jones and S McWilliam read, amended and approved the statistical analysis plan.

# 4. Contents


1. Change Control ........ 64
2. Approval and agreement ........ 66
3. Roles and responsibilities ........ 67
4. Contents ........ 68
5. List of abbreviations and definitions of terms ........ 71
6. Statement of Compliance ........ 72
7. Background and Rationale ........ 72
8. PROteKT Study Objectives ........ 73
9. Investigational Plan and Study Design ........ 73
    - 9.1. Overall study design and plan - description ........ 73
    - 9.2. Treatments studied ........ 73
    - 9.3. Treatment compliance ........ 73
    - 9.4. Patient population studied ........ 73
    - 9.5. Inclusion criteria ........ 73
    - 9.6. Exclusion criteria ........ 74
    - 9.7. Removal of patients from therapy or assessment ........ 74
    - 9.8. Consent process ........ 74
    - 9.9. Blinding ........ 74
    - 9.10. Method of assignment to treatment ........ 74
    - 9.11. Sequence and duration of all study periods ........ 74
    - 9.12. Schedule of assessments ........ 74
10. Listing of Outcomes ........ 76
    - 10.1. Primary outcome(s) ........ 76
    - 10.2. Secondary outcomes ........ 76
11. Determination of Sample Size ........ 76
12. Study Framework ........ 76
13. Confidence Intervals, p-values and Multiplicity ........ 76
14. Timing and Objectives of Interim and Final Analyses ........ 77
    - 14.1. Interim monitoring and analyses ........ 77
    - 14.2. Final analysis ........ 77
15. Disposition of Participants ........ 77

15.1. Screening, eligibility and recruitment ... 77
15.2. Post randomisation discontinuations ... 79
16. Protocol Deviations ... 79
17. Unblinding ... 80
18. Efficacy Evaluations ... 81
18.1. Data Sets Analysed ... 81
18.2. Demographic and Other Baseline Characteristics ... 81
18.3. Compliance with treatment ... 82
18.4. Analysis of primary outcome ... 82
18.4.1. Derivation ... 83
18.4.2. Analysis ... 83
18.5. Analysis of Secondary Outcomes ... 83
18.5.1. Change in serum concentration of creatinine and eGFR during tobramycin exposure between rosuvastatin arm and control arm ... 83
18.5.1.1. Derivation ... 83
18.5.1.2. Analysis ... 84
18.5.2. Change in other urinary and plasma biomarkers of renal injury during tobramycin exposure between rosuvastatin arm and control arm ... 84
18.5.2.1. Derivation ... 84
18.5.2.2. Analysis ... 84
18.5.3. Difference in tobramycin concentrations between rosuvastatin treated arm and the control arm to identify any pharmacokinetic interaction between rosuvastatin and tobramycin 84
18.5.3.1. Derivation ... 84
18.5.3.2. Analysis ... 85
18.5.4. Difference in Forced Expiratory Volume in 1 second (FEV1) and C-Reactive Protein, between rosuvastatin treated arm and the control arm to identify any pharmacodynamic interaction between rosuvastatin and tobramycin ... 85
18.5.4.1. Derivation ... 85
18.5.4.2. Analysis ... 85
18.5.5. Relationship between plasma rosuvastatin concentrations achieved in children randomised to the intervention arm and change in urinary KIM-1 ... 86
18.5.5.1. Derivation ... 86
18.5.5.2. Analysis ... 86
18.5.6. Difference in biomarkers of *Pseudomonas aeruginosa* between rosuvastatin treated arm and the control arm ... 86

18.5.6.1. Derivation ........ 86
18.5.6.2. Analysis ........ 86
19. Missing data and withdrawals ........ 86
20. Additional analyses ........ 87
21. Safety Evaluations ........ 87
21.1. Data sets analysed ........ 87
21.2. Presentation of the data ........ 88
22. Quality Control ........ 88
23. References ........ 88

# 5. List of abbreviations and definitions of terms

| | |
|---|---|
| AR | Adverse reaction |
| CF | Cystic Fibrosis |
| CRF | Case report form |
| CRP | C-Reactive protein |
| CONSORT | Consolidated Standards of Reporting Trials |
| CTIMP | Clinical trial of an investigational medicinal product |
| CTRC | Clinical Trials Research Centre |
| CTU | Clinical Trials Unit |
| eGFR | Expected globular filtration rate |
| FEV1 | Forced expiratory volume in 1 second |
| HDL | High-density lipoprotein |
| LDL | Low-density lipoprotein |
| HIV | Human Immunodeficiency Virus |
| ICH | International Council for Harmonisation |
| IDSMC | Independent Data and Safety Monitoring Committee |
| IQR | Inter-quartile range |
| IMP | Investigational medicinal product |
| ITT | Intention to treat |
| IV | Intravenous |
| KIM-1 | Kidney Injury Molecule-1 |
| SAE | Serious adverse event |
| SAP | Statistical analysis plan |
| SD | Standard deviation |
| SOP | Standard operating procedure |
| TSC | Trial Steering Committee |

# 6. Statement of Compliance

This Statistical Analysis Plan (SAP) provides a detailed and comprehensive description of the pre-planned final analyses for the study “PROteKT” . The planned statistical analyses described within this document are compliant with those specified in brief within the PROteKT protocol v8.0 06/02/2017.

This study is carried out in accordance with the World Medical Association Declaration of Helsinki (1964) and the Tokyo (1975), Venice (1983), Hong Kong (1989) and South Africa (1996) amendments and will be conducted in compliance with the protocol, Clinical Trials Research Centre (CTRC) Clinical Trials Unit (CTU) Standard Operating Procedures (SOPs) and EU Directive 2001/20/EC, transposed into UK law as the UK Statutory Instrument 2004 No 1031: Medicines for Human Use (Clinical Trials) Regulations 2004.

These planned analyses will be performed by the trial statistician. This study is a clinical trial of a medicinal product and is registered on the EudraCT database. The statistical analysis plan has been developed to support the posting of results on the EudraCT system. This is a regulatory requirement which should be fulfilled within 6 months after the end of the study as defined within the clinical trial protocol.

The results of the final analysis described within this statistical analysis plan will be contained within a statistical analysis report. This report will be used as the basis of the primary research publications according to the study publication plan.

All analyses are performed with standard statistical software (SAS v9.3 or later). The finalised analysis datasets, programs and outputs will be archived following Good Clinical Practice guidelines and SOP TM021 Archiving procedure in CTRC. The testing and validation of the statistical analysis programs will be performed following SOP ST001.

# 7. Background and Rationale

The full background and rationale for the trial can be found in section 2.1 of the protocol. To be brief, Cystic Fibrosis (CF) is a common, inherited, life-limiting disease which affects around 9000 people in the UK alone. 25% of children with CF aged 12-15 years have chronic pulmonary infection, and 40% by age 16-19 years. There is improved survival with intravenous (IV) antibiotics, however, these can be potentially nephrotoxic. Current strategies for the prevention of nephrotoxicity are only partially effective. PROteKT will test the hypothesis that taking rosuvastatin alongside the IV

aminoglycoside antibiotic, tobramycin, can inhibit aminoglycoside nephrotoxicity in children with cystic fibrosis.

# 8. PROteKT Study Objectives

The primary and secondary objectives can be found listed in the protocol. The null hypothesis is that there is no difference in mean fold-change in urinary KIM-1 from baseline to peak concentration during exposure to tobramycin between the rosuvastatin treated arm and the control arm. The alternate hypothesis is that there is a difference between the two groups.

# 9. Investigational Plan and Study Design

## 9.1. Overall study design and plan - description

This study is a phase IIa, multi-centre, randomised, superiority, open-labelled trial of rosuvastatin in children with cystic fibrosis (CF) receiving clinically indicated treatment with intravenous (IV) tobramycin. Patients will be randomised equally to either receive rosuvastatin or no intervention (control).

## 9.2. Treatments studied

To summarise, those assigned to the treatment arm will receive a dose of oral rosuvastatin 10 (mg), once daily, for the duration of a treatment course of IV tobramycin (usually lasting 14 days). Those assigned to the control arm will not receive any medication alongside their tobramycin treatment.

## 9.3. Treatment compliance

Once the participant has been informed of their treatment allocation, treatment compliance with the study protocol will be recorded in the study CRFs (this will include a daily record of the time of taking the study IMP, completed by the participant in their patient-held study diary or completed by the research nurse in a treatment diary if participant is an inpatient). At the T+13/final day assessment, a count of any unused tablets will be performed, and these will be returned to the local site pharmacy.

## 9.4. Patient population studied

The patient population to be studied will include 50 children with cystic fibrosis aged 6 to 18 years receiving clinically indicated treatment with intravenous tobramycin, and who fulfil the inclusion criteria.

## 9.5. Inclusion criteria

The inclusion criteria can be found in section 5.1 of the protocol.

## 9.6. Exclusion criteria

The exclusion criteria can be found in section 5.2 of the protocol.

## 9.7. Removal of patients from therapy or assessment

Full details can be found in section 5.3 of the protocol.

## 9.8. Consent process

Full details on the consent procedure can be found in section 11.3 of the protocol. Consent is prospective and will take place before any trial specific tests or visits.

## 9.9. Blinding

PROteKT is an open label trial and hence patients will not be blinded to treatment allocation. However, allocation concealment is possible as participants will be randomised using a secure (24-hour) web-based randomisation programme controlled centrally by the CTRC CTU (see section 6.4 of the protocol for further details). The laboratory responsible for analysing KIM-1 will be blinded to treatment group during the analysis process.

## 9.10. Method of assignment to treatment

Participants will be randomised in a 1:1 ratio using variable block randomisation. Information on block sizes and stratification factors can be found in ST002TEM01 Randomisation Specification, filed in section 4 of the Statistics Trial File.

## 9.11. Sequence and duration of all study periods

A study flow chart can be found in section 1 of the protocol. To summarise, patients are randomised 1:1 to either the control or treatment arm. A course of IV tobramycin treatment is started and daily urine samples are taken. At T+1, T+8 and T+13 formal assessments take place where blood samples are taken. The final visit is scheduled for 4 weeks (+/- 7 days) after completing tobramycin treatment (usually lasting 14 days).

## 9.12. Schedule of assessments

A full schedule of assessments can be found in section 8 of the protocol. Table 8.12.1. is a condensed version detailing the times of assessments for the primary and secondary outcomes.

**Table 8.12.1**

| | Baseline T0 | Daily during IV Tobramycin | T+1day | T+8days (between day T+7 and T+9 inclusive) | T+13days/Final day assessment | Follow-up visit (4 weeks after final dose of Tobramycin) | Additional assessment in case of suspected adverse event |
|---|---|---|---|---|---|---|---|
| Collection of urine sample for biomarker analysis | x | x | | | | x | |
| Measurement of FEV1 | x | | | x | x | x | |
| Blood samples: | x | | x | x | x | x | x |
| Serum Creatinine | x | | x | x | x | x | x |
| Lipid Profile | x | | x | x | x | x | x |
| Liver Function | x | | x | x | x | x | x |
| Creatine Kinase | x | | x | x | x | x | x |
| CRP | x | | x | x | x | x | x |
| Tobramycin | | | x | x | x | | x |
| Rosuvastatin (Treatment arm) | | | x | x | x | | x |
| Plasma biomarkers | x | | x | x | x | x | x |
| DNA | x | | | | | | |
| Sputum | x | | x | x | x | | |

## 10. Listing of Outcomes

### 10.1. Primary outcome(s)

The primary outcome measure will be the difference in mean fold-change in urinary KIM-1 from baseline to peak concentration during exposure to tobramycin between the rosuvastatin treated arm and control arm.

### 10.2. Secondary outcomes

1. Difference in serum concentration of creatinine and eGFR during tobramycin exposure between rosuvastatin treated arm and the control arm.
2. Difference in other urinary and plasma biomarkers of renal injury during tobramycin exposure between rosuvastatin treated arm and the control arm.
3. Difference in serious adverse events between rosuvastatin treated arm and the control arm.
4. Difference in tobramycin concentrations between rosuvastatin treated arm and the control arm to identify any pharmacokinetic interaction between rosuvastatin and the tobramycin.
5. Difference in Forced Expiratory Volume in 1 second (FEV1) and C-Reactive Protein, between rosuvastatin treated arm and the control arm to identify any pharmacodynamics interaction between rosuvastatin and the tobramycin.
6. Relationship between plasma rosuvastatin concentrations achieved in children randomised to the intervention arm and change in urinary KIM-1.
7. Difference in biomarkers of *Pseudomonas aeruginosa* between rosuvastatin treated arm and the control arm.

## 11. Determination of Sample Size

PROteKT aims to recruit 50 patients. The sample size calculation can be found in section 9.4 of the protocol.

## 12. Study Framework

The overall objectives for each of the study outcomes (primary and secondary) is to test the superiority of rosuvastatin compared with no intervention.

## 13. Confidence Intervals, p-values and Multiplicity

All applicable statistical tests will be two-sided and will be performed using a 5% significance level; confidence intervals presented will be 95%. No adjustment for multiplicity will be used.

# 14. Timing and Objectives of Interim and Final Analyses

## 14.1. Interim monitoring and analyses

The Independent Data and Safety Monitoring Committee (IDSMC) will meet at least annually as stated within section 5.3 of the IDSMC Charter; the first meeting tookplace after the first 20 patients had been recruited, the second meeting took place after a total of 40 patients had been recruited. There are no formal interim analyses planned at any time during PROteKT. A separate SAP has been written for the IDSMC reports.

## 14.2. Final analysis

The final analysis will take place at the end of the trial which is defined to be the date on which data for all participants has been finalised and entered onto the database with data entry privileges withdrawn (data lock).

# 15. Disposition of Participants

## 15.1. Screening, eligibility and recruitment

Screening logs will be summarised by site in a table detailing:

i) the number of patients who were assessed for eligibility at the screening visit,

ii) those who met the study inclusion criteria at screening (expressed as a frequency and a % with the denominator being i),

iii) those who did not meet the study inclusion criteria at screening (expressed as a frequency and a % with the denominator being i),

iv) those who were eligible at screening and consent obtained, (expressed as a frequency and a % with the denominator being ii),

v) those who were eligible at screening and consent not obtained, (expressed as a frequency and a % with the denominator being ii),

vi) those who provided consent but were not randomised (expressed as a frequency and a % with the denominator being iv),

vii) those who provided consent and were randomised (expressed as a frequency and a % with the denominator being iv).

Reasons for ineligibility will be summarised by site and overall in a table with the following categories for reasons:

1. Patient not within required age range
2. Unable to take tablets
3. Existing treatment with a statin
4. Previous adverse reaction to a statin
5. Co-enrolment in other drug trials or completion of a previous CTIMP within last 30 days
6. Previous randomisation in PROteKT
7. Patient taking excluded con med (see protocol)
8. Female participant who is pregnant or lactating (or refusing a pregnancy test if of child bearing potential)
9. Patient of Asian ancestry (Japanese, Chinese, Filipino, Vietnamese, or Korean)
10. Patient with renal disease (eGFR<60ml/min/1.73m$^2$, using the Schwartz formula, in the 6 months preceding screening)
11. Patient with current elevation in transaminases exceeding 3 x ULN
12. Family or personal history of hereditary muscular disorders
13. Patient with myopathy
14. Patient has a history of or active alcohol abuse
15. Patient has hypothyroidism
16. Patient has galactose intolerance, Lapp lactase deficiency, glucose-galalactose malabsorption
17. Patient is Hepatitis C+ or HIV+
18. Other reason considered ineligible.

Frequencies will be presented along with percentages using the denominator as iii).
Reasons for consent declined will be summarised by site and overall in a table with the following categories:

1. Does not want to consent (unwilling to provide reason)
2. Unwilling/unable to comply with study requirements
3. Does not wish to consent for other reason (reasons will be reported).

Frequencies will be presented along with percentages using the denominator as v).
A recruitment summary table will be presented showing the following for each centre: centre code, hospital name, dates site opened/closed to recruitment, total number of months open to recruitment, planned total recruitment and total number randomised.

A CONSORT flow diagram [4] will be used to summarise the number of patients who were:

- assessed for eligibility at screening
    - eligible at screening
    - ineligible at screening*

- eligible and randomised
- eligible but not randomised*
- received the randomised allocation
- did not receive the randomised allocation*
- lost to follow-up*
- discontinued the intervention*
- randomised and included in the primary analysis
- randomised and excluded from the primary analysis*

*reasons will be provided.

### 15.2. Post randomisation discontinuations

Every effort will be made to collect follow-up data even if participants have withdrawn from trial intervention. In some cases it may not be possible to continue follow-up of trial participants due to transfer to a non-participating centre, loss to follow-up etc. in which case the withdrawal form will be completed.

Withdrawals from treatment and/or trial will be presented as line listings detailing:

- Randomisation number
- Hospital name
- Date of randomisation
- Date of discontinuation of tobramycin
- Date of discontinuation of rosuvastatin if applicable
- Who made the decision to withdraw participant from trial:
  - Clinician
  - Participant /parent/guardian
  - Clinician and participant
- Reason for discontinuation:
  - Death
  - Lost to follow-up
  - Transfer to a non-participating centre
  - Withdrawal of consent for follow-up
  - Other reason (specify)
- Level of withdrawal (from treatment and/or trial).

## 16. Protocol Deviations

Possible protocol deviations will be defined in GE010TEM01 Monitoring Plan and specified as minor or major.
Protocol deviations will be classified and signed-off using ST001TEM03 Protocol Deviations and Population Exclusions template prior to requesting the treatment allocations and any analysis being performed. The number (and percentage) of patients with at least one major/minor/all protocol deviation will be summarised by site and in total by treatment group. The patients that are included in the intention to treat (ITT) analysis data set will be used as the denominator to calculate the percentages. No formal statistical testing will be undertaken.
Patients to be excluded from analysis populations will be defined in template ST001TEM04: Protocol deviations and data set definitions template agreed and approved prior to any release of randomisation code.

# 17. Unblinding

Not applicable since PROteKT is an open label study. The laboratory analysts will remain blinded throughout the duration of the trial.

# 18. Efficacy Evaluations

## 18.1. Data Sets Analysed

The principle of intention-to-treat, as far as is practically possible, will be the main strategy of the analysis adopted for the primary outcome and all the secondary outcomes. These analyses will be conducted on all randomised participants, in the group to which they were allocated, and for whom the outcome(s) of interest have been observed/measured. No imputations will be made.
No subgroup analyses will be performed.
The membership of the analysis set for each outcome will be determined and documented and reasons for participant exclusion will be given prior to the blind being broken and the randomisation lists being requested.
All numeric values will be presented to two decimal places.

## 18.2. Demographic and Other Baseline Characteristics

Descriptive statistics will be reported overall and split by treatment group (rosuvastatin and control). Categorical data will be summarised by numbers and percentages. Continuous data will be summarised by mean, standard deviation, median, inter-quartile range and range. Variables to be included in the table are as follows:

| **Patient demographics** | **CRF** |
|---|---|
| Number of patients randomised | Baseline |
| Age (overall and split by required categories for EudraCT database:<br>Gestational age <37 weeks; 0-27 days; 28 days-23 months; 2-11 years; 12-17 years; 18-64 years years; 65-84 years; 85+) | Baseline |
| Gender (male/female) | Baseline |
| Height (cm) | Baseline |
| Weight (kg) | Baseline |
| Ethnic origin | Baseline |
| **Disease characteristics** | **CRF** |
| Urinary KIM-1 (ng/mg Cr) | Lab data |
| Urinary NGAL (ng/mg Cr) | Lab data |
| Serum creatinine (µmol/L) | Baseline |

| | |
|---|---|
| eGFR (mL/min/1.73m$^2$) | Baseline |
| Aspartate transaminase (iu/L) | Baseline |
| Alanine transaminase (iu/L) | Baseline |
| HDL cholesterol (mmol/L) | Baseline |
| LDL cholesterol (mmol/L) | Baseline |
| Total cholesterol (mmol/L) | Baseline |
| Triglycerides (mmol/L) | Baseline |
| Creatine kinase (iu/L) | Baseline |
| C Reactive Protein (mg/L) | Baseline |
| FEV in 1 second | Baseline |
| FEV in 1 second (% predicted) | Baseline |

### 18.3. Compliance with treatment

Patients that withdrew from treatment will be presented as line listings detailing: randomisation number, site, date of randomisation, date of discontinuation, reason for discontinuation, further details. The number and percentage of completed diaries will be presented overall and split by centre and by treatment group. The proportion of doses of tobramycin reported as not given or not known will be presented averaged across all patients overall and split by centre and by treatment group. The proportion of doses of rosuvastatin reported as not given or not known will be presented averaged across all patients overall and split by centre. Data will be summarised by mean, standard deviation, median, inter-quartile range, minimum and maximum.

### 18.4. Analysis of primary outcome

The primary outcome is the difference in mean fold-change in urinary KIM-1 from baseline to peak concentration during exposure to tobramycin between the rosuvastatin treated arm and the control arm. Urinary KIM-1 will be normalised to urinary creatinine by dividing the KIM-1 value by the corresponding urinary creatinine value.

### 18.4.1. Derivation

Urine samples will be collected daily during exposure to IV tobramycin.

### 18.4.2. Analysis

The primary outcome will be analysed using the method of analysis of covariance (ANCOVA). The outcome measure will be mean log-transformed fold-change of normalised KIM-1 (ng/mgCr) calculated by dividing the peak value, corresponding to the 'highest value' of normalised KIM-1 during exposure to tobramycin, by the baseline normalised KIM-1 value for each participant. The explanatory variables will be treatment group and baseline normalised KIM-1 value. Although randomisation is stratified by centre, due to the small numbers of expected recruitment in each centre, it will not be included as a covariate in the analyses as this could lead to unreliable estimates and p-values which are either too large or too small.
.The assumptions underlying the ANCOVA model will be explored. Normality of residuals will be checked by inspection of histograms and Q-Q plots. If residuals are not normally distributed the non-parametric Mann-Whitney U-test will be used. The assumption of homoscedasticity will be assessed by inspection of scatter plots of the residuals against the fitted values. Where scatter plots suggest non-constant residual variance, sandwich estimators will be used to estimate standard errors and 95% confidence intervals.

## 18.5. Analysis of Secondary Outcomes

The analyses of secondary outcomes will use the method of linear mixed effects models. These models take into account the serial nature of the data and account for correlations within measurements at different time points for the same patient. The total number of patients contributing at each time point will be reported.

### 18.5.1. Change in serum concentration of creatinine and eGFR during tobramycin exposure between rosuvastatin arm and control arm

#### 18.5.1.1. Derivation

A serum sample will be taken at the baseline visit T0, T+1, T+8 and T+13 days (or final day of tobramycin treatment if earlier than T+13), final day of tobramycin (if later than T+13) during tobramycin exposure and at any unscheduled visits, where T is day of randomisation. This will be assessed for serum creatinine and results will be provided from each local laboratory. Height is recorded at T0, T+8 and T+13 days.

The Schwartz formula will be used to calculate eGFR as follows:

eGFR (Schwartz) =40*height (cm) / Serum or Plasma Creatinine (μmol/L).

Creatinine (serum or plasma) will be recorded in the Blood Results section of Case Report Form 3: Follow-up (Treatment Phase). Height (cm) is also recorded on this form.

#### 18.5.1.2. Analysis

Serum concentration of creatinine and eGFR will be the outcome variables in turn. An interaction between time (as a categorical variable) and treatment will be included in a model which will give estimates of the treatment effect at each time point. Mean and individual profile plots, split by treatment group, will be presented.

### 18.5.2. Change in other urinary and plasma biomarkers of renal injury during tobramycin exposure between rosuvastatin arm and control arm

#### 18.5.2.1. Derivation

Plasma samples will be taken at the baseline visit T0, T+1 days, T+8 days (between days T+7 and T+9), T+13 days (or final day of tobramycin treatment if earlier than T+13) , final day of tobramycin (if later than T+13) during tobramycin exposure and at any unscheduled visits. Urine samples will be collected daily during exposure to IV tobramycin.

These samples will be assessed and results on biomarkers will be provided. Urinary NGAL will be measured using a validated assay at the University of Liverpool and will be normalised to urinary creatinine by dividing the NGAL value by the corresponding urinary creatinine value.

#### 18.5.2.2. Analysis

A linear mixed model with log-transformed normalised NGAL (ng/mgCr) as the outcome variable will be used. The model will include an interaction term between treatment and time. Mean and individual profile plots, split by treatment group, will be presented.

### 18.5.3. Difference in tobramycin concentrations between rosuvastatin treated arm and the control arm to identify any pharmacokinetic interaction between rosuvastatin and tobramycin

#### 18.5.3.1. Derivation

Tobramycin doses are taken up to three times daily. A blood sample to measure tobramycin concentrations will be taken on T+1, T+8 and T+13 days (or final day of tobramycin treatment if earlier than T+13), final day of tobramycin (if later than T+13) during tobramycin exposure and at any unscheduled visits, where T is time of randomisation. These will be captured on Case Report Form 3: Follow-up (Treatment Phase). It will also be recorded if the sample was not taken with a reason.

#### 18.5.3.2. Analysis

Tobramycin concentration will be modelled initially using a one-compartmental, non-linear, pharmacokinetic mixed model.
Suppose subject $S_i, i = 1, \ldots, n$, is administered doses $d$ at times $t_j, j = 1, \ldots, l$.
The plasma concentration, $C_{ij}(t)$, of the drug for subject $S_i$ at time $t_j$ is denoted by:

$$C_{ij}(t) = \sum_{j=0}^{l} \frac{d_{ij}k_{ei}}{Cl_i(1 - k_{ei})} \left(e^{-k_{ei}(t-t_{ij})}\right) \exp(\varepsilon_{ij})$$

$Cl_i$ = clearance for subject $S_i$;
$k_{ei}$ = elimination coefficient for $S_i$;
$\varepsilon_{ij}$ = random error term.
Since there are no dose variations, the above can be simplified to:

$$C_{ij}(t_j) = \sum_{j=0}^{l} Ce^{-k_{ei}(t_j - \sum_{m=0}^{j-1}(t_{m+1} - t_m))}$$

$C$ is the exponential coefficient for subject $i$.
Model parameters can be fit using a non-linear regression model.
Area under the concentration curve up to time $t$ ($AUC_{0\text{-}t}$) will be estimated as well as the maximum concentration ($C_{max}$) and the time of maximum concentration $T_{max}$. AUC will be estimated using the trapezium rule.
An ANCOVA model will be used to compare the AUC, $C_{max}$ and $T_{max}$ in both treatment groups. These models will be adjusted for sex, age and BMI.

### 18.5.4. Difference in Forced Expiratory Volume in 1 second (FEV1) and C-Reactive Protein, between rosuvastatin treated arm and the control arm to identify any pharmacodynamic interaction between rosuvastatin and tobramycin

#### 18.5.4.1. Derivation

Forced Expiratory Volume in 1 second (FEV1) will be measured locally during study visits at baseline and time points T+8, T+13 (or final day of tobramycin treatment if before T+13) and final day of treatment (if later than T+13). All blood samples taken at study visits will be analysed for C-Reactive Protein.

#### 18.5.4.2. Analysis

To identify pharmacodynamic interactions, FEV1 and C-reactive protein will be outcome variables in a linear mixed model in turn. The covariates will be treatment group and time; an interaction between time and treatment will also be included. Mean and individual profile plots, split by treatment group, will be presented.

### 18.5.5. Relationship between plasma rosuvastatin concentrations achieved in children randomised to the intervention arm and change in urinary KIM-1

#### 18.5.5.1. Derivation

See section 17.5.2.1 for information on urine and plasma samples. To assess rosuvastatin compliance the diary will be cross-checked with the rosuvastatin concentrations to check that participants who have recorded taking rosuvastatin doses have rosuvastatin concentration not equal to zero.

#### 18.5.5.2. Analysis

Rosuvastatin concentrations and urinary KIM-1 measurements will be summarised at each time point for the rosuvastatin group.

Scatter plots of rosuvastatin concentrations against reduction in log-transformed KIM-1 from baseline will be created for each time point.

### 18.5.6. Difference in biomarkers of *Pseudomonas aeruginosa* between rosuvastatin treated arm and the control arm

#### 18.5.6.1. Derivation

See section 17.5.2.1 for information on urine and plasma samples.

#### 18.5.6.2. Analysis

Each relevant biomarker of *Pseudomonas aeruginosa* will be the outcome variable in turn in the mixed models. Interactions between time and treatment will be included. The specific biomarkers to be assessed have not yet been defined. Only those identified to have *Pseudomonas aeruginosa* will be included in this analysis.

# 19. Missing data and withdrawals

Line listings of patient withdrawals from follow-up with reasons will be presented, overalland split by treatment arm. The numbers of urine sample information sheets returned, urine samples transferred from local site to central lab, urine samples received by the central lab and urine samples viable for analysis will be presented overall and split by centre. Line listings of the number of urine samples expected (i.e. the total number of days on tobramycin treatment), the number of samples viable for analysis and the number of missing samples will be presented.

As much information as possible will be collected about the reasons for missing outcome data and this will be used to inform sensitivity analyses:

1. The difference from baseline to KIM-1 value on final day of tobramycin treatment.
2. The primary analysis will be repeated for those who returned at least 50% of samples only.
3. For those missing a baseline urine sample, the baseline KIM-1 value will be imputed as the mean KIM-1 value over all baseline KIM-1 values.

Reasons for dropouts will be recorded in each treatment arm; if loss to follow-up appears to be informative, i.e. the patient drops out of the study for reasons related to the study then joint modelling of the longitudinal data and the time to dropout from the study will be used to analyse the secondary endpoints.

# 20. Additional analyses

A secondary analysis of urinary KIM-1 will investigate the difference in area under the curve (AUC) of urinary KIM-1 during exposure to IV tobramycin between the rosuvastatin treated arm and control arm.

Log transformed NGAL will also be analysed using the methods for the primary outcome analysis of KIM-1 and area under the curve.

Blind review of the aggregrated data was undertaken by a statistician independent to the PROteKT trial. The following recommendations were made:

- Addition of a sensitivity analysis including centre as a random effect
- Addition of sensitivity analyses for the primary and secondary outcomes excluding values greater than the upper quartile plus 1.5 times the interquartile range (IQR) or lower than the lower quartile minus 1.5 times the IQR.

# 21. Safety Evaluations

Adverse events will be categorised according to severity as “Mild”, “Moderate”, or “Severe”. They will also be classified in relation to the causality with the treatment as “Unrelated”, “Unlikely”, “Possibly”, “Probably”, and “Almost certainly”. Full details on the definition and classification of these adverse events are presented in section 10 of the protocol.

## 21.1. Data sets analysed

For the safety analysis, patients will be analysed according to which treatment was received in order to accurately represent the adverse effects of rosuvastatin. Participants ARs/SAEs will be included in

the treatment group they were actually receiving at the time of AR/SAE onset to take into account any patients that crossed over from one group to another.

### 21.2. Presentation of the data

All adverse reactions (ARs) and serious adverse events (SAEs) reported by the clinical investigator will be presented mutually exclusively in a table. The number (and percentage) of patients experiencing each AR/SAE will be presented for each treatment arm categorised by severity (mild, moderate, severe). For each patient, only the maximum severity experienced of each type of AR will be displayed. The number (and percentage) of occurrences of each AR/SAE will also be presented for each treatment arm. No formal statistical testing will be undertaken. SAEs will be presented as line listings.

## 22. Quality Control

To ensure quality control, an independent statistician will follow this SAP to independently program the primary analysis and safety data from the raw data. Any discrepancies found will be discussed with the trial statistician to resolve. No programming will be shared or shown between the statisticians. The independent statistician will also check the report against their output obtained from the statistical software.

## 23. References


1. International Conference on Harmonisation. Topic E3 Structure and content of clinical study reports.(CPMP/ICH/137/95). 1995
2. International Conference on Harmonisation. Topic E9 Statistical Principles for Clinical Trials (CPMP/ICH/363/96). 1998.
3. Guidance on Statistical Analysis Plans.
4. Schulz KF, Altman DG, Moher D, for the CONSORT Group. CONSORT 2010 Statement: updated guidelines for reporting parallel group randomised trials. BMJ 2010;340:c332.
5. Beringer PM, Vinks AATMM, Jelliffe RW, Shapiro BJ. Pharmacokinetics of Tobramycin in Adults with Cystic Fibrosis: Implications for Once-Daily Administration. Antimicrobial Agents and Chemotherapy. 2000 Apt; 44(4): 809-813.

# Supplemental Tables

## Supplemental Table 1: Treatment compliance

| | Number of days tobramycin taken | | | Number of days rosuvastatin taken |
|---|---|---|---|---|
| | Control | Rosuvastatin | Overall | |
| Number of participants[1] | 25 | 20 | 45 | 20 |
| Mean (SD) | 13.28 (1.24) | 12.60 (2.85) | 12.98 (2.12) | 12.65 (2.74) |
| Median (IQR) | 14 (13, 14) | 14 (12, 14) | 14 (13, 14) | 14 (12, 14) |
| Min, Max | 9, 15 | 3, 15 | 3, 15 | 3, 14 |

[1] Participants only included if they returned a treatment diary and did not withdraw at baseline.

## Supplemental Table 2: Sensitivity analyses for the primary outcome

| Sensitivity analysis | Treatment group | N[1] | Estimated geometric mean fold-change | Estimated mean treatment difference[2] | 95% CI | P-value |
|---|---|---|---|---|---|---|
| Log-transformed mean fold change of KIM-1[3] from baseline to last day of treatment | Control | 24 | 1.85 | - | - | - |
| | Rosuvastatin | 20 | 2.00 | 1.08 | 0.87, 1.35 | 0.48 |
| Log-transformed mean fold change of KIM-1[3] from baseline to peak, excluding those who had returned less than 50% of samples | Control | 23 | 1.89 | - | - | - |
| | Rosuvastatin | 19 | 2.03 | 1.07 | 0.68, 1.34 | 0.52 |
| Log-transformed mean fold change of KIM-1[3] from baseline to peak, including participants with a missing baseline sample by imputing their baseline result as the mean value of all observed baseline results. | Control | 27 | 2.10 | - | - | - |
| | Rosuvastatin | 21 | 1.35 | 0.64 | 0.37, 1.10 | 0.10 |
| Log-transformed mean fold change of KIM-1[3] from baseline to peak, including centre as a random effect | Control | 24 | 1.82 | - | - | - |
| | Rosuvastatin | 20 | 1.99 | 1.09 | 0.89, 1.34 | 0.38 |

| | | | | | | |
|---|---|---|---|---|---|---|
| Log-transformed mean fold change of KIM-1[3] from baseline to peak, excluding normalised KIM-1 results greater than the upper quartile plus 1.5 times the IQR[4] or lower than the lower quartile minus 1.5 times the IQR | Control | 18 | 1.89 | - | - | - |
| | Rosuvastatin | 20 | 1.92 | 1.02 | 0.83, 1.24 | 0.85 |

[1]3 in the control and 1 in the intervention arm did not have baseline urine samples and were excluded; 2 in the intervention arm withdrew at baseline before commencing treatment and were excluded.

[2]Adjusted for baseline normalised KIM-1.

[3]Normalised to urinary creatinine.

[4]IQR=Interquartile range

## Supplemental Table 3: Area under the curve analysis results

| Outcome | Treatment group | N | Mean (SD) AUC of normalised KIM-1 (ng/mgCr) | Estimated mean treatment difference | 95% CI | P-value |
|---|---|---|---|---|---|---|
| KIM-1[1] | Control | 27 | 23.05 (33.02) | - | - | - |
| | Rosuvastatin | 21 | 10.65 (6.11) | 12.41 | -0.89, 25.70 | 0.07 |
| NGAL[1] | Control | 27 | 1139.4 (1106.1) | - | - | - |
| | Rosuvastatin | 21 | 581.6 (630.8) | 557.8 | 46.5, 1069.2 | 0.03 |

[1]Normalised to urinary creatinine.

## Supplemental Table 4: NGAL ANCOVA model results

| Log-transformed mean fold change from baseline to peak NGAL normalised to urinary creatinine | N[1] | Estimated geometric mean fold-change | Estimated mean treatment difference[2] | 95% CI | P-value |
|---|---|---|---|---|---|
| Control | 24 | 8.90 | - | - | - |
| Rosuvastatin | 20 | 4.99 | 0.56 | 0.27, 1.15 | 0.11 |

[1]3 in the control and 1 in the intervention arm did not have baseline urine samples and were excluded; 2 in the intervention arm withdrew at baseline before commencing treatment and were excluded.

[2]Adjusted for baseline normalised NGAL.

## Supplemental Table 5: Post-hoc Analysis: Linear mixed model to assess difference in tobramycin concentrations

| Outcome | Treatment group | N | Estimated mean[1] | Estimated mean treatment difference | 95% CI | P-value |
|---|---|---|---|---|---|---|
| Tobramycin concentration | Control | 25 | 1.24 | - | - | - |
| | Rosuvastatin | 20 | 1.47 | -0.24 | -1.49, 1.02 | 0.70 |

[1]Adjusted for time since last dose of tobramycin; included an interaction between visit and treatment group.

## Supplemental Table 6: Relationship between plasma rosuvastatin concentrations and urinary KIM-1

| Visit | N | Mean (SD) rosuvastatin concentration | Mean (SD) KIM-1 normalised to urinary creatinine (ng/mgCr) |
|---|---|---|---|
| T0 | 14 | 0.10 (0.36) | 6.84 (5.00) |
| T+1 | 10 | 3.25 (4.88) | 7.64 (3.46) |
| T+8 | 16 | 1.56 (1.13) | 8.45 (3.81) |
| T+13 | 14 | 1.22 (1.37) | 12.76 (7.45) |
| 4 weeks following treatment cessation | 13 | 0.33 (1.09) | 7.74 (4.23) |

## Supplemental Table 7: AKI by KDIGO criteria

| Participant | Treatment arm | Baseline serum creatinine (μmol/l) | Follow-up serum creatinine (μmol/l) / Visit | KDIGO stage |
|---|---|---|---|---|
| 1 | Control | 30 | 114 / T+8 | Stage 3: Increase ≥3 times from baseline |
| 1 | Control | 30 | 49 / T+13 | Stage 1: Increase ≥1.5 and <2 times from baseline |
| 2 | Control | 30 | 140 / T+1 | Stage 3: Increase ≥3 times from baseline |
| 2 | Control | 30 | 61 / T+8 | Stage 2: Increase ≥2 and <3 times from baseline |
| 2 | Control | 30 | 46 / T+13 | Stage 1: Increase ≥1.5 and <2 times from baseline |
| 3 | Rosuvastatin | 41 | 76 / T+1 | Stage 1: Increase ≥1.5 and <2 times from baseline |
| 4 | Rosuvastatin | 29 | 47 / T+8 | Stage 1: Increase ≥1.5 and <2 times from baseline |
